# An Unsymmetrical, Cyclic Diborene Based on a Chelating CAAC Ligand and its Small‐Molecule Activation and Rearrangement Chemistry

**DOI:** 10.1002/anie.202113947

**Published:** 2021-12-03

**Authors:** Wei Lu, Arumugam Jayaraman, Felipe Fantuzzi, Rian D. Dewhurst, Marcel Härterich, Maximilian Dietz, Stephan Hagspiel, Ivo Krummenacher, Kai Hammond, Jingjing Cui, Holger Braunschweig

**Affiliations:** ^1^ Institute for Inorganic Chemistry Julius-Maximilians-Universität Würzburg Am Hubland 97074 Würzburg Germany; ^2^ Institute for Sustainable Chemistry & Catalysis with Boron Julius-Maximilians-Universität Würzburg Am Hubland 97074 Würzburg Germany; ^3^ Key Laboratory of Green Chemistry & Technology of Ministry of Education College of Chemistry Sichuan University 29 Wangjiang Road Chengdu 610064 P. R. China; ^4^ School of Physical Sciences Ingram Building University of Kent Park Wood Road Canterbury CT2 7NH United Kingdom

**Keywords:** alkylideneborane, carbene, diborene, thermal rearrangement

## Abstract

A one‐pot synthesis of a CAAC‐stabilized, unsymmetrical, cyclic diborene was achieved via consecutive two‐electron reduction steps from an adduct of CAAC and B_2_Br_4_(SMe_2_)_2_. Theoretical studies revealed that this diborene has a considerably smaller HOMO–LUMO gap than those of reported NHC‐ and phosphine‐supported diborenes. Complexation of the diborene with [AuCl(PCy_3_)] afforded two diborene–Au^I^ π complexes, while reaction with DurBH_2_, P_4_ and a terminal acetylene led to the cleavage of B−H, P−P, and C−C π bonds, respectively. Thermal rearrangement of the diborene gave an electron‐rich cyclic alkylideneborane, which readily coordinated to Ag^I^ via its B=C double bond.

## Graphical abstract

A doubly CAAC‐supported unsymmetrical, cyclic diborene was obtained via an in situ, one‐pot synthesis protocol. This diborene features a small HOMO–LUMO gap. The reactivity of this diborene with small molecules, including [AuCl(PCy_3_)], durylborane, white phosphorus (P_4_) and terminal alkynes, as well as the thermal rearrangement chemistry, were investigated.

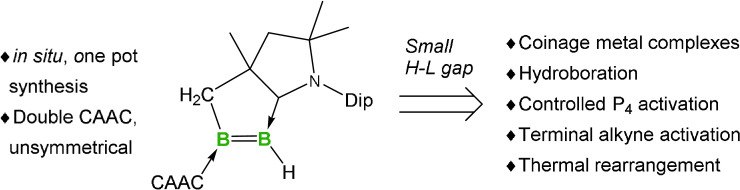

## Introduction

**Figure 1:**
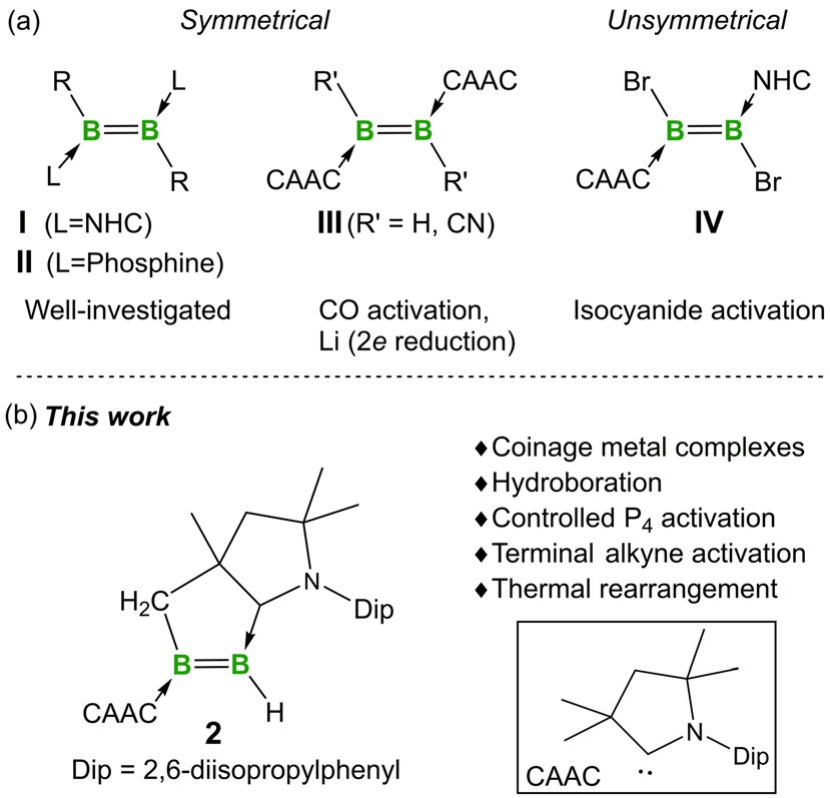
a) Known neutral symmetrical (**I‐III**) and unsymmetrical (**IV**) diborenes supported by NHCs, phosphines and CAACs; b) Present work: a doubly CAAC‐stabilized unsymmetrical diborene.

The last decade has witnessed remarkable advances in the chemistry of neutral diborenes stabilized by Lewis bases, such as NHCs, cyclic (alkyl)(amino)carbenes (CAACs) and phosphines (**I**–**III**) (Figure 1).^[1]^ Intriguingly, it has been established that the bonding properties of the low‐valent B_2_ moieties vary significantly based on the stabilizing ligands.^[2]^ Indeed, it has been revealed that the presence of the stronger π‐accepting and stronger σ‐donating CAAC ligands in diborenes gives rise to smaller HOMO–LUMO (HL) gaps than those of their NHC and phosphine analogs, which holds potential for the activation of enthalpically strong chemical bonds.[^2a^, ^3^] Despite their fundamental importance, CAAC‐supported diborenes are far less established, which is mainly due to the lack of reliable synthetic routes to these species in contrast to the well‐developed protocols for NHC‐ and phosphine‐stabilized diborenes.^[1c]^ For example, the dehalogenative coupling protocol established for the synthesis of NHC‐stabilized diborenes is not applicable to CAAC‐stabilized aryldihaloboranes CAAC⋅BArX_2_ due to their preference for radical or borylene formation under reductive conditions.^[4]^ Most notably, the general synthetic methods utilizing preformed diboranes B_2_X_4_/B_2_R_2_X_2_ as building blocks for NHC‐ and phosphine‐supported diborenes have not yet found success with CAACs.[^3a^, ^5^] Accordingly, it is unsurprising that only two examples of structurally authenticated CAAC‐stabilized diborenes have been described so far.[^3b^, ^6^] The parent diborene (**III**, R′=H) was obtained by either reductive coupling of CAAC⋅BX_2_H (X=Br, Cl) adducts or hydrogenation of a diboracumulene (**V**). In contrast, a sterically encumbered dicyanodiborene (**III**, R′=CN) was generated by thermal rearrangement of an isocyanide adduct of **V**.

In 2017, Kinjo and co‐workers reported that the bonding nature of the B_2_ core in **I** could be fine‐tuned by the incorporation of one CAAC in place of an NHC ligand, providing the first unsymmetrical diborene, **IV**, with a polarized B=B bond.^[7]^ Employing a similar protocol, the same group developed a CAAC‐ and phosphine‐supported diboraallene featuring an unsaturated B−B bond.^[8]^ Given that the desymmetrization strategy enables the construction of more reactive diborene species compared with their symmetrical counterparts,^[9]^ a significant recent interest in this field is the synthesis of unsymmetrical diborenes via stepwise introduction of different supporting ligands to the B_2_ centers of the relative diborane precursors.^[10]^ Moreover, the addition of σ bonds across a B−B triple bond of NHC‐supported diborynes has also been achieved, which allows the simple installation of different anionic substituents on a B_2_ motif.^[11]^ Despite these advances, doubly CAAC‐supported unsymmetrical diborenes have not yet been described, while the chemistry of doubly CAAC‐stabilized diborenes in general is far less developed than those of NHC‐ and phosphine‐stabilized diborenes.[^6b^, ^12^] Herein, we present the synthesis, characterization, and reactivity of a reactive CAAC‐supported diborene based on a highly unusual, in situ‐generated chelating CAAC ligand.

## Results and Discussion

**Scheme 1:**
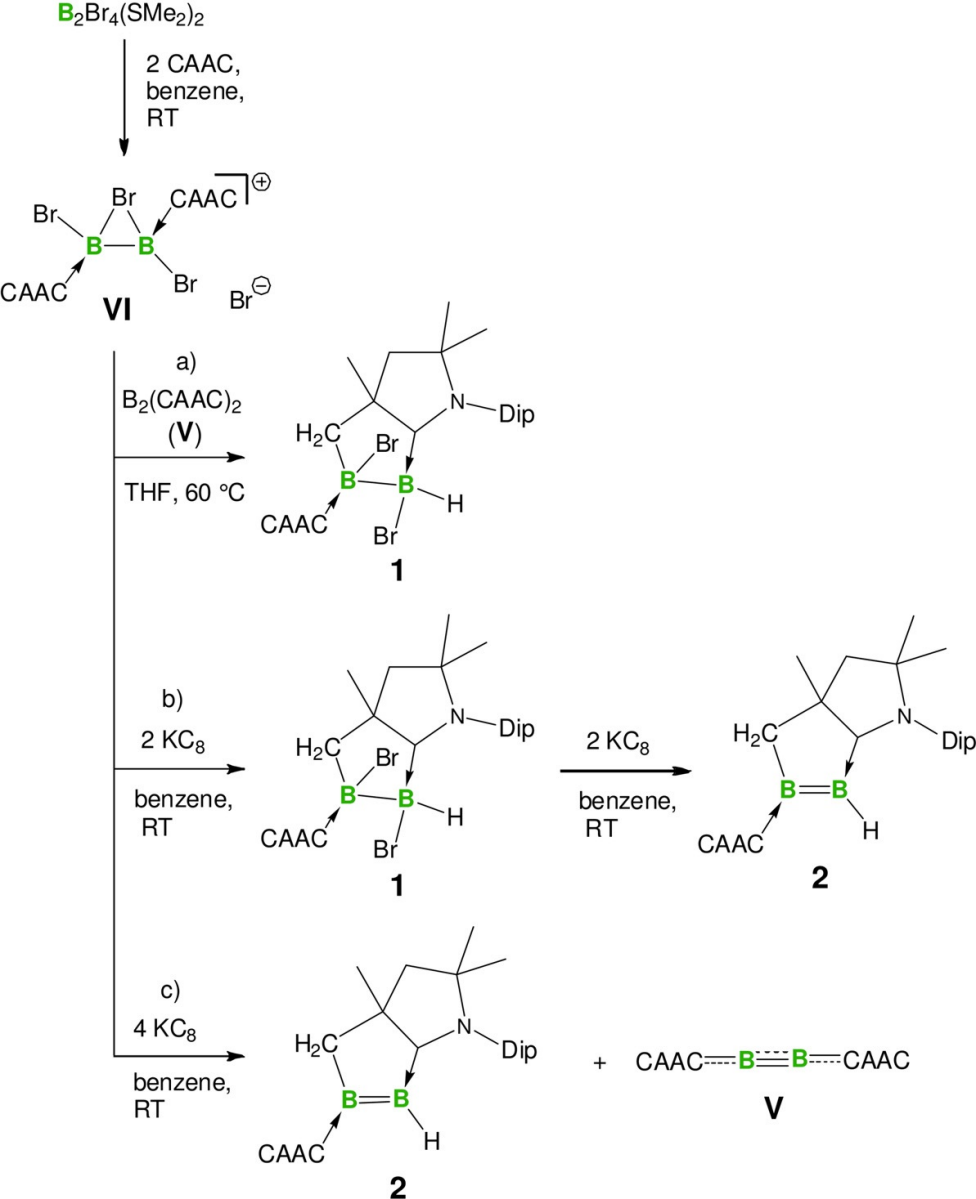
Syntheses of doubly CAAC‐supported unsymmetrical diborene **2**.

**Synthesis of an unsymmetrical CAAC‐stabilized, cyclic diborene and its coordination chemistry**. In previous studies we explored the four‐electron reduction of [B_2_Br_3_(CAAC)_2_]Br **VI** in the presence of sodium naphthalenide in THF at −78 °C, yielding exclusively the diboracumulene **V**.^[13]^ Surprisingly, when **VI** was subjected to two‐electron reduction with KC_8_ in benzene at ambient temperature, the unsymmetrical diborane **1** was obtained in moderate yield (Scheme 1 b). We reasoned that the formation of **1** could be due to the existence of the highly labile transient dibromodiborene B_2_Br_2_(CAAC)_2_ (**VII**), which would provide **1** after rearrangement via addition of a C−H bond from a methyl group of a CAAC unit across the B=B double bond. We hypothesized that dibromodiborene **VII** might be accessible by an alternate approach, via the comproportionation reaction of diboracumulene **V** with **VI**.^[14]^ While only a very slow conversion was noted at lower temperatures, heating a THF suspension of **VI** and **V** to 60 °C provided a green mixture, from which compound **1** was obtained in good yield (Scheme 1 a).

**Figure 2:**
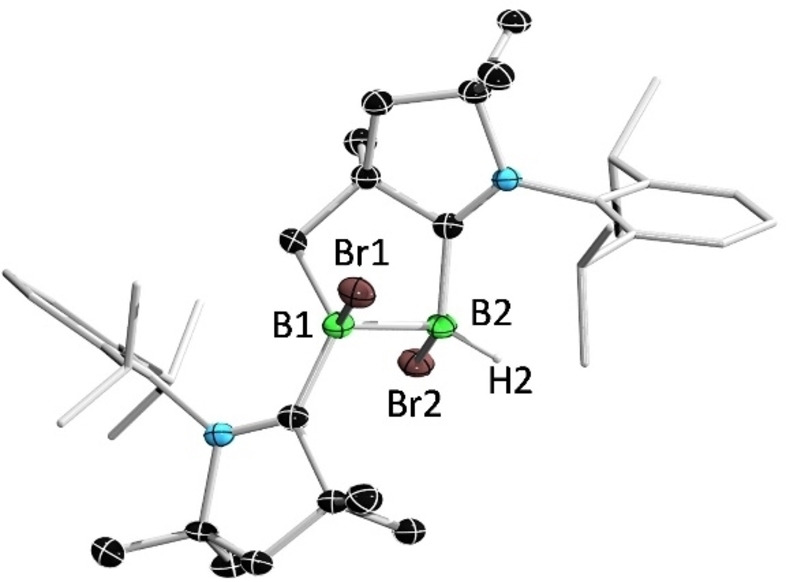
Solid‐state structure of **1**. Ellipsoids are shown at the 50 % probability level. Ellipsoids of peripheral groups and all hydrogen atoms except that bound to B2 have been omitted for clarity.

The ^1^H and ^13^C NMR spectra of **1** indicate the presence of a ca. 1:1 diastereomeric mixture. In the ^11^B NMR spectrum, compound **1** displays two resonances at 1.7 and −10.8 ppm, indicative of two tetracoordinate boron centers. Single‐crystal X‐ray diffraction analysis revealed the presence of a derivatized CAAC unit chelating the diborane B_2_ unit as a monoanionic group (Figure 2).^[15]^

The presence of two bromides and two neutral carbene donors bound to the B_2_ unit of **1** suggested that this could also act as a precursor to a diborene. Accordingly, treatment of **1** with two equivalents of KC_8_ in benzene at ambient temperature afforded a blue solution, which after workup yielded diborene **2** as a dark blue crystalline solid. Interestingly, when **VI** was treated with four equivalents of KC_8_ at room temperature, a reaction mixture containing **2** and **V** was obtained (Scheme 1 c). These results further support the presence of transient dibromodiborene **VII**, which either undergoes rearrangement to give **1** or further two‐electron reduction to provide **V**.

Compound **2** shows two ^11^B NMR signals (48.4 and 43.2 ppm) that are downfield with respect to those of **1** (1.7 and −10.8 ppm). The calculated ^11^B NMR resonances of an optimized structure of **2** at the B3LYP/6‐311G* level of theory (CH_2_ *B*: 51.0 ppm; *B*H: 44.5 ppm) are nearly identical to those observed experimentally (Table S2).

**Figure 3:**
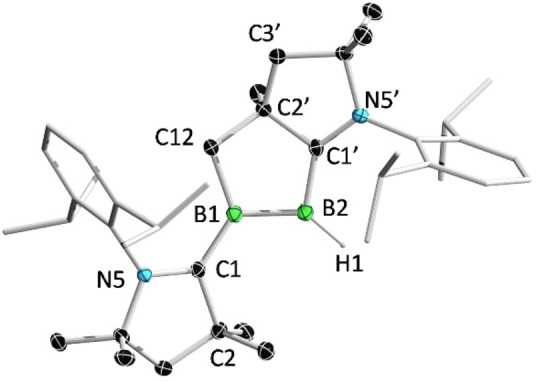
Solid‐state structure of **2**. Ellipsoids are shown at the 50 % probability level. Ellipsoids of peripheral groups and all hydrogen atoms except that bound to B2 have been omitted for clarity.

The solid‐state structure of **2** was unambiguously determined via single‐crystal X‐ray diffraction analysis (Figure 3). The B=B distance (1.633(7) Å) is significantly shorter than that in **1** (1.818(5) Å), but falls at the long end of the range for B=B double bonds reported in base‐stabilized diborenes (1.52–1.63 Å). The C1′−B2 (1.523(9) Å) distance is comparable to those in **III** (R′=H) and **IV**, but considerably shorter than the C1−B1 (1.633(7) Å) distance, indicating much stronger interactions between the B_2_ core and the CAAC_C1′_ ligand.

**Figure 4:**
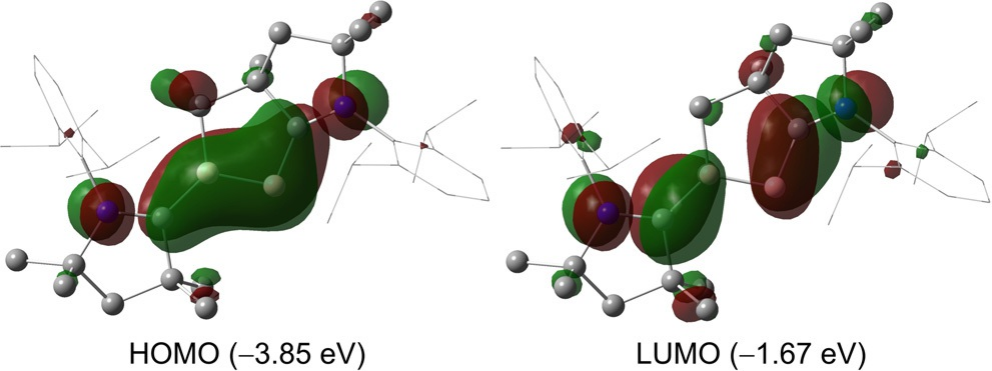
Plots of the HOMO and LUMO of **2**.

To elucidate the electronic features of **2**, we performed density functional theory (DFT) and natural bond orbital (NBO) calculations at the B3LYP/6‐311G* level of theory. The HOMO of **2** mainly corresponds to the B=B π‐bonding orbital with some delocalization to the formally empty p‐orbital of the carbene carbon centers (Figure 4). The LUMO is dominated by π‐type orbitals at the B1−C1 and B2−C1′ bonds, with some contribution from the adjacent N lone pairs. NBO analysis gives a Wiberg bond index (WBI) value of 1.25 for the B−B bond, confirming the multiple bond character of the B−B bond. Interestingly, the calculated H–L gap of compound **2** is relatively small compared with those reported for **I**–**IV** (comparable to that of **III** (R=H)), suggesting its potential in the activation of energetically inert bonds (see Supporting Information, Figure S52).[^2a^, ^3^, ^7^]

**Scheme 2:**
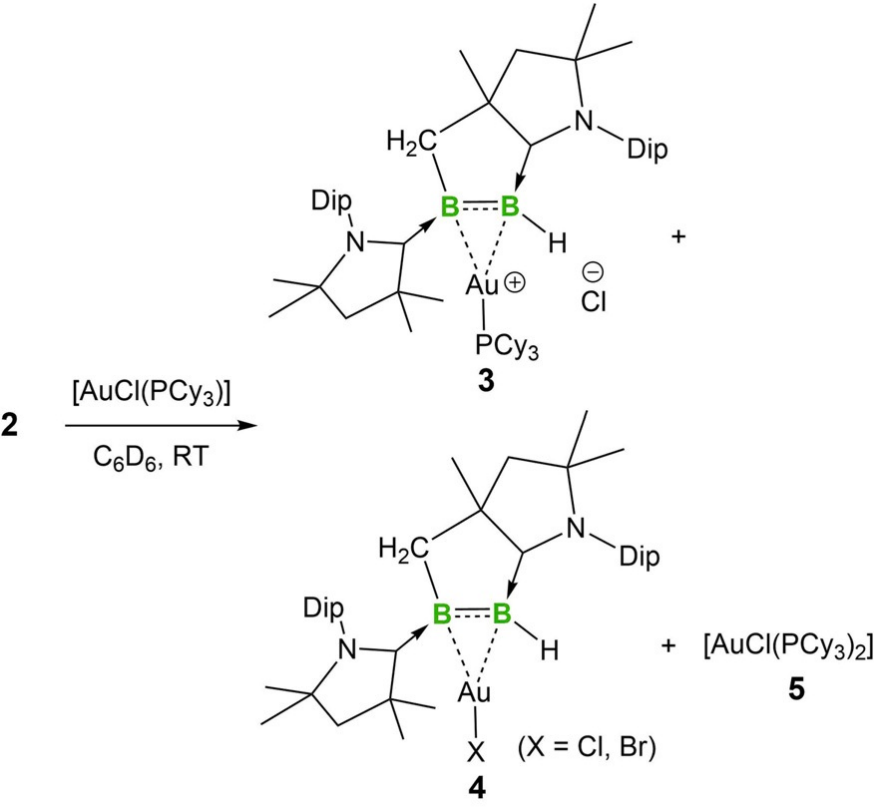
Coordination of **2** to Au^I^.

Considering that previous reactivity studies of CAAC‐stabilized diborenes **II**–**IV** were limited to their reaction with CO, lithium and an isocyanide, we set out to examine the reactions of **2** with coinage metal complexes and small molecules. The reaction of **2** with AgOTf gave a red mixture concomitant with the formation of a Ag mirror owing to the reduction of Ag^I^. When **2** was treated with [AuCl(PCy_3_)] in benzene, the blue color faded over ca. 1 h to afford a red mixture, from which **3** was obtained as red crystals (Scheme 2). After separation of **3** from the mother liquor, **4** was obtained as a red crystalline solid. The ^11^B NMR spectrum of compound **3** displays two broad singlets (37.8, 30.9 ppm) that are upfield compared with those of **2** (48.4, 43.2 ppm).

**Figure 5:**
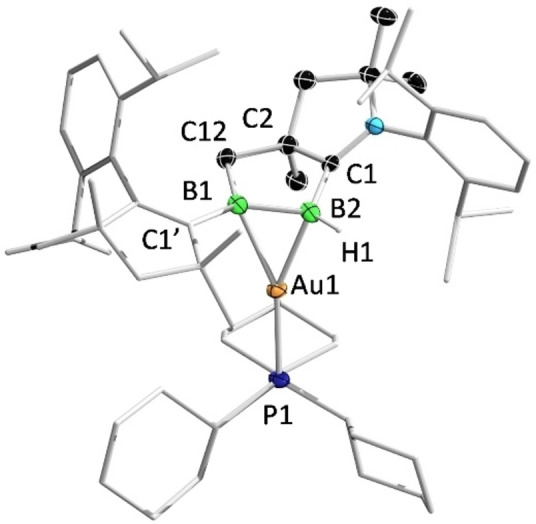
Solid‐state structure of **3**. Ellipsoids are shown at the 50 % probability level. Ellipsoids of peripheral groups, the counterion of **3** and all hydrogen atoms except that bound to B2 have been omitted for clarity.

**Figure 6:**
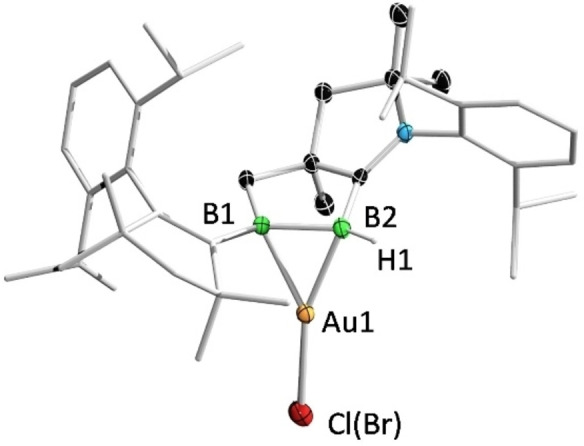
Solid‐state structure of **4**. Ellipsoids are shown at the 50 % probability level. Ellipsoids of peripheral groups and all hydrogen atoms except that bound to B2 have been omitted for clarity.

Single‐crystal X‐ray diffraction analyses revealed the solid‐state structures of **3** and **4**. As shown in Figure 5, the cationic Au^I^ center of **3** is coordinated to the B=B unit in an unsymmetrical η^2^ fashion. The B−B distance of **3** (1.669(4) Å) is slightly longer than the corresponding bond of **2** (1.633(7) Å), which is in turn significantly longer than those (1.58–1.59 Å) in 1,2‐diboraallene‐Au^I^ complexes, the only structurally characterized examples of complexes of unsaturated B−B species with Au^I^.^[16]^ The B−Au distances (B2−Au1: 2.271(3) Å and B1−Au1: 2.354(2) Å) are slightly longer than those (2.21–2.22 Å) found in the aforementioned 1,2‐diboraallene‐Au^I^ complex. Compound **4** co‐crystallized as a mixture of AuCl‐ and AuBr‐containing species, likely due to a halogen exchange between AuCl and KBr from a trace impurity in samples of **2**, as the crystals were grown from an incompletely purified sample before recrystallization (Figure 6). The two species were refined with an occupancy of 0.63(Cl) to 0.37(Br). Compound **4** features a considerably longer B−B bond (1.695(4) Å) than that of **3**. The boron–Au distances (B2−Au1: 2.198(3) Å and B1−Au1: 2.219(3) Å) are slightly shorter than those in **3**, but are comparable to those reported in the aforementioned 1,2‐diboraallene–Au^I^ complex.

**Scheme 3:**
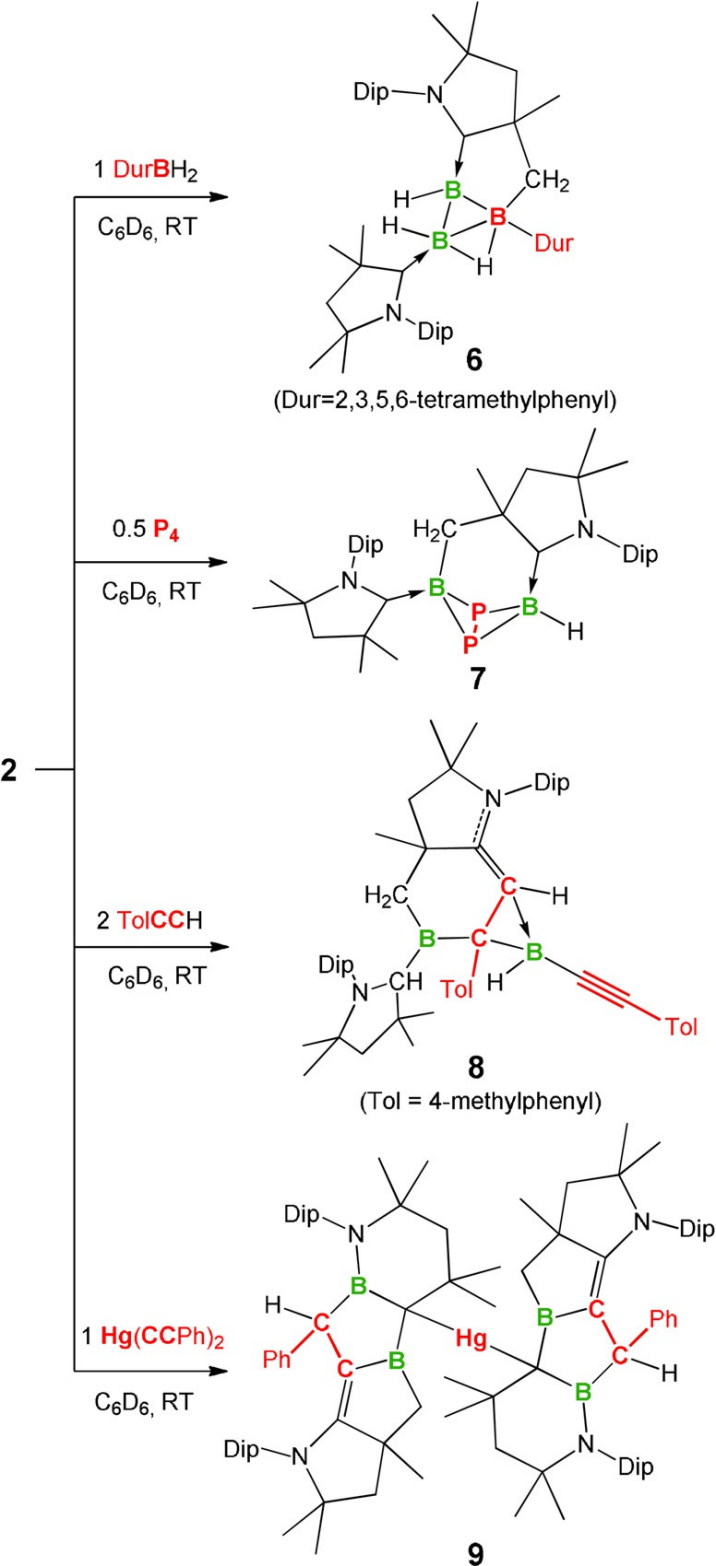
Reactivity of **2**.

**Further reactivity of 2**. With a homoatomic bond dissociation energy between those of carbon and silicon, both of which readily form long and stable chains, boron is a potential candidate for homoatomic bond catenation to provide B−B chains.^[17]^ Despite great efforts, however, these catenation reactions are hampered by cluster and multicenter complex formation due to the intrinsic electron‐deficiency of boron.^[18]^ We have previously demonstrated the catalyst‐free hydroboration and dihydroboration of doubly NHC‐stabilized diborenes, which enabled the formation of electron‐precise B_3_ chains and B_3_ clusters.^[19]^ In view of these diverse B_3_ structures obtained by the hydroboration of diborenes, we examined the reaction of **2** with a hydroborane (Scheme 3).

**Figure 7:**
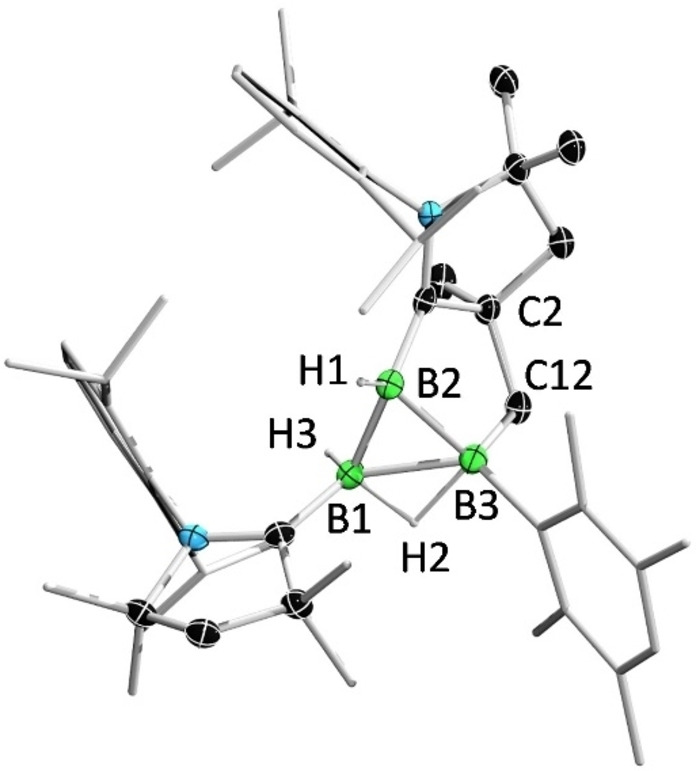
Solid‐state structure of **6**. Ellipsoids are shown at the 50 % probability level. Ellipsoids of peripheral groups and all hydrogen atoms except those bound to boron have been removed for clarity.

Treating a benzene solution of **2** with one equivalent of durylborane (DurBH_2_, Dur=2,3,5,6‐tetramethylphenyl) afforded a red mixture within 15 min, from which single crystals of **6** were obtained by slow evaporation of a benzene solution in a glovebox. Compound **6** displays three ^11^B NMR resonances (7.3, −5.4, −24.3 ppm), suggesting the formation of a nonclassical boron complex. Single‐crystal X‐ray diffraction analysis of **6** established its tricyclic structure with the B_3_ three‐membered ring positioned approximately perpendicular to the B_2_C_3_ five‐membered ring (C12‐B3‐B2‐B1 torsion angle, 101.8(1)°) (Figure 7). The B−B distances (1.787(2), 1.798(2) and 1.890(2) Å) of **6** fall within the typical range of B−B bonds in boron clusters (1.67–2.09 Å).[^19b^, ^20^]

The controlled activation of white phosphorus (P_4_) with reactive low‐valent main‐group compounds has attracted great interest as a potentially environmentally benign route to organophosphorus compounds.^[21]^ To our knowledge, there is only one example of P_4_ activation mediated by a low‐valent boron species, Kinjo's aforementioned 1,2‐diboraallene, which selectively gives B_2_P_4_ and B_4_P_4_ cages.^[22]^ When **2** was treated with P_4_, a red mixture was formed, from which a stoichiometric amount of **7** was obtained as a red solid. Compound **7** shows two broad resonances in its ^31^P NMR spectrum (−119.0, −217.4 ppm), indicating the cleavage of P−P bonds of P_4_ and formation of B−P bonds. The corresponding ^11^B NMR spectrum shows singlet and doublet resonances (−12.9, −21.6 ppm, respectively) that are significantly upfield of those of **2**.

**Figure 8:**
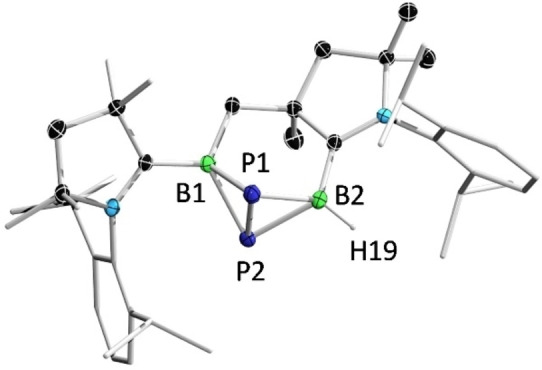
Solid‐state structure of **7**. Ellipsoids are shown at the 50 % probability level. Ellipsoids of peripheral groups and all hydrogen atoms except that bound to B2 have been removed for clarity.

The solid‐state structure of **7** reveals the formation of a B_2_P_2_ butterfly fragment with a B1‐P1‐P2‐B2 torsion angle of 108.4(8)°, which is supported by two CAAC ligands as well as methylene and hydride substituents (Figure 8). In contrast to the reaction of Kinjo's 1,2‐diboraallene with P_4_, which renders B_*n*_P_*m*_ cages with retention of the B−B σ bond, the reaction of **2** with P_4_ leads to four‐electron oxidation and complete cleavage of the B=B double bond.

Polyheterocycles are complex organic polycyclic frameworks involving two or more heterocyclic moieties and constitute an important class of compounds that have been widely utilized in the field of materials science, coordination chemistry, agrochemistry, and most notably, medicinal chemistry.^[23]^ Among these, boron‐containing heterocycles have drawn growing interest because of their potential use as bioactive agents and the peculiar physicochemical properties of boron‐containing benzene analogs.^[24]^ Given that heavier p‐block element alkene and alkyne analogs are capable of activating alkynes via cycloaddition to give a range of ring structures, we examined the reaction of **V** with propyne and acetylene, which readily cleaves the C−C triple bonds to afford aromatic diborete and diborabenzene complexes, respectively.^[2c]^ It was also disclosed that the presence of labile supporting ligands enabled a diborene to react with 2‐butyne under photolytic conditions, yielding a monophosphine‐stabilized homoaromatic 1,3‐dihydro‐1,3‐diborete species. More recently, we reported reactions of NHC‐ and phosphine‐stabilized diborenes with terminal alkynes, from which hydroalkynylation, [2+2] cycloaddition and C−C triple bond scission products were isolated; the outcome was dependent on the nature of the B=B double bonds and the reaction conditions.^[25]^ Inspired by the above‐mentioned advances, we investigated the reaction of **2** towards alkynes.

The reaction of **2** with (4‐methylphenyl)acetylene (TolCCH) in a 1:2 ratio provided **8** as a white solid. In the ^1^H NMR spectrum of **8**, two characteristic resonances for the protons residing on the protonated carbene carbon and the borirane carbon are displayed at 3.69 (singlet) and 2.91 (doublet, *J=*8 Hz) ppm, respectively. In the ^11^B NMR spectrum, two broad peaks for the tri‐ and tetracoordinate boron atoms are observed at 76.7 (very broad) and −13.8 ppm, respectively, which are well reproduced by theoretical calculations (77.5 and −11.2 ppm). Given that the reported C−C triple bond cleavage reactions of diborenes are limited to conventional alkynes, we investigated the reaction of **2** with metal alkynyl complexes. When **2** was treated with an excess of NaCCH, NMR spectroscopy showed no evidence of a reaction. In contrast, reaction of **2** with half an equivalent of bis(2‐phenylethynyl)mercury (Hg(CCPh)_2_) afforded a red‐brown mixture rapidly, from which orange single crystals of **9** were obtained. Complex **9** was isolated only in small amounts and could not be characterized by NMR spectroscopy, perhaps due to the presence of diastereomers arising from the numerous chiral centers in the molecule. Further reactivity studies showed that **2** was capable of activation of 1,4‐diethynylbenzene and 1,3,5‐triethynylbenzene, respectively. However, all attempts to separate the products from the reaction mixtures were unsuccessful.

**Figure 9:**
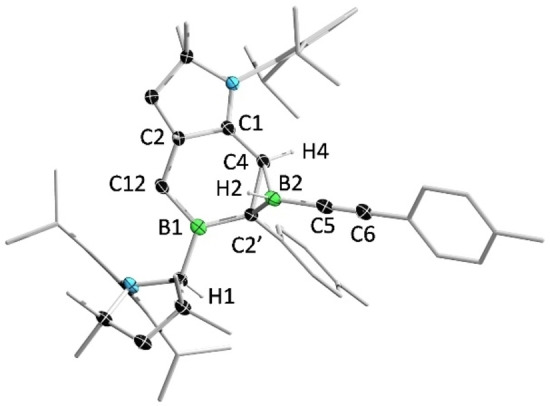
Solid‐state structure of **8**. Ellipsoids are shown at the 50 % probability level. Ellipsoids of peripheral groups and all hydrogen atoms except those bound to B2, C4 and the CAAC carbene center have been removed for clarity.

**Figure 10:**
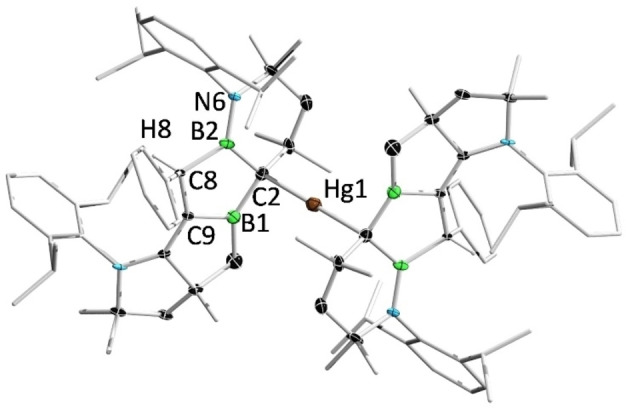
Solid‐state structure of **9**. Ellipsoids are shown at the 50 % probability level. Ellipsoids of peripheral groups and all hydrogen atoms except that bound to C8 have been removed for clarity.

Single‐crystal X‐ray diffraction analyses revealed the solid‐state structures of **8** and **9** (Figure 9 and Figure 10). Compound **8** features a tricyclic ring moiety, comprised of a boron‐ and nitrogen‐doped octahydrocycloprop[*e*]indene derivative, an aroma moiety of interest to the fragrance industry.^[26]^ The B2−C4 distance (1.726(3) Å) is slightly longer than typical B(sp^3^)−C(sp^3^) bond lengths in electron‐precise boron complexes (1.57–1.69 Å),^[27]^ and the C1−C4 bond length (1.395(2) Å) is between those of C−C single and double bonds. These structural features, together with the characteristic ^11^B NMR resonance, imply the presence of a cyclic (alkyl)(amino)olefin (CAAO) donor, which closely resembles N‐heterocyclic olefin ligands.^[28]^

The solid‐state structure of compound **9** is centrosymmetric with two tetracyclic units lying parallel to each other. The bonding of Hg1 and C2 leads to a pyramidal geometry around C2 if the C−Hg bond is disregarded (sum of bond angles: 340.7°).

**Figure 11:**
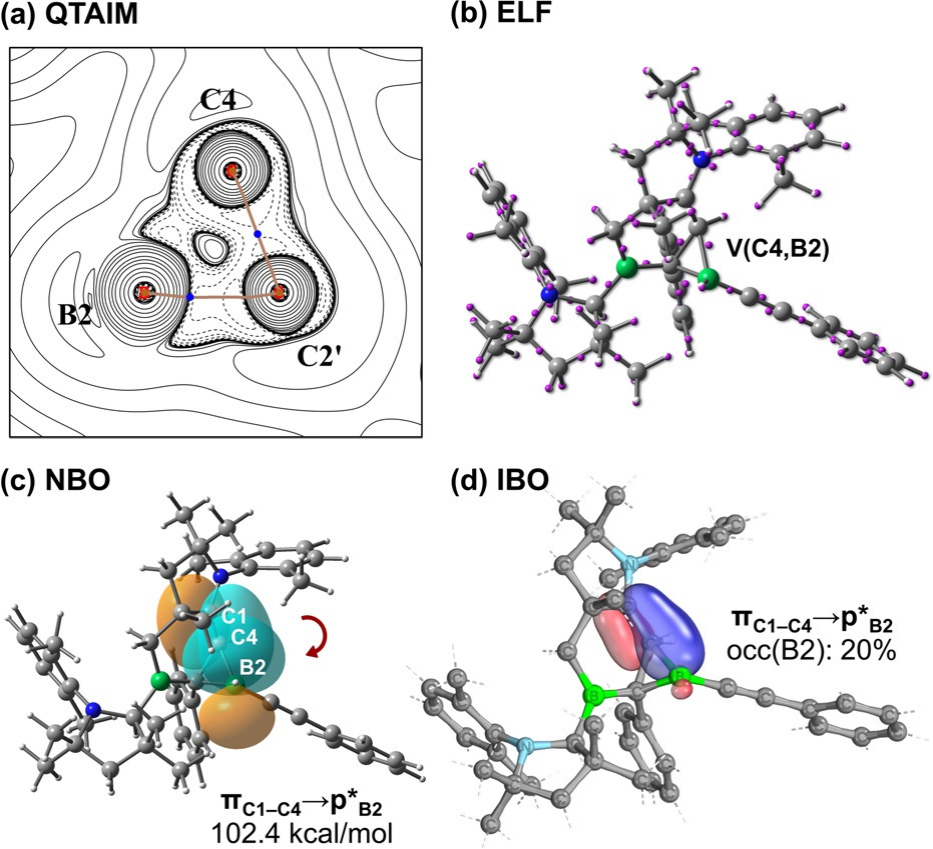
Theoretical results revealing the interactions between a CAAO and B2 in **8′**: a) QTAIM electron density map with relevant bond paths and bond critical points; b) ELF attractors (purple dots); c) NBO π_C1−C4_→p*_B2_ perturbative donor–acceptor interaction (102.4 kcal mol^−1^); d) the π_C1−C4_ IBO and its delocalization to the adjacent B2 center (occupancy at B2: 20 %).

To gain additional insight into the interactions between the CAAO ligand and B2 in **8**, we carried out computational calculations based on distinct, complementary approaches on the simplified structure **8′** (see Table S1b for structure). Within the framework of the quantum theory of atoms in molecules (QTAIM), no (3,−1) bond critical point is found between the B2 and C4 atoms (Figure 11 a), indicating the absence of conventional covalent C−B bonding. Indeed, electron localization function (ELF) calculations reveal a V(C,B) bonding attractor between C4 and B2 (Figure 11 b), but which is not located on the straight line linking the atomic nuclei. Similar peculiar positions of the bonding attractor have previously been reported for some highly strained molecules, including cyclopropane and oxaziridine,^[29]^ and are usually associated with the formation of bent σ bonds.^[30]^ A C4−B2 bonding interaction in **8′** is also supported by Mayer bond order (MBO), WBI, and fuzzy bond order (FBO) calculations, as C4−B2 bond orders of 0.47, 0.49 and 0.56 are found, respectively. Second‐order perturbation energies obtained from NBO computations confirm the presence of interactions between the B2 and C4 nuclei originating mostly from donor–acceptor bonding (E^(2)^=102.4 kcal mol^−1^) from the π(C1−C4) orbital of the CAAO unit to the lone vacant p*_B2_ orbital (Figure 11 c), which shifts their corresponding natural orbital occupancies to 1.67 and 0.46, respectively. This interaction is supported further by intrinsic bond orbital (IBO) calculations, where a π_C1−C4_→p*_B2_ donor‐acceptor interaction is also found (Figure 11 d). Taken together, our computations reveal the presence of C4−B2 bonding interactions, ultimately leading to the stabilization of an unusual three‐membered C_2_B ring involving a neutral CAAO donor ligand and a vicinal π‐acidic boron center.

Given the established dissociation of certain alkenes,^[31]^ and alkene analogs of Al^[32]^ and the other group 14 elements (Si, Ge, Sn, Pb),^[33]^ to form the corresponding carbene and carbene analogs, and the fact that CAACs are capable of stabilizing dicoordinate borylenes,^[4]^ we envisaged that CAAC‐stabilized borylenes could be accessible if the bond dissociation approach could be extended to **2**. This prompted us to investigate the stability of **2** under thermolysis. When a C_6_D_6_ solution of **2** was heated at 80 °C overnight, an orange mixture was obtained, and NMR spectroscopy suggested the stoichiometric conversion of **2** to a new species, from which compound **10** was isolated as an orange solid. In the ^1^H NMR spectrum of **10**, a broad resonance for the B*H* unit is observed at 4.5 ppm, which is upfield relative to that of **2** (5.2 ppm). The ^11^B NMR spectrum appears as two broad resonances at 42.6 and 29.2 ppm, both upfield of those of **2** (48.4 and 43.2 ppm).

**Scheme 4:**
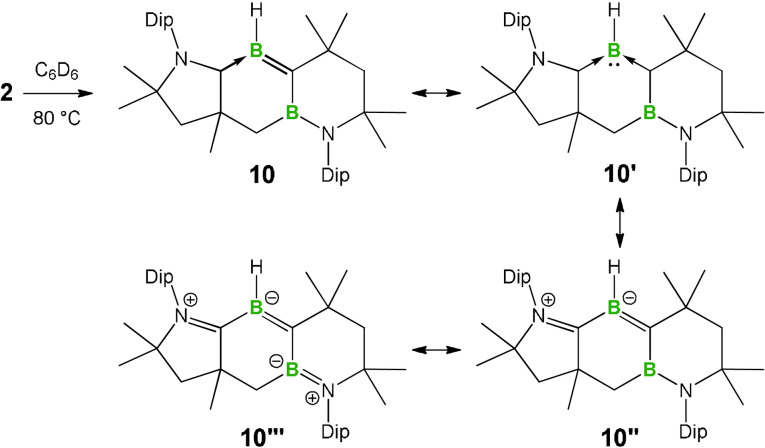
Thermal rearrangement of **2** into **10** and plausible resonance forms thereof.

A single‐crystal X‐ray diffraction analysis of **10** indicated that cleavage of the B=B double bond of **2** had occurred, along with the ring expansion and migration of the non‐tethered CAAC ligand, to furnish a cyclic alkylideneborane, which could also be described as a CAAC and cyclic (alkyl)(boryl)carbene (CABC)‐stabilized borylene (**10′**) (Scheme 4). To gain more information on the thermal cleavage of the B=B double bond, we performed a trapping experiment by heating a C_6_D_6_ solution of **2** under a H_2_ atmosphere. However, compound **10** was again obtained exclusively and no evidence for the homo‐cleavage of the B=B double bond was obtained.

**Figure 12:**
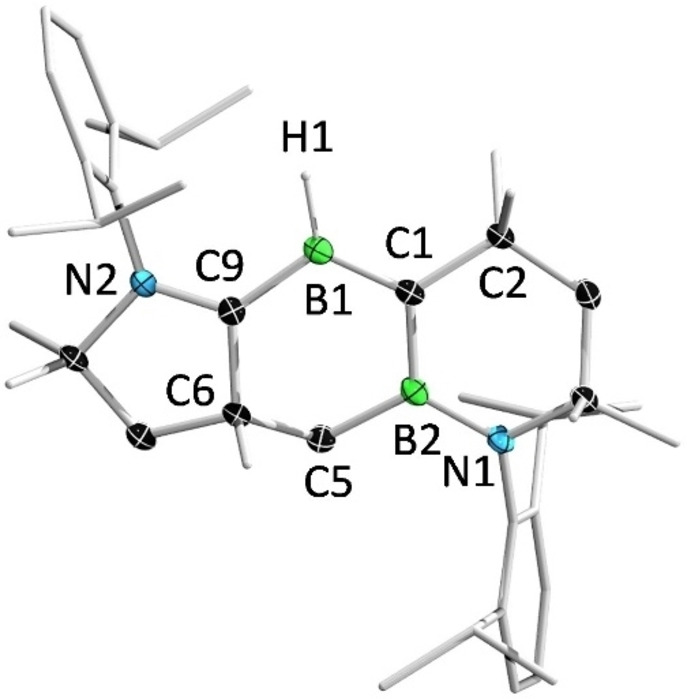
Solid‐state structure of **10**. Ellipsoids are shown at the 50 % probability level. Ellipsoids of peripheral groups and all hydrogen atoms except that bound to B1 have been removed for clarity.

In the solid‐state structure of **10**, the H1, B1, C9, C1, C2 and B2 atoms are nearly coplanar (Figure 12). The B1−C9 distance (1.545(2) Å) is slightly longer than the corresponding distance in **2**. In contrast, the B1−C1 bond length (1.468 (2) Å) is significantly shorter than those of the C_CAAC_−B bonds in **2** (1.523(9) and 1.633(7) Å) but fall in the range of reported B=C double bonds in base‐supported boraalkenes[^2b^, ^3b^, ^34^] (1.43–1.50 Å) and are comparable to those of CAAC‐stabilized borylenes (1.46–1.48 Å).^[35]^

Boraalkenes featuring a dicoordinate boron center and a neutral B=C double bond are formally isolobal and isoelectronic to vinyl cations and have been known for decades.[^34a^, ^36^] Their small‐molecule activation chemistry[^36a^, ^37^] and complexation with transition metals fragments^[38]^ were studied by the groups of Nöth, Berndt and Paetzold. However, these compounds generally either bear deactivating heteroatom substituents at boron or are generated under harsh conditions. In 2015, we reported the unique reactions of **V** with CO and isocyanide, which allowed the formation of bis(carbonyl) and bis(isocyanide) adducts of **V**, respectively, involving CAAC‐supported B=C bonds.[^2b^, ^3b^] Shortly afterward, Kinjo et al. reported the synthesis of a series of 2‐aza‐4‐borabutadienes by triethylborane‐catalyzed carboboration of nitriles.^[34b]^ More recently, Erker and co‐workers documented the construction of NHC‐stabilized cyclic boraalkenes by simple deprotonation of their borenium precursors and demonstrated their cycloaddition reactions with small molecules.^[34c]^

**Scheme 5:**
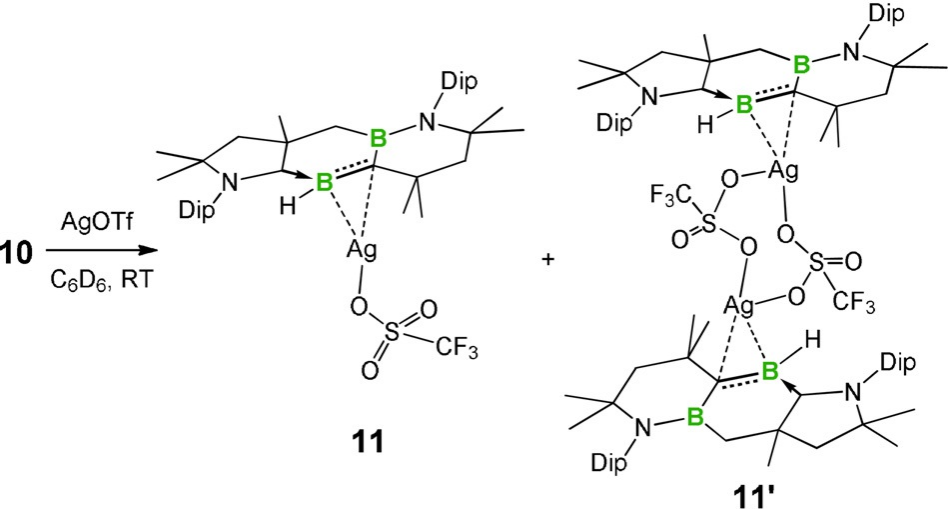
Complexation of **10** with AgOTf.

Despite the above advances, the coordination chemistry of alkylideneboranes is much less developed. Indeed, structurally authenticated metal complexes of alkylideneboranes are limited to Fe^0^,[^38a^, ^38e^] Co^I^,[^38b^, ^38c^] Rh^I^,^[38d]^ and Pt^0[38c]^ examples, which prompted us to examine the coordination chemistry of **10**. When **10** was treated with a stoichiometric amount of AgOTf in benzene, a red suspension formed immediately, from which colorless crystals were obtained by slow evaporation of a benzene solution in a glovebox (Scheme 5). The ^1^H and ^13^C NMR spectra showed evidence for the existence of two isomers (**11**/**11′**) in a 1:1.8 ratio in dichloromethane for the alkylideneborane–Ag^I^ complexes. The ^11^B NMR spectrum displays two broad resonances at 39.9 and 20.8 ppm, which are 2.7 and 8.4 ppm upfield of those in **10**, respectively. Despite multiple attempts, we were unable to separate the two isomers by recrystallization. In contrast, **10** does not form a complex with [AuCl(PCy_3_)], likely due to the steric hindrance around the Au^I^ center.

**Figure 13:**
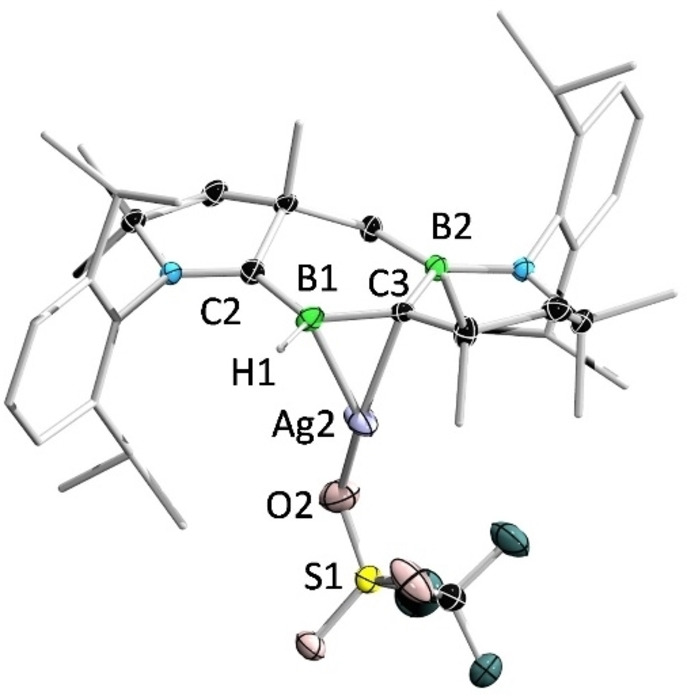
Solid‐state structure of **11**. Ellipsoids are shown at the 50 % probability level. Ellipsoids of peripheral groups and all hydrogen atoms except that bound to B1 have been removed for clarity.

**Figure 14:**
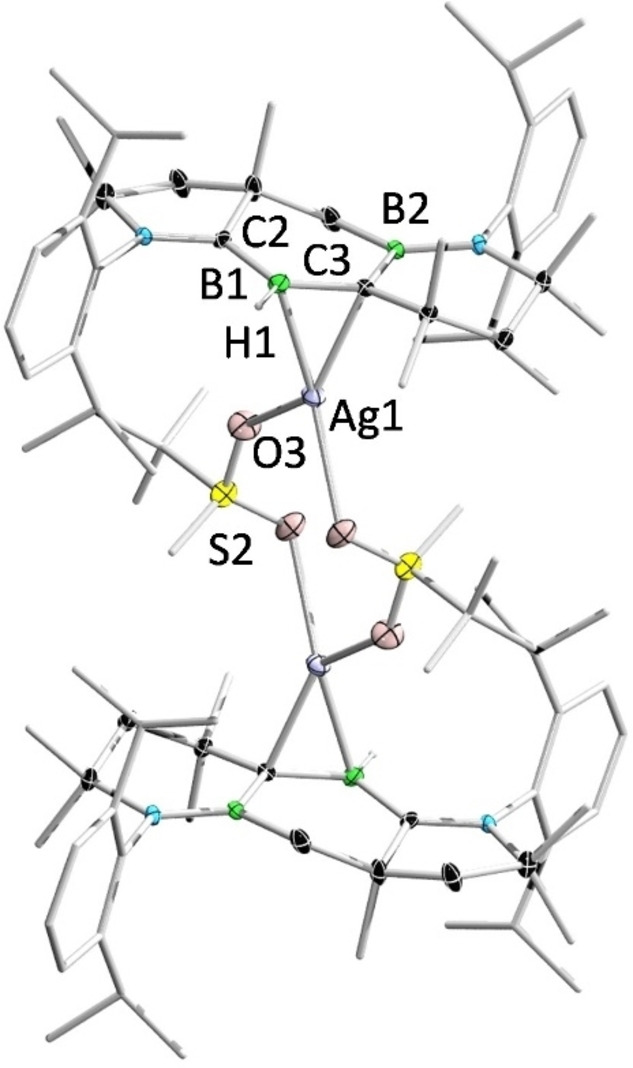
Solid‐state structure of **11′**. Ellipsoids are shown at the 50 % probability level. Ellipsoids of peripheral groups and all hydrogen atoms except that bound to B1 have been removed for clarity.

The structures of **11** and **11′** were determined by single‐crystal X‐ray diffraction analysis (Figure 13 and Figure 14). In the solid‐state structure of **11**, the cationic Ag^I^ center is coordinated by the B=C double bond and the counterion (OTf^−^) in an η^2^ and η^1^ fashion, respectively. The B1 and C3 atoms adopt trigonal planar geometries (sum of bond angles: ∑B1=359.6°, ∑C3=359.1°). The B1−Ag2 distance (2.364(4) Å) is slightly longer than those observed in diborene‐ and 1,2‐diboraallene‐Ag^I^ complexes (2.276–2.366 Å).[^16^, ^39^] The C3−Ag2 distance (2.263(3) Å) is comparable to that reported in an Ag^I^–ethylene complex,^[40]^ but considerably shorter than that in an Ag^I^–borataalkene adduct (2.633(3) Å).^[41]^ In contrast to **11**, **11′** is dinuclear, featuring an Ag_2_O_4_S_2_ eight‐membered ring. The B1−Ag1 distance (2.446(3) Å) in **11′** is slightly longer than that in **11**. It is noteworthy that **11** and **11′** represent the first examples of alkylideneborane–Ag^I^ complexes.

## Conclusion

This report demonstrates the in situ, one‐pot synthesis of the first doubly CAAC‐supported unsymmetrical, cyclic diborene. The presence of the strong σ‐donating/π‐accepting CAAC ligands as well as an unsymmetrical coordination environment renders a small H–L gap in this diborene. The new diborene reacts with a range of unsaturated molecules, a dihydroborane, P_4_ and formed stable π complexes with Au^I^ fragments. The new diborene was also found to undergo thermal rearrangement to give a cyclic alkylideneborane, which binds in a π fashion to Ag^I^.

## Conflict of interest

The authors declare no conflict of interest.

## Supporting information

As a service to our authors and readers, this journal provides supporting information supplied by the authors. Such materials are peer reviewed and may be re‐organized for online delivery, but are not copy‐edited or typeset. Technical support issues arising from supporting information (other than missing files) should be addressed to the authors.

Supporting Information

Supporting Information
