# Supplementary material for: An Unsymmetrical, Cyclic Diborene Based on a Chelating CAAC Ligand and its Small‐Molecule Activation and Rearrangement Chemistry
Source: Angew Chem Int Ed Engl. 2021 Dec 3;61(3):e202113947. doi: 10.1002/anie.202113947 (PMC9299934; doi:10.1002/anie.202113947)
Supplement: Supplementary file 2 — Supporting Information [file ANIE-61-0-s001.pdf]

Angewandte Chemie
Eine Zeitschrift der Gesellschaft Deutscher Chemiker

Supporting Information

An Unsymmetrical, Cyclic Diborene Based on a Chelating CAAC Ligand and its Small-Molecule Activation and Rearrangement Chemistry

*Wei Lu, Arumugam Jayaraman, Felipe Fantuzzi, Rian D. Dewhurst, Marcel Härterich, Maximilian Dietz, Stephan Hagspiel, Ivo Krummenacher, Kai Hammond, Jingjing Cui, and Holger Braunschweig**

anie_202113947_sm_miscellaneous_information.pdf
anie_202113947_sm_cif.zip

1. Synthesis, physical and spectroscopic data for all new compounds

General considerations:
All reactions were performed under an atmosphere of dry argon by using standard Schlenk or glovebox techniques; solvents were dried over Na metal, K metal or CaH_2. ^{1}H, ^{11}B, ^{13}C and ^{31}P spectra were obtained with a Bruker Avance 400 at 298 K unless otherwise stated. NMR multiplicities are abbreviated as follows: s = singlet, d = doublet, t = triplet, sep = septet, m = multiplet, br = broad signal. Coupling constants *J* are given in Hz. HRMS spectra were obtained from a Thermo Scientific Exactive Plus spectrometer. UV-Vis spectra were measured with a METTLER TOLEDO UV-vis-Excellence UV5 spectrophotometer. Fourier-transform infrared (FT-IR) spectra were recorded on a Bruker ALPHA-Transmittance FT-IR Spectrometer. For quantitative NMR spectra, 1,3,5-trimethoxylbenzene was used as internal standard to monitor yields, unless otherwise stated.
Compounds **VI**[1] and **V**[2] were synthesized according to literature methods.

Synthesis of compound 1:

At ambient temperature, a benzene solution (5 mL) of $B_2Br_4{\cdot}(SMe_2)_2$ (290 mg, 0.63 mmol) was added dropwise to a CAAC (344 mg, 1.28 mmol) solution under vigorous stirring. The mixture was stirred for 2 h to give a white suspension, then two equivalents of KC_8 (172 mg, 1.27 mmol) was added in small portions. The resulting suspension was allowed to stir overnight to give a blue mixture, then filtered and the residue was washed with a small amount of benzene (2 mL). The filtrate was combined and dried under vacuum, then washed with pentane and dried to afford an orange solid (54%). Single crystals of **1** were obtained by slow evaporation of a benzene solution in a glovebox.

^{1}H NMR (400 MHz, benzene-*d*$_6$) δ 7.26 – 7.17 (m, 4H, Ar*H*), 7.13 – 7.00 (m, 8H, Ar*H*), 4.54 (sep, *J* = 6.7 Hz, 1H, C*H*(CH$_3$)$_2$), 4.27 (sep, *J* = 6.7 Hz, 1H, C*H*(CH$_3$)$_2$), 3.44 (sep, *J* = 6.7 Hz, 1H, C*H*(CH$_3$)$_2$), 3.35 (sep, *J* = 6.7 Hz, 2H, C*H*(CH$_3$)$_2$), 3.02 (sep, *J* = 6.7 Hz, 1H, C*H*(CH$_3$)$_2$), 2.95 (sep, *J* = 6.7 Hz, 1H, C*H*(CH$_3$)$_2$), 2.79 (sep, *J* = 6.7 Hz, 1H, C*H*(CH$_3$)$_2$), 2.61 (d, *J* = 12.0 Hz, 1H, C*H*$_2$), 2.29 (d, *J* = 12.0 Hz, 1H, C*H*$_2$), 2.24 (s, 3H, C*H*$_3$), 2.04 (s, 3H, C*H*$_3$), 2.01 – 1.95 (m, 9H, C*H*$_3$), 1.89 (s, 3H, C*H*$_3$), 1.83 (d, *J* = 6.7 Hz, 3H, C*H*$_3$), 1.75 (d, *J* = 6.7 Hz, 3H, C*H*$_3$), 1.71 (d, *J* = 6.7 Hz, 3H, C*H*$_3$), 1.66 (d, *J* = 6.7 Hz, 1H, C*H*$_2$), 1.64 – 1.59 (m, 5H, C*H*$_2$ & C*H*$_3$), 1.57 (d, *J* = 6.7 Hz, 6H, C*H*$_3$), 1.53 (s, 2H, C*H*$_2$), 1.52 – 1.47 (m, 4H, C*H*$_2$ & C*H*$_3$), 1.46 (s, 3H, C*H*$_3$), 1.35 – 1.33 (m, 6H, C*H*$_3$), 1.27 (s, 3H, C*H*$_3$), 1.23 (s, 3H, C*H*$_3$), 1.20 – 1.16 (m, 6H, C*H*$_3$), 1.15 (d, *J* = 6.7 Hz, 3H, C*H*$_3$), 1.13 – 1.11 (m, 6H, C*H*$_3$), 1.09 – 1.08 (m, 6H, C*H*$_3$), 1.05 – 1.03 (m, 3H, C*H*$_2$), 0.92 (s, 3H, C*H*$_3$), 0.87 (s, 9H, C*H*$_3$), 0.83 (s, 3H, C*H*$_3$), 0.56 – 0.52 (d, *J* = 12 Hz, 1H, C*H*$_2$). **^{11}B NMR (128 MHz, benzene-*d*$_6$)** δ 2.03 (br, s), −10.46 (br, s). **^{13}C{^{1}H} NMR (101 MHz, benzene-*d*$_6$)** δ 147.9 (*C*-Ar), 147.5 (*C*-Ar), 147.1 (*C*-Ar), 147.0 (*C*-Ar), 146.7 (*C*-Ar), 146.7 (*C*-Ar), 146.6 (*C*-Ar), 146.3 (*C*-Ar), 135.8 (*C*-Ar), 135.1 (*C*-Ar), 133.6 (*C*-Ar), 133.4 (*C*-Ar), 129.6 (*C*H-Ar), 129.5 (*C*H-Ar), 129.3 (*C*H-Ar), 129.2 (*C*H-Ar), 125.9 (*C*H-Ar), 125.7 (*C*H-Ar), 125.5 (*C*H-Ar), 125.4 (*C*H-Ar), 125.1 (*C*H-Ar), 124.6 (*C*H-Ar), 124.5 (*C*H-Ar), 123.9 (*C*H-Ar), 79.6 (*C*-Alkyl), 79.4 (*C*-Alkyl), 76.9 (*C*-Alkyl), 76.8 (*C*-Alkyl), 58.1 (*C*H$_3$), 55.7 (*C*H$_3$), 54.9 (*C*H$_3$), 54.8 (*C*H$_3$), 54.1 (*C*H$_2$), 53.3 (*C*H$_2$), 52.7 (*C*H$_2$), 52.5 (*C*H$_2$), 36.2 (br, *C*H$_2$), 35.0 (*C*H$_3$), 34.3 (*C*H$_3$), 34.2 (br, *C*H$_2$), 34.0 (*C*H$_3$), 32.6 (*C*H$_3$), 32.6 (*C*H$_3$), 31.0 (*C*H$_3$), 30.9 (*C*H$_3$), 30.6 (*C*H$_3$), 29.6 (*C*H$_3$), 29.6 (*C*H-Alkyl), 29.4 (*C*H$_3$), 29.2 (*C*H-Alkyl), 28.9 (*C*H-Alkyl), 28.9 (*C*H-Alkyl), 28.8 (*C*H-Alkyl), 28.6 (*C*H-Alkyl), 28.6 (*C*H$_3$), 28.4 (*C*H$_3$), 28.4 (*C*H$_3$), 28.4 (*C*H-Alkyl), 28.4 (*C*H$_3$), 28.1 (*C*H-Alkyl), 28.1 (*C*H$_3$), 27.4

(CH_3), 26.7 (CH_3), 26.6 (CH_3), 26.4 (CH_3), 26.4 (CH_3), 26.3 (CH_3), 26.2 (CH_3), 25.8 (CH_3), 25.4 (CH_3), 25.3 (CH_3), 25.2 (CH_3), 24.8 (CH_3), 24.1 (CH_3), 23.6 (CH_3), 23.4 (CH_3). **LIFDI-MS:** *m/z* calcd. For $[C_{40}H_{63}B_2Br_2N_2]^+$: 753.3518; found: 753.3499.

Alternative synthesis of compound 1 (compropotionation):

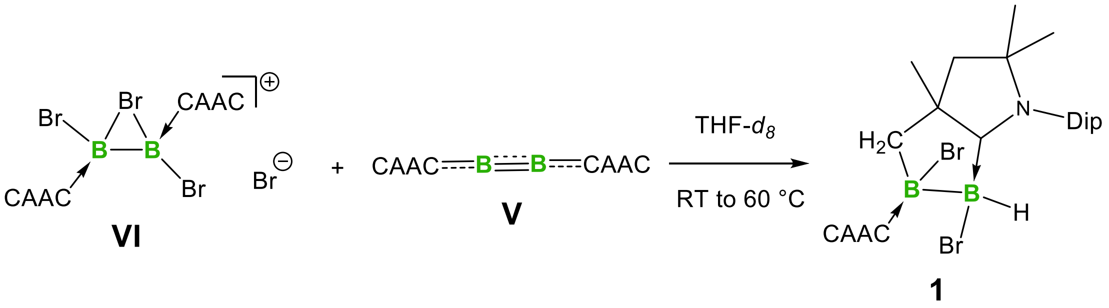

The reaction of **VI** and **V** was first examined in THF-*d*8 at ambient temperature. However, only a small amount (~10%) of **1** was observed by NMR spectroscopy after three days (Figure S5-S6).

When a THF-*d*8 solution of **VI** (20 mg, 0.022 mmol) and **V** (15.4 mg, 0.026 mmol) mixture was allowed to heat at 60 °C overnight, a green mixture was formed. NMR spectroscopy revealed a full conversion of the starting materials to **1** (85% NMR yield, based on **VI**) as well as some unidentified species (Figure S7-S8).

Synthesis of compound 2:

A benzene solution (10 mL) of $B_2Br_4{\cdot}(SMe_2)_2$ (1.0 g, 2.17 mmol) was slowly added to a CAAC (1.16 g, 4.33 mmol) solution at ambient temptation. The mixture was stirred for 2 h to give a white suspension, then KC_8 (0.585 g, 4.33 mmol) was slowly added in small portions. The reaction mixture was allowed to stir overnight, followed by treatment with two equivalents of KC_8 (0.585 g, 4.33 mmol) to afford a dark blue mixture. After the reaction was complete, the suspension was filtered, and the residue was washed with benzene (2 x 5 mL). The combined filtrate was dried under vacuum and washed with a small amount of pentane to afford compound **2** as a dark blue crystalline solid (0.7 g, 54%).

^{1}H NMR (400 MHz, benzene-*d*6) δ 7.24 – 7.15 (m, 2H, Ar*H*), 7.13 – 7.05 (m, 4H, Ar*H*), 5.25 (br, s, 1H, B*H*), 3.33 (sep, *J* = 6.7 Hz, 1H, C*H*(CH_3)$_2$), 3.13 (sep, *J* = 6.7 Hz, 1H, C*H*(CH_3)$_2$), 3.02 (sep, *J* = 6.7 Hz, 1H, C*H*(CH_3)$_2$), 2.90 (sep, *J* = 6.7 Hz, 1H, C*H*(CH_3)$_2$), 2.04 (s, 3H, CH_3), 1.99 – 1.92 (m, 2H, CH_2), 1.91 (s, 3H, CH_3), 1.78 (q, *J* = 12.6 Hz, 2H, CH_2), 1.71 – 1.66 (m, 3H, CH_3), 1.51 (d, *J* = 6.7 Hz, 3H, CH_3), 1.40 (d, *J* = 6.7 Hz, 3H, CH_3), 1.32 (d, *J* = 6.7 Hz, 3H, CH_3), 1.28 (d, *J* = 6.7 Hz, 3H, CH_3), 1.23 (d, *J* = 6.7 Hz, 3H, CH_3), 1.21 – 1.15 (m, 12H, CH_3), 1.12 (s, 3H, CH_3), 1.10 (s, 3H, CH_3), 0.99 (s, 3H, CH_3), 0.94 – 0.88 (m, 1H, CH_2), 0.86 – 0.76 (m, 1H, CH_2). **^{11}B NMR (128 MHz, benzene-*d*6)** δ 48.37 (br, s), 43.20 (br, s). **^{13}C{^{1}H} NMR (101 MHz, benzene-*d*6)** δ 148.4 (*C*-Ar), 148.3 (*C*-Ar), 147.9 (*C*-Ar), 147.3 (*C*-Ar), 137.3 (*C*-Ar), 135.2 (*C*-Ar), 128.6 (*C*H-Ar), 128.2 (*C*H-Ar), 124.5 (*C*H-Ar), 124.3 (*C*H-Ar), 124.3 (*C*H-Ar), 123.8 (*C*H-Ar), 76.0 (*C*-Alkyl), 70.0 (*C*-Alkyl), 58.0 (*C*-Alkyl), 55.4 (CH_2), 53.7 (CH_2), 50.5 (*C*-Alkyl), 38.6 (CH_2), 35.7 (CH_3), 35.3 (CH_3), 34.8 (CH_3), 30.1 (CH_3), 29.3

(CH_3), 29.1 (CH_3), 28.8 ($CH(CH_3)_2$), 28.7 ($CH(CH_3)_2$), 28.7 ($CH(CH_3)_2$), 27.9 ($CH(CH_3)_2$), 27.2 (CH_3), 26.4 (CH_3), 25.6 (CH_3), 25.2 (CH_3), 25.1 (CH_3), 25.0 (CH_3), 23.2 (CH_3), 22.5 (CH_3). **FT-IR** (solid, cm^{-1}): 2965, 2918, 2851, 2426 (BH), 1476, 1441, 1417, 1333, 1279, 1203, 1179, 1123, 1082, 1042, 901, 800. **LIFDI-MS:** *m/z* calcd. For $[C_{40}H_{63}B_2N_2]^+$: 593.5172; found: 593.5161.

Synthesis of compound 2 from 1:

KC_8 (7 mg, 0.052 mmol) was added to a sealable NMR tube containing a benzene-*d*$_6$ solution of **1** (17.8 mg, 0.024 mmol). The reaction was monitored by NMR spectroscopy. After two days a blue mixture was afforded, and ^{1}H and ^{11}B NMR spectra indicated that full conversion of **1** to **2** was achieved (95 % NMR yield) (Figure S13-S14).

Synthesis of compounds 3 and 4:

[AuCl(PCy$_3$)] (30 mg, 0.058 mmol) was slowly added to a sealable NMR tube containing a benzene-*d*$_6$ solution of **2** (30 mg, 0.051 mmol). The color of the mixture turned from green via brown to red-brown overnight, and NMR spectroscopy revealed nearly quantitative conversion of **2** to the diborene-Au(I) complexes **3** (63%) and **4** (22%). After the reaction, all volatiles were removed under reduced pressure, the residue was extracted with pentane and concentrated to give an orange red powder of **3**. Single crystals of **3** were obtained by slow evaporation of a benzene solution.

Alternative synthesis of 4:

Benzene-*d*$_6$ (1 mL) was added to a sealable NMR tube containing a mixture of **2** (33 mg, 0.056 mmol) and [AuCl(PCy$_3$)] (11 mg, 0.022 mmol). The reaction mixture was allowed to stand overnight, during which time red crystals were formed on the wall of the NMR tube. The supernatant of the mixture was carefully removed with a pipette, the residue was washed with a small amount of benzene and dried under vacuum, from which compound **4** was obtained as a red solid (17 mg, 70%).

Complex 3:
^{1}H NMR (400 MHz, DCM-*d*$_2$) δ 7.56 (t, *J* = 7.8 Hz, 1H, Ar*H*), 7.49 – 7.37 (m, 2H, Ar*H*), 7.37 – 7.25 (m, 3H, Ar*H*), 4.14 (br, s, 1H, B*H*), 2.96 (sep, *J* = 6.7 Hz, 1H, C*H*(CH$_3$)$_2$), 2.79 (sep, *J* = 6.7 Hz, 2H, C*H*(CH$_3$)$_2$), 2.53 (sep, *J* = 6.7 Hz, 1H, C*H*(CH$_3$)$_2$), 2.31 – 2.21 (m, 4H, C*H*$_2$ & C*H*$_3$), 2.21 – 2.06 (m, 5H, C*H* & C*H*$_2$), 1.99 – 1.85 (m, 15H, C*H*$_2$ & C*H*$_3$), 1.84 – 1.72 (m, 7H, C*H*$_2$ & C*H*$_3$), 1.53 (d, *J* = 6.6 Hz, 3H, C*H*$_3$), 1.46 (d, *J* = 3.4 Hz, 9H, C*H*$_2$ & C*H*$_3$), 1.42 – 1.22 (m, 28H, C*H*$_2$ & C*H*$_3$), 1.19 (d, *J* = 6.7 Hz, 3H, C*H*$_3$), 1.17 (s, 3H, C*H*), 1.13 (d, *J* = 6.8 Hz, 3H, C*H*$_3$), 1.08 (d, *J* = 6.6 Hz, 3H, C*H*$_3$), 0.61 (dd, *J* = 16.3, 3.2 Hz, 1H, C*H*$_2$). **^{11}B NMR (128 MHz, DCM-*d*$_2$)** δ 38.13 (br, s), 30.90 (br, s). **^{31}P NMR (162 MHz, DCM-*d*$_2$)** δ 64.70 (s). **^{13}C{^{1}H} NMR (101 MHz, DCM-*d*$_2$)** δ 147.4 (*C*-Ar), 146.4 (*C*-Ar), 146.1 (*C*-Ar), 145.9 (*C*-Ar), 134.4 (*C*-Ar), 133.1 (*C*-Ar), 130.7 (*C*H-Ar), 129.5 (*C*H-Ar), 125.9 (*C*H-Ar),

125.5 (*C*H-Ar), 124.9 (*C*H-Ar), 124.7 (*C*H-Ar), 78.9 (*C*-Alkyl), 76.3 (*C*-Alkyl), 53.8 (CH_2), 53.0 (CH_2), 37.3 (br, CH_2), 35.9 (CH_3), 35.9 (CH_3), 34.4 (*C*H-Alkyl), 34.1 (*C*H-Alkyl), 33.3 (CH_3), 32.6 (CH_3), 31.1 (CH_3), 30.8 (CH_2), 30.7 (CH_2), 30.2 (CH_3), 29.5 (*C*H-Alkyl), 29.4 (*C*H-Alkyl), 28.8 (*C*H-Alkyl), 28.2 (CH_3), 27.9 (CH_3), 27.5 (CH_2), 27.4 (CH_2), 27.2 (CH_3), 26.9 (CH_3), 26.7 (CH_3), 26.2 (CH_2), 26.1 (CH_2), 25.9 (CH_3), 25.8 (CH_3), 24.7 (CH_3), 22.9 (CH_3), 22.8 (CH_3). **LIFDI-MS:** *m/z* calcd. For [$C_{58}H_{95}AuB_2N_2P$]: 1069.7079; found: 1069.7066.

Complex 4:

^{1}H NMR (400 MHz, DCM-*d*$_2$) δ 7.47 (t, *J* = 7.7 Hz, 1H, Ar*H*), 7.42 – 7.38 (m, 1H, Ar*H*), 7.27 (t, *J* = 7.4 Hz, 4H, Ar*H*), 2.99 (sep, *J* = 6.7 Hz, 1H, C*H*(CH_3)$_2$), 2.92 – 2.84 (sep, *J* = 6.7 Hz, 1H, C*H*(CH_3)$_2$), 2.76 (sep, *J* = 6.7 Hz, 2H, C*H*(CH_3)$_2$), 2.36 – 2.24 (m, 1H, C*H*$_2$), 2.12 (s, 2H, C*H*$_2$), 2.01 (s, 3H, C*H*$_3$), 1.96 – 1.88 (m, 1H, C*H*$_2$), 1.79 (s, 3H, C*H*$_3$), 1.69 (s, 3H, C*H*$_3$), 1.43 (d, *J* = 10.5 Hz, 7H, C*H*$_2$ & C*H*$_3$), 1.40 – 1.24 (m, 22H, C*H*$_2$ & C*H*$_3$), 1.21 (s, 3H, C*H*$_3$), 1.16 (d, *J* = 6.6 Hz, 3H, C*H*$_3$), 1.11 (d, *J* = 6.6 Hz, 3H, C*H*$_3$). **^{11}B NMR (128 MHz, DCM-*d*$_2$)** δ 33.80 (br, s), 16.00 (br, s). **^{13}C{^{1}H} NMR (101 MHz, DCM-*d*$_2$)** δ 147.6 (*C*-Ar), 147.2 (*C*-Ar), 146.8 (*C*-Ar), 146.2 (*C*-Ar), 134.6 (*C*-Ar), 133.7 (*C*-Ar), 129.8 (*C*H-Ar), 129.0 (*C*H-Ar), 125.6 (*C*H-Ar), 125.2 (*C*H-Ar), 124.6 (*C*H-Ar), 124.4 (*C*H-Ar), 77.8 (*C*-Alkyl), 75.3 (*C*-Alkyl), 55.1 (*C*-Alkyl), 54.9 (CH_2), 53.6 (CH_2), 35.3 (CH_3), 30.7 (CH_3), 30.4 (CH_3), 29.5 (CH_3), 29.2 (CH_3), 29.1 (C*H*(CH_3)$_2$), 28.8 (C*H*(CH_3)$_2$), 28.4 (C*H*(CH_3)$_2$), 27.8 (CH_3), 26.7 (CH_3), 26.0 (CH_3), 25.8 (CH_3), 24.5 (CH_3), 23.1 (CH_3), 22.6 (CH_3). **LIFDI-MS:** *m/z* calcd. for [$C_{40}H_{62}AuB_2N_2Cl$]: 824.4448; found: 824.4429. *m/z* calcd. for [$C_{40}H_{62}AuB_2N_2Br$]: 868.3943; found: 868.3929.

Synthesis of compound 6:

At ambient temperature, benzene was slowly added to a mixture of **2** (40 mg, 0.068 mmol) and $DurBH_2$ (10 mg, 0.068) under vigorous stirring. The color of the reaction mixture turned from blue via purple to red within 15 min. The mixture was allowed to stir for 2 h, then filtered and concentrated. Slow evaporation of the benzene solution gave compound **6** as a red crystalline solid (30%).

^{1}H NMR (400 MHz, benzene-*d*$_6$) δ 7.36 – 7.24 (m, 2H, Ar*H*), 7.21 – 7.17 (m, 1H, Ar*H*), 7.13 – 6.91 (m, 4H, Ar*H*), 4.00 (sep, *J* = 6.7 Hz, 1H, C*H*(CH_3)$_2$), 3.42 (sep, *J* = 6.7 Hz, 1H, C*H*(CH_3)$_2$), 3.13 (s, 3H, DurC*H*$_3$), 2.98 – 2.70 (m, 4H, C*H*(CH_3)$_2$ & DurC*H*$_3$), 2.41 (s, 6H, DurC*H*$_3$), 2.28 (sep, *J* = 6.7 Hz, 1H, C*H*(CH_3)$_2$), 1.85 (q, *J* = 12.0 Hz, 2H, C*H*$_2$), 1.75 – 1.66 (m, 6H, C*H*$_3$), 1.61 – 1.53 (m, 1H, C*H*$_2$), 1.42 (d, *J* = 6.7 Hz, 3H, C*H*$_3$), 1.40 (s, 3H, C*H*$_3$), 1.38 – 1.34 (m, 1H, C*H*$_2$), 1.34 – 1.29 (m, 4H, C*H*$_2$ & C*H*$_3$), 1.21 (d, *J* = 6.9 Hz, 3H, C*H*$_3$), 1.17 – 1.12 (m, 4H, C*H*$_2$ & C*H*$_3$), 1.10 (s, 3H, C*H*$_3$), 1.08 – 1.05 (m, 6H, C*H*$_3$), 1.02 – 0.94 (m, 6H, C*H*$_3$), 0.76 – 0.61 (m, 9H, C*H*$_3$). **^{11}B NMR (128 MHz, benzene-*d*$_6$)** δ 7.26, −5.41, −24.32. **^{13}C{^{1}H} NMR (101 MHz, benzene-*d*$_6$)** δ 149.4 (*C*-Ar), 148.8 (*C*-Ar), 145.5 (*C*-Ar), 145.2 (*C*-Ar), 137.5 (*C*-Ar), 133.7 (*C*-Ar), 132.4 (*C*-Ar), 129.3 (*C*H-Ar), 128.6 (*C*H-Ar), 127.4 (*C*H-Ar), 125.9 (*C*H-Ar), 125.0 (*C*H-Ar), 124.9 (*C*H-Ar), 124.3 (*C*H-Ar), 75.7 (*C*-Alkyl), 72.0 (*C*-Alkyl), 55.6 (*C*-Alkyl), 55.1 (CH_2), 53.1 (CH_2), 52.0 (*C*-Alkyl), 38.3 (CH_2), 33.8 (CH_3), 30.6 (CH_3), 29.7 (CH_3), 29.7 (CH_3), 29.3 (CH_3), 29.1 (C*H*(CH_3)$_2$), 28.7 (C*H*(CH_3)$_2$), 28.1 (C*H*(CH_3)$_2$), 27.7 (C*H*(CH_3)$_2$), 27.3 (CH_3), 27.1 (CH_3), 26.5 (CH_3), 26.2 (CH_3), 26.2 (CH_3), 26.0 (CH_3), 25.7 (CH_3), 25.3 (CH_3), 24.1 (CH_3), 23.2 (CH_3), 21.5 (CH_3). **LIFDI-MS:** *m/z* calcd. For [$C_{50}H_{77}B_3N_2$]: 738.6360; found: 738.6342.

Synthesis of compound 7:

At ambient temperature, white phosphorous (P_4) (6 mg, 0.038 mmol) was added to a sealable NMR tube containing a benzene-d_6 solution of **2** (30 mg, 0.051 mmol). After two days, a red mixture was obtained, and NMR spectroscopy revealed quantitative formation of a new species. Slowly evaporation of the reaction mixture in a glovebox afforded **7** as red crystals (24 mg, 71%).

^{1}H NMR (400 MHz, benzene-d_6) δ 7.22 (m, 1H, Ar*H*), 7.15 – 7.07 (m, 4H, Ar*H*), 7.07 – 7.02 (m, 1H, Ar*H*), 3.11 – 2.91 (m, 4H, C*H*(CH_3)$_2$), 1.89 (d, *J* = 12.4 Hz, 1H, C*H*$_2$), 1.82 (d, *J* = 6.7 Hz, 6H, C*H*$_3$), 1.75 (s, 3H, C*H*$_3$), 1.73 – 1.66 (m, 4H, C*H*$_2$ & C*H*$_3$), 1.62 (d, *J* = 6.7 Hz, 3H, C*H*$_3$), 1.59 – 1.55 (m, 5H, C*H*$_2$), 1.46 – 1.39 (m, 1H, C*H*$_2$), 1.31 (d, *J* = 6.7 Hz, 3H, C*H*$_3$), 1.20 – 1.15 (m, 10H, C*H*$_2$ & C*H*$_3$), 1.13 (d, *J* = 6.7 Hz, 3H, C*H*$_3$), 1.04 (s, 3H, C*H*$_3$), 1.02 (s, 3H, C*H*$_3$), 0.95 (s, 3H, C*H*$_3$), 0.94 (s, 3H, C*H*$_3$). **^{11}B NMR (128 MHz, benzene-d_6)** δ −12.87, −21.49 (d, *J* = 123.1 Hz). **^{31}P NMR (162 MHz, benzene-d_6)** δ −118.84 (br), −216.72 (br). **^{13}C{^{1}H} NMR (101 MHz, benzene-d_6)** δ 147.8 (*C*-Ar), 147.1 (*C*-Ar), 146.4 (*C*-Ar), 145.9 (*C*-Ar), 137.0 (*C*-Ar), 134.2 (*C*-Ar), 129.1 (*C*H-Ar), 128.5 (*C*H-Ar), 125.2 (*C*H-Ar), 124.9 (*C*H-Ar), 124.6 (*C*H-Ar), 124.2 (*C*H-Ar), 72.8 (*C*-Alkyl), 70.2 (*C*-Alkyl), 56.7 (*C*H$_2$), 55.6 (*C*H$_2$), 52.3 (*C*-Alkyl), 49.2 (*C*-Alkyl), 38.3 – 37.7 (m, *C*H$_3$), 32.6 – 32.1 (m, *C*H$_3$), 31.9 – 31.3 (m, *C*H$_2$), 30.3 (*C*H$_3$), 29.3 – 29.2 (m, *C*H$_3$), 29.1 (*C*H(CH_3)$_2$), 28.9 (*C*H(CH_3)$_2$), 28.8 (*C*H(CH_3)$_2$), 28.7 (*C*H(CH_3)$_2$), 28.3 (*C*H$_3$), 28.3 (*C*H$_3$), 28.2 (*C*H$_3$), 26.2 (*C*H$_3$), 25.9 (*C*H$_3$), 25.8 – 25.7 (m, *C*H$_3$), 25.6 (*C*H$_3$), 25.6 (*C*H$_3$), 25.5 (*C*H$_3$), 25.5 (*C*H$_3$), 23.5 (*C*H$_3$), 23.2 (*C*H$_3$). **LIFDI-MS:** *m/z* calcd. For [$C_{40}H_{62}B_2N_2P_2$]: 654.4569; found: 654.4553.

Synthesis of compound 8:

(4-methylphenyl)acetylene (TolCCH) (16 mg, 0.140 mmol) was added to a benzene solution of **2** (40 mg, 0.068 mmol) at ambient temperature, and the blue mixture was stirred for 2 d to give a white suspension. After the reaction, all volatiles were removed under reduced pressure and the residue was washed with pentane and dried over vacuum to give **8** as a white solid (34 mg, 61%). Single crystals of **8** were obtained by slow evaporation of a benzene solution at ambient temperature.

^{1}H NMR (400 MHz, benzene-d_6) δ 7.39 – 7.21 (m, 4H, Ar*H*), 7.10 (d, *J* = 4.6 Hz, 2H, Ar*H*), 6.99 – 6.88 (m, 3H, Ar*H*), 6.82 – 6.60 (m, 5H, Ar*H*), 4.50 – 4.24 (sep, *J* = 6.7 Hz, 1H, C*H*(CH_3)$_2$), 3.69 (s, 1H, C_{CAAC}=C*H*), 3.03 (sep, *J* = 6.7 Hz, 2H, C*H*(CH_3)$_2$), 2.91 (d, *J* = 16 Hz, 1H, C*H*$_2$), 2.61 (sep, *J* = 6.7 Hz, 1H, C*H*(CH_3)$_2$), 2.29 – 2.21 (m, 2H, C*H* & C*H*$_2$), 2.17 (s, 3H, C*H*$_3$), 2.09 – 1.94 (m, 2H, C*H*$_2$), 1.93 (s, 3H, C*H*$_3$), 1.86 – 1.79 (m, 4H, C*H*$_2$ & C*H*$_3$), 1.75 (s, 6H, C*H*$_3$), 1.64 (d, *J* = 6.7 Hz, 3H, C*H*$_3$), 1.58 (s, 3H, C*H*$_3$), 1.51 (d, *J* = 6.8 Hz, 3H, C*H*$_3$), 1.35 – 1.26 (m, 4H, C*H*$_2$ & C*H*$_3$), 1.18 (d, *J* = 6.7 Hz, 3H, C*H*$_3$), 1.13 (d, *J* = 6.6 Hz, 3H, C*H*$_3$), 1.06 (s, 3H, C*H*$_3$), 1.02 (s, 3H, C*H*$_3$), 0.96 (d, *J* = 6.6 Hz, 3H, C*H*$_3$), 0.82 (s, 3H, C*H*$_3$), 0.61 (d, *J* = 6.7 Hz, 3H, C*H*$_3$), 0.47 (d, *J* = 6.7 Hz, 3H, C*H*$_3$). **^{11}B NMR (128 MHz, chloroform-*d*)** δ 76.74 (br), −15.29. **^{13}C{^{1}H} NMR (101 MHz, benzene-d_6)** δ 191.9 (C_{CAAC}), 150.0 (*C*-Ar), 149.9 (*C*-Ar), 148.4 (*C*-Ar), 147.6 (*C*-Ar), 145.5 (*C*-Ar), 143.9 (*C*-Ar), 135.9 (*C*-Ar), 133.8 (*C*-Ar), 133.3 (*C*H-Ar), 131.5 (*C*H-Ar), 129.5 (*C*H-Ar), 128.7 (*C*H-Ar), 128.5 (*C*H-Ar), 125.9 (*C*H-Ar), 125.8 (*C*H-Ar), 125.0 (*C*H-Ar), 124.7 (*C*H-Ar), 124.0 (*C*H-Ar), 123.4 (*C*H-Ar), 103.0 (C≡*C*-Ar), 72.6 (C_{CAAC}=*C*H), 68.6 (*C*-Alkyl), 64.4 (*C*-Alkyl), 60.2 (*C*H$_2$), 57.9 (*C*H$_2$), 55.5 (C_{CAAC}*H*), 45.2 (*C*H$_2$), 44.0 (*C*H$_3$), 43.1 (*C*H$_3$), 33.7 (*C*H$_3$), 33.2 (*C*H$_3$), 32.3 (*C*H$_3$), 30.4 (*C*H$_3$), 29.9 (*C*H$_3$), 28.8 (*C*H(CH_3)$_2$), 28.6

(*C*H(CH_3)$_2$), 28.5 (*C*H(CH_3)$_2$), 28.2 (*C*H_3), 27.7 (*C*H_3), 27.0 (*C*H_3), 26.97 (*C*H_3), 26.93 (*C*H_3), 26.8 (*C*H(CH_3)$_2$), 25.9 (*C*H_3), 25.7 (*C*H_3), 23.7 (*C*H_3), 23.58 (*C*H_3), 23.52 (*C*H_3), 21.0 (*C*H_3), 20.9 (*C*H_3). **LIFDI-MS:** *m/z* calcd. For [$C_{58}H_{78}B_2N_2$]: 824.6346; found: 824.6331.

Synthesis of compound 9:

A benzene solution of **2** (30 mg, 0.051 mmol) and Hg(CCPh)$_2$ (10 mg, 0.025 mmol) was stirred overnight to give a red brown mixture. After the reaction, the mixture was filtered, and slow evaporation of the benzene mixture gave **9** as orange crystals. However, complex **9** was isolated only in small amounts and could not be characterized by NMR spectroscopy.

Synthesis of compound 10:

A benzene-*d$_6$* solution of **2** (42 mg, 0.071 mmol) was heated at 80 °C overnight to give an orange mixture, in which a quantitative conversion of **2** to **10** was manifested by NMR spectroscopy (>95% NMR yield). (*No further purification of **10** was performed for further reactivity studies*). Orange single crystals of **10** were obtained by slow evaporation of a pentane solution at ambient temperature in a glovebox.

^{1}H NMR (400 MHz, benzene-*d$_6$*) δ 7.24 – 7.20 (m, 2H, Ar*H*), 7.16 – 7.11 (m, 2H, Ar*H*), 7.08 – 7.01 (m, 2H, Ar*H*), 4.53 (br, s, 1H, B*H*), 3.78 (sep, *J* = 6.7 Hz, 1H, C*H*(CH_3)$_2$), 3.52 (sep, *J* = 6.7 Hz, 1H, C*H*(CH_3)$_2$), 2.94 (sep, *J* = 6.7 Hz, 1H, C*H*(CH_3)$_2$), 2.70 (sep, *J* = 6.7 Hz, 1H, C*H*(CH_3)$_2$), 2.03 – 1.94 (m, 2H, C*H$_2$*), 1.71 – 1.67 (m, 2H, C*H$_2$*), 1.66 (s, 3H, C*H$_3$*), 1.63 (s, 3H, C*H$_3$*), 1.58 (s, 3H, C*H$_3$*), 1.39 (d, *J* = 6.7 Hz, 3H, C*H$_3$*), 1.38 (d, *J* = 6.7 Hz, 3H, C*H$_3$*), 1.37 – 1.30 (m, 10H, C*H$_2$* & C*H$_3$*), 1.28 (d, *J* = 6.7, 3H, C*H$_3$*), 1.27 (d, *J* = 6.7, 3H, C*H$_3$*), 1.19 – 1.15 (m, 6H, C*H$_3$*), 1.13 – 1.09 (m, 4H, C*H$_2$* & C*H$_3$*), 1.05 (s, 3H, C*H$_3$*), 0.84 (s, 3H, C*H$_3$*). ^{11}B NMR (**128 MHz, benzene-*d$_6$***) δ 42.61 (br), 29.22 (br). **^{13}C{^{1}H} NMR (101 MHz, benzene-*d$_6$*)** δ 148.0 (*C*-Ar), 147.6 (*C*-Ar), 146.8 (*C*-Ar), 145.6 (*C*-Ar), 143.2 (*C*-Ar), 133.3 (*C*-Ar), 129.2 (*C*H-Ar), 126.0 (*C*H-Ar), 124.4 (*C*H-Ar), 124.4 (*C*H-Ar), 124.0 (*C*H-Ar), 123.6 (*C*H-Ar), 74.2 (*C*-Alkyl), 58.0 (*C*H_2), 56.7 (*C*-Alkyl), 54.5 (*C*H_2), 53.0 (*C*-Alkyl), 38.3 (*C*H_2), 37.5 (*C*H_3), 36.7 (*C*H_3), 36.6 (*C*H_3), 35.3 (*C*H_3), 31.5 (*C*H_3), 30.3 (*C*H_3), 29.4 (*C*H(CH_3)$_2$), 29.1 (*C*H_3), 28.9 (*C*H(CH_3)$_2$), 28.3 (*C*H(CH_3)$_2$), 28.2 (*C*H(CH_3)$_2$), 27.3 (*C*H_3), 26.5 (*C*H_3), 26.1 (*C*H_3), 25.8 (*C*H_3), 25.5 (*C*H_3), 25.0 (*C*H_3), 24.8 (*C*H_3), 23.3 (*C*H_3), 22.9 (*C*H_3). **LIFDI-MS:** *m/z* calcd. For [$C_{40}H_{62}B_2N_2$]: cal: 592.5094; found: 592.5086.

Synthesis of compound 11 and 11':

AgOTf (27 mg, 0.10 mol) was slowly added to a benzene solution of **10** (42 mg, 0.071 mmol) under vigorous stirring to give a red suspension in 10 min. After the reaction, all the volatiles were removed under reduced pressure to give a red solid. The residue was dissolved in dichloromethane and slow evaporation of the solution provided **11** and **11'** as colorless crystals (41 mg, 70%) (*Despite multiple attempts, we were unable to separate the two isomers by recrystallization*).

^{1}H NMR (400 MHz, DCM-d_2) δ 7.61 – 7.50 (m, Ar*H*), 7.44 – 7.17 (m, Ar*H*), 4.11 (s, B*H*), 3.83 (s, B*H*), 3.43 (t, sep, *J* = 6.7 Hz, C*H*(CH_3)$_2$), 3.34 (sep, *J* = 6.7 Hz, C*H*(CH_3)$_2$), 3.21 (sep, *J* = 6.7 Hz, C*H*(CH_3)$_2$), 3.11 (sep, *J* = 6.7 Hz, C*H*(CH_3)$_2$), 2.79 (sep, *J* = 6.7 Hz, C*H*(CH_3)$_2$), 2.56 (sep, *J* = 6.7 Hz, C*H*(CH_3)$_2$), 2.29 – 2.18 (m, CH_2), 2.08 – 1.84 (m, CH_2 & CH_3), 1.58 (s, CH_3), 1.47 (s, CH_3), 1.43 – 1.10 (m, CH_2 & CH_3), 0.97 (s, CH_3). **^{11}B NMR (128 MHz, DCM-d_2)** δ 39.88 (br), 20.77 (br). **^{19}F NMR (377 MHz, DCM-d_2)** δ −77.70. **^{13}C{^{1}H} NMR (101 MHz, DCM-d_2)** δ 148.6 (*C*-Ar), 148.0 (*C*-Ar), 147.2 (*C*-Ar), 147.1 (*C*-Ar), 146.5 (*C*-Ar), 145.7 (*C*-Ar), 145.5 (*C*-Ar), 145.1 (*C*-Ar), 140.2 (*C*-Ar), 131.8 (*C*-Ar), 131.3 (*C*-Ar), 130.5 (*C*H-Ar), 130.5 (*C*H-Ar), 126.8 (*C*H-Ar), 125.6 (*C*H-Ar), 125.6 (*C*H-Ar), 125.2 (*C*H-Ar), 124.9 (*C*H-Ar), 124.7 (*C*H-Ar), 124.3 (*C*H-Ar), 124.2 (*C*H-Ar), 122.1 (*C*F_3), 118.9 (*C*F_3), 79.2 (*C*-Alkyl), 78.7 (*C*-Alkyl), 58.0 (*C*-Alkyl), 57.8 (*C*-Alkyl), 57.3 (*C*H_2), 57.0 (*C*H_2), 55.6 (*C*H_2), 54.7 (*C*-Alkyl), 52.5 (*C*-Alkyl), 51.6 (*C*H_2), 39.8 (*C*H_2), 37.3 (*C*H_3), 37.2 (*C*H_3), 37.2 (*C*H_3), 36.8 (*C*H_3), 36.6 (*C*-Alkyl), 36.6 (*C*-Alkyl), 36.1 (*C*H_3), 36.0 (*C*H_3), 35.5 (*C*H_3), 35.4 (*C*H_3), 34.9 (*C*H_2), 33.5 (*C*H_3), 33.3 (*C*H_3), 33.2 (*C*H_3), 32.1 (*C*H_3), 31.2 (*C*H_3), 31.0 (*C*H_3), 30.3 (*C*H(CH_3)$_2$), 30.0 (*C*H(CH_3)$_2$), 29.5 (*C*H_3), 29.2 (*C*H(CH_3)$_2$), 29.0 (*C*H(CH_3)$_2$), 28.7 (*C*H(CH_3)$_2$), 28.6 (*C*H(CH_3)$_2$), 28.6 (*C*H_3), 28.5 (*C*H_3), 28.4 (*C*H(CH_3)$_2$), 27.5 (*C*H_3), 27.1 (*C*H_3), 27.0 (*C*H_3), 26.8 (*C*H_3), 26.5 (*C*H_3), 26.5 (*C*H_3), 26.4 (*C*H_3), 26.3 (*C*H_3), 25.3 (*C*H_3), 24.8 (*C*H_3), 24.6 (*C*H_3), 24.5 (*C*H_3), 24.4 (*C*H_3), 23.5 (*C*H_3), 23.1 (*C*H_3), 23.0 (*C*H_3), 22.9 (*C*H_3). **LIFDI-MS:** *m/z* calcd. For the monomer [$C_{40}H_{62}B_2N_2AgCF_3SO_3$]: cal: 848.3665; found: 850.3661.

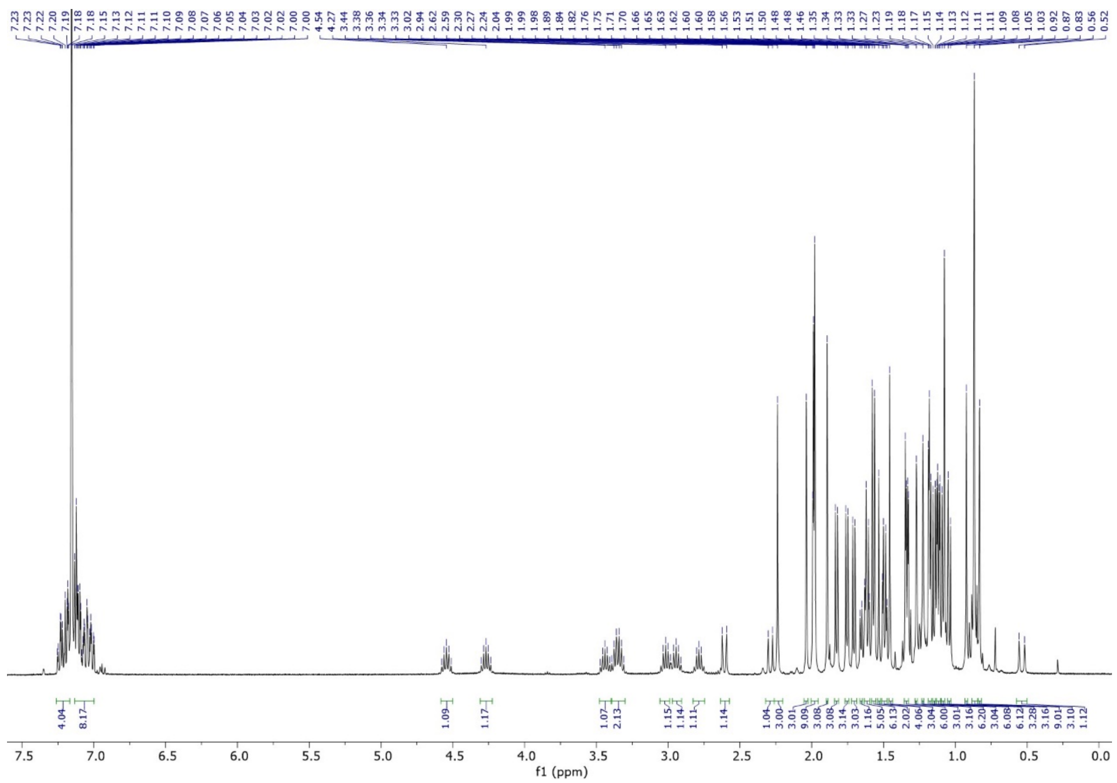

Figure S1. ^{1}H NMR spectrum of **1**.

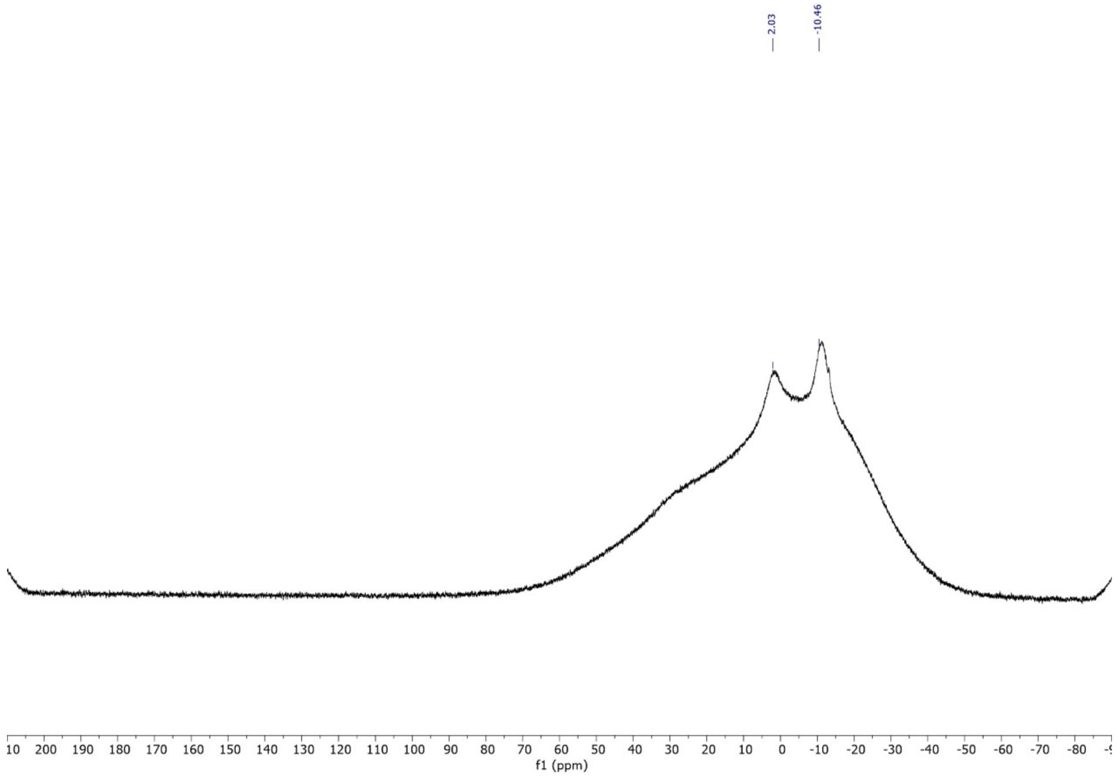

Figure S2. ^{11}B NMR spectrum of **1**.

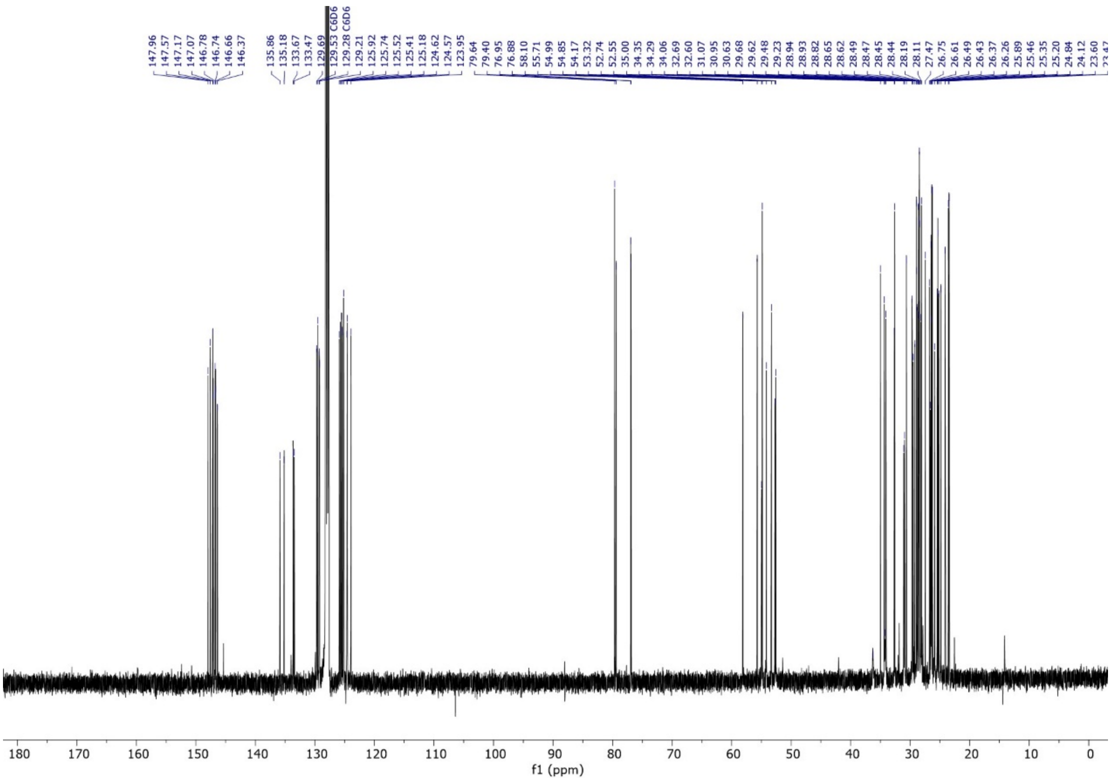

Figure S3. $^{13}C\{^{1}H\}$ NMR spectrum of **1**.

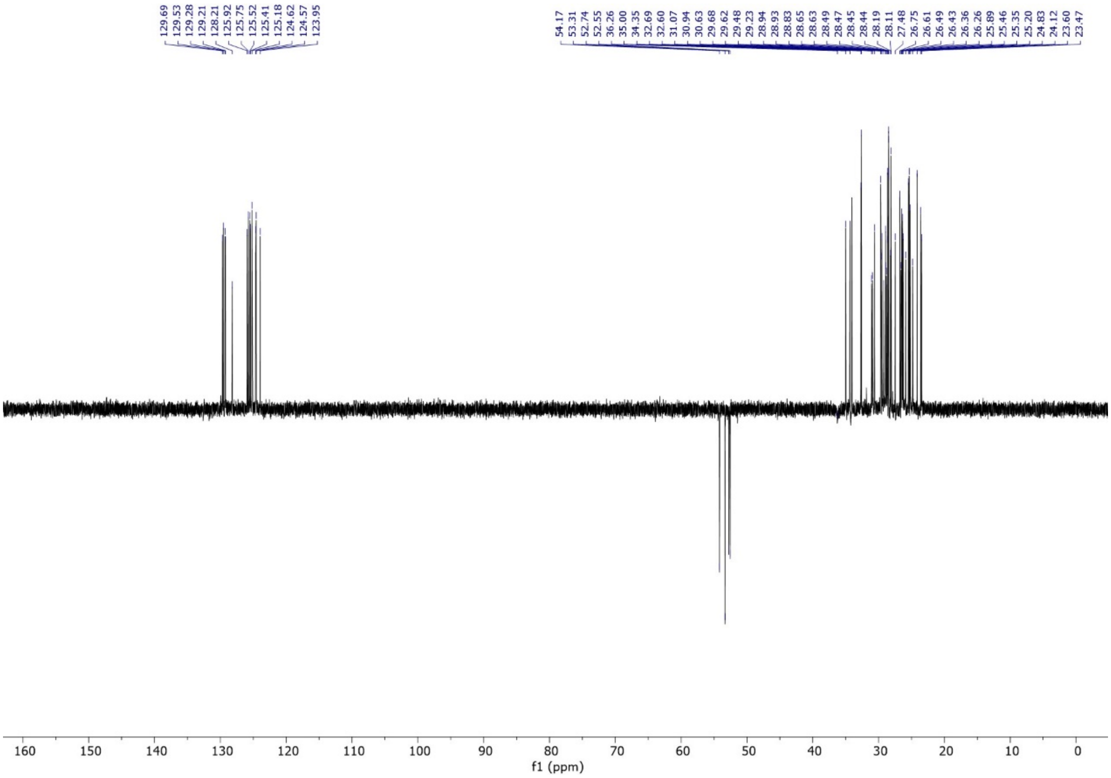

Figure S4. $^{13}C\{^{1}H\}$ NMR (DEPT 135) spectrum of **1**.

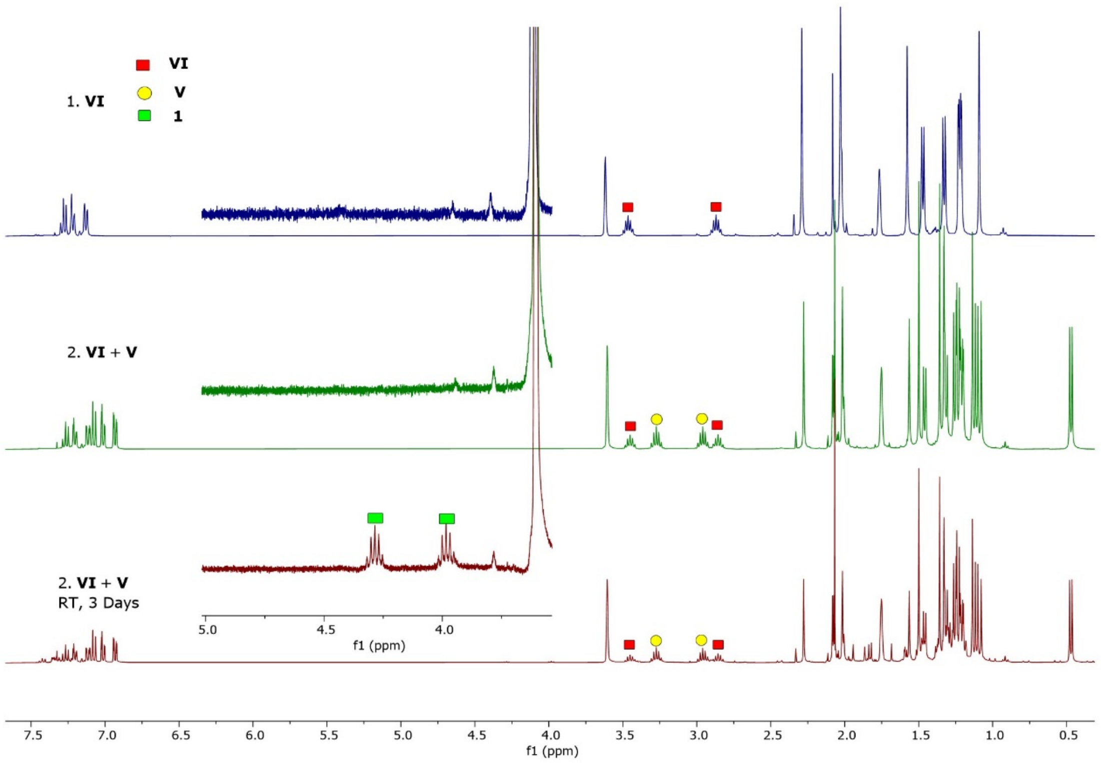

Figure S5. ^{1}H NMR spectra of the reaction of **VI** and **V** at RT.

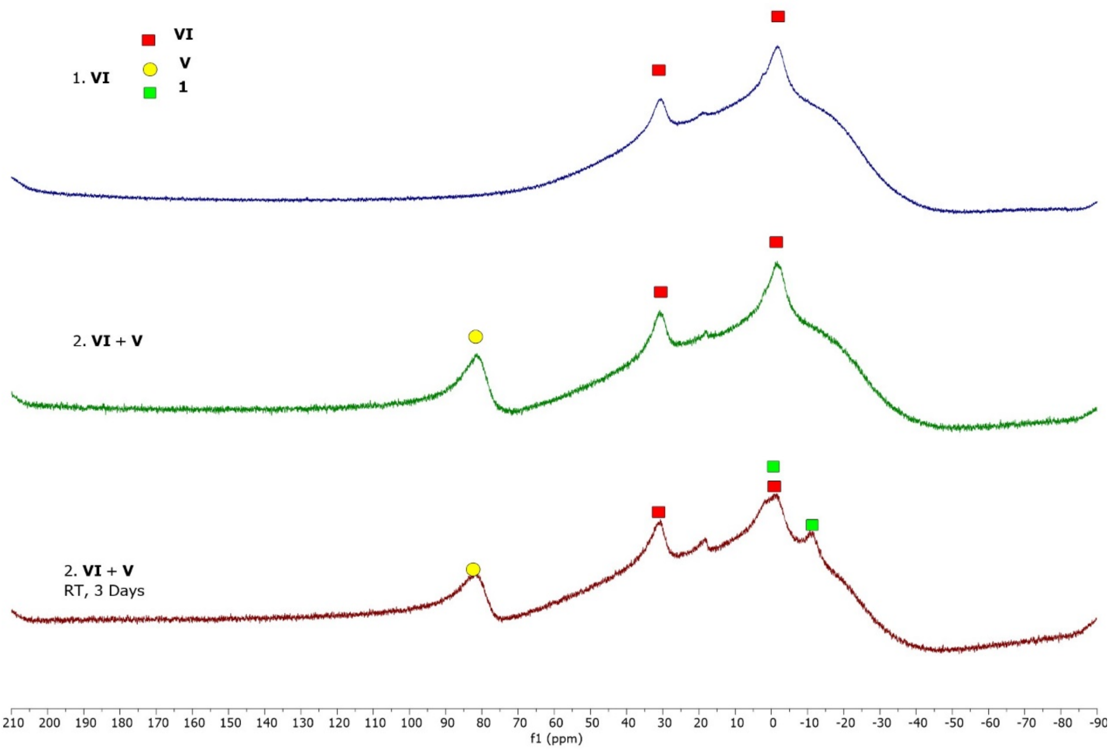

Figure S6. ^{11}B NMR spectra of the reaction of **VI** and **V** at RT.

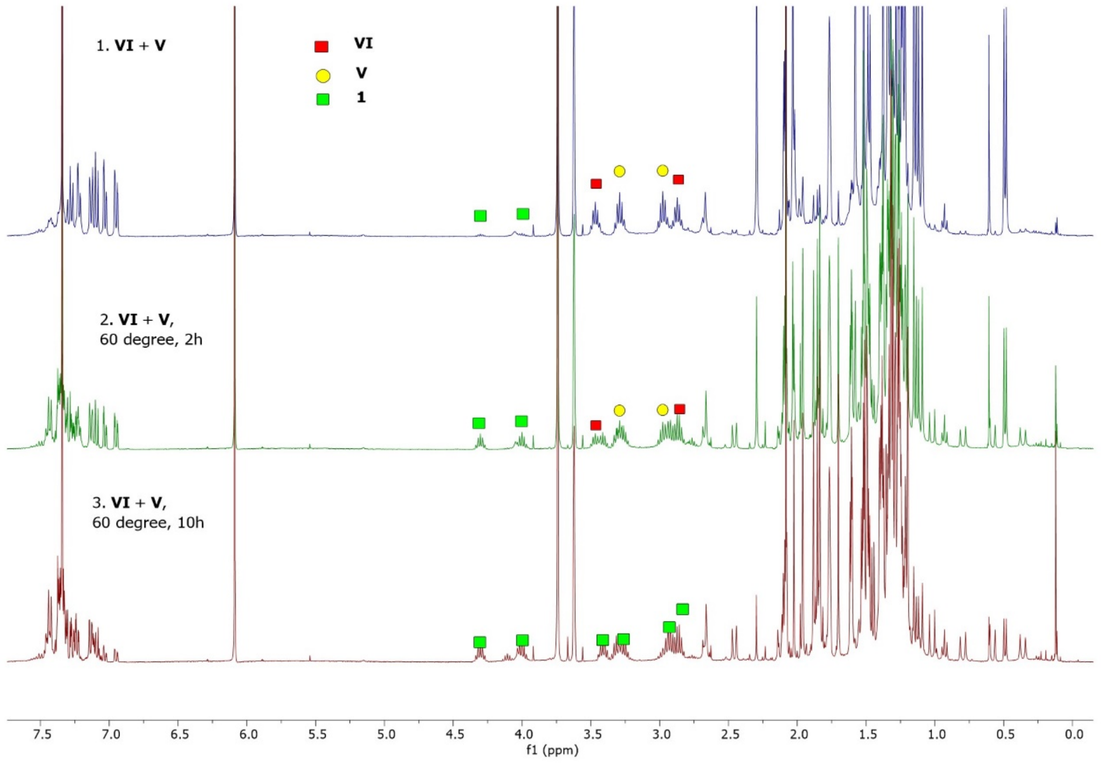

Figure S7. ^{1}H NMR spectra of the reaction of **VI** and **V** at 60 °C.

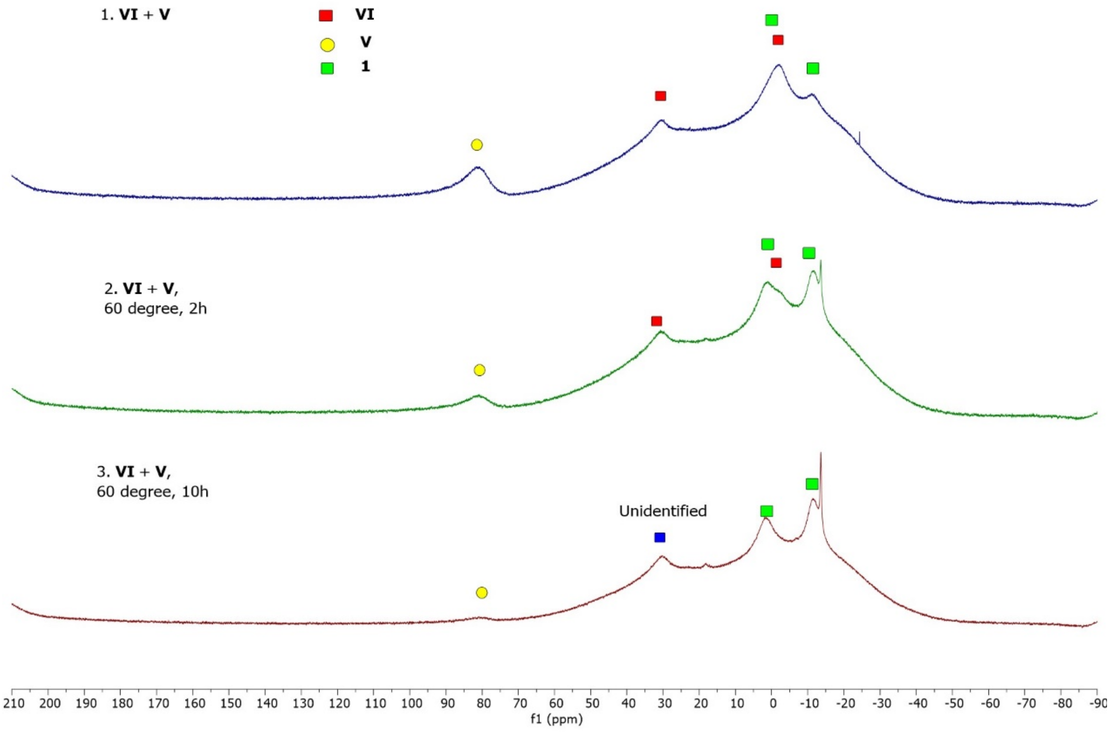

Figure S8. ^{11}B NMR spectra of the reaction of **VI** and **V** at 60 °C.

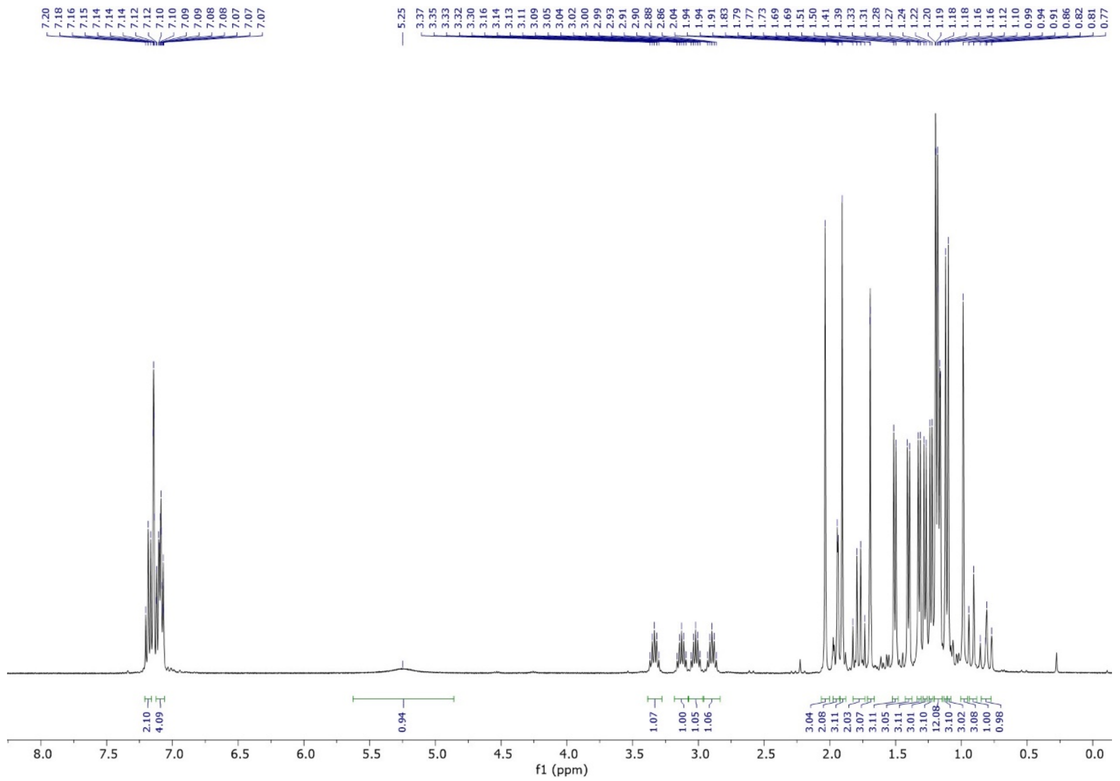

Figure S9. ^{1}H NMR spectrum of **2**.

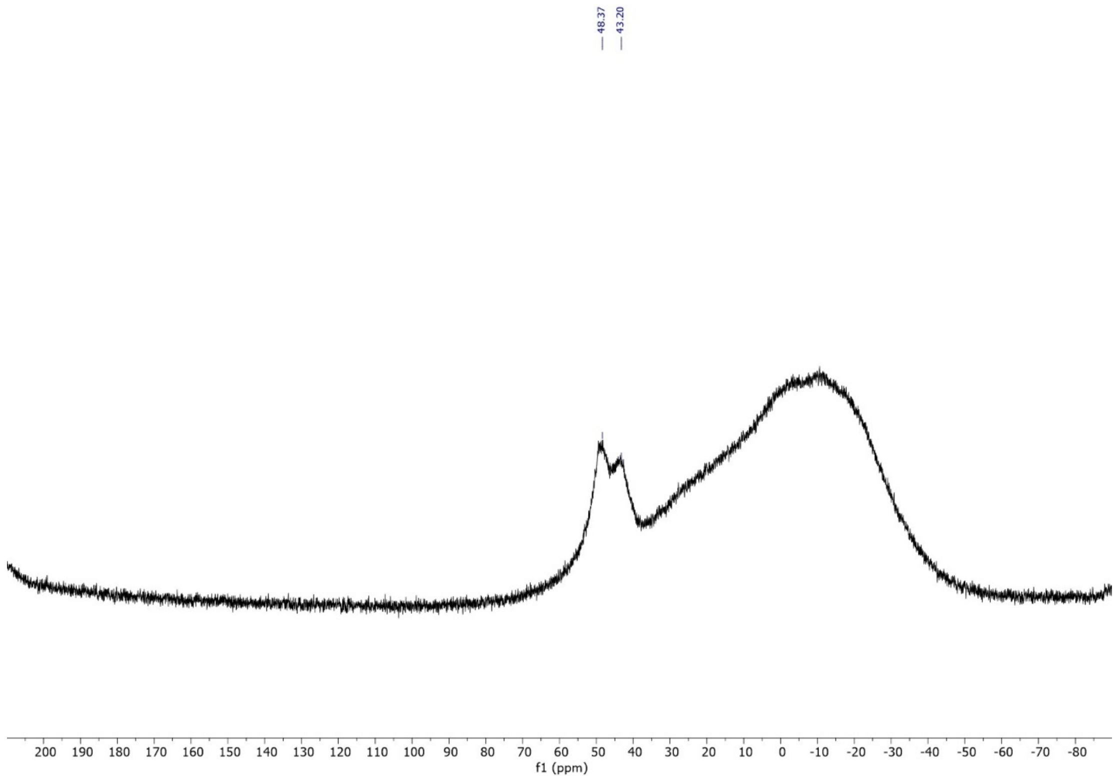

Figure S10. ^{11}B NMR spectrum of **2**.

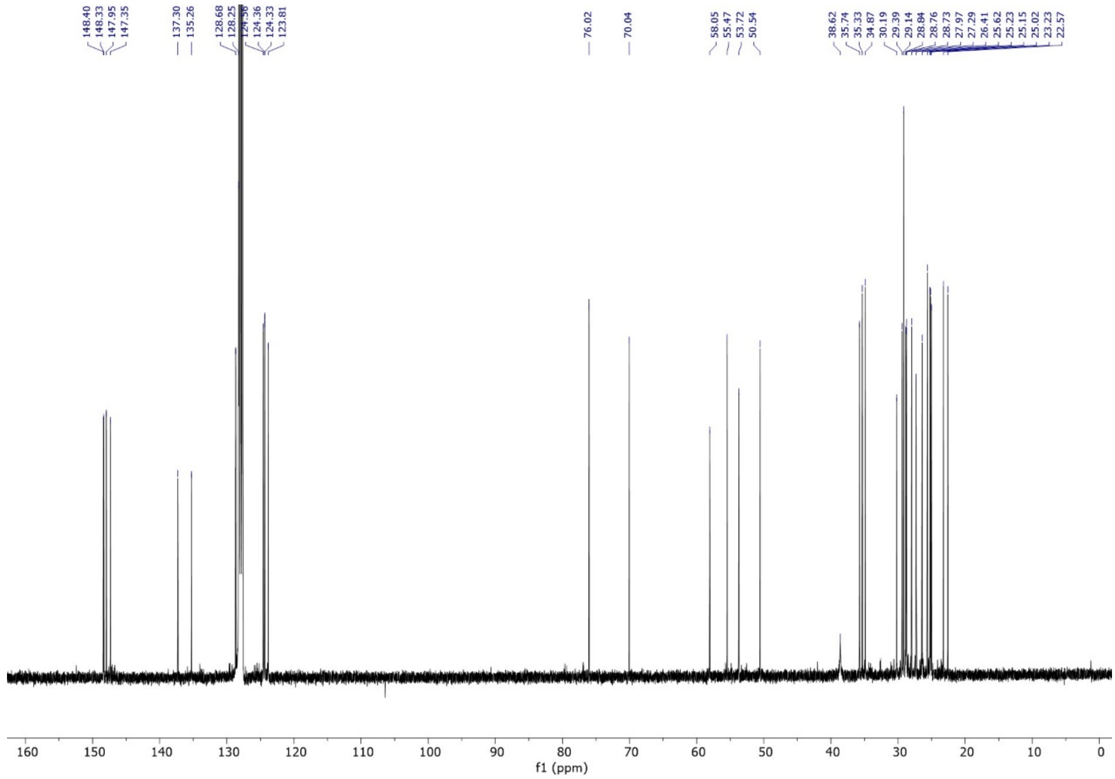

Figure S11. $^{13}C\{^{1}H\}$ NMR spectrum of **2**.

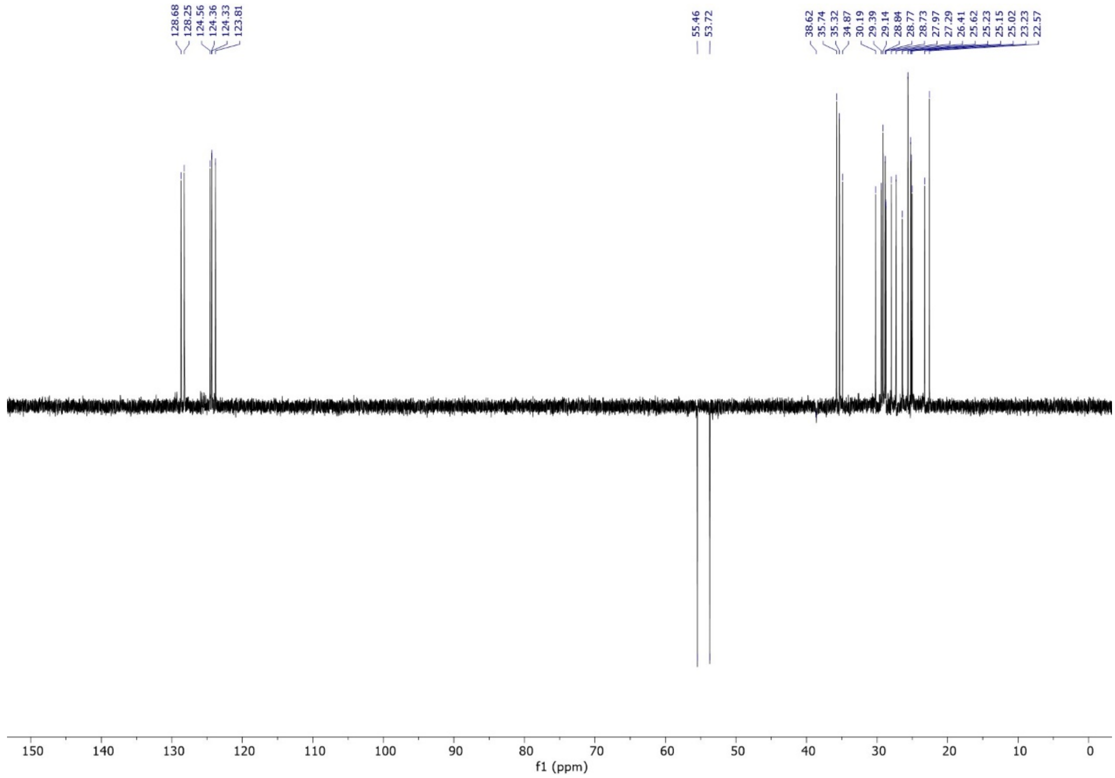

Figure S12. $^{13}C\{^{1}H\}$ NMR (DEPT 135) spectrum of **2**.

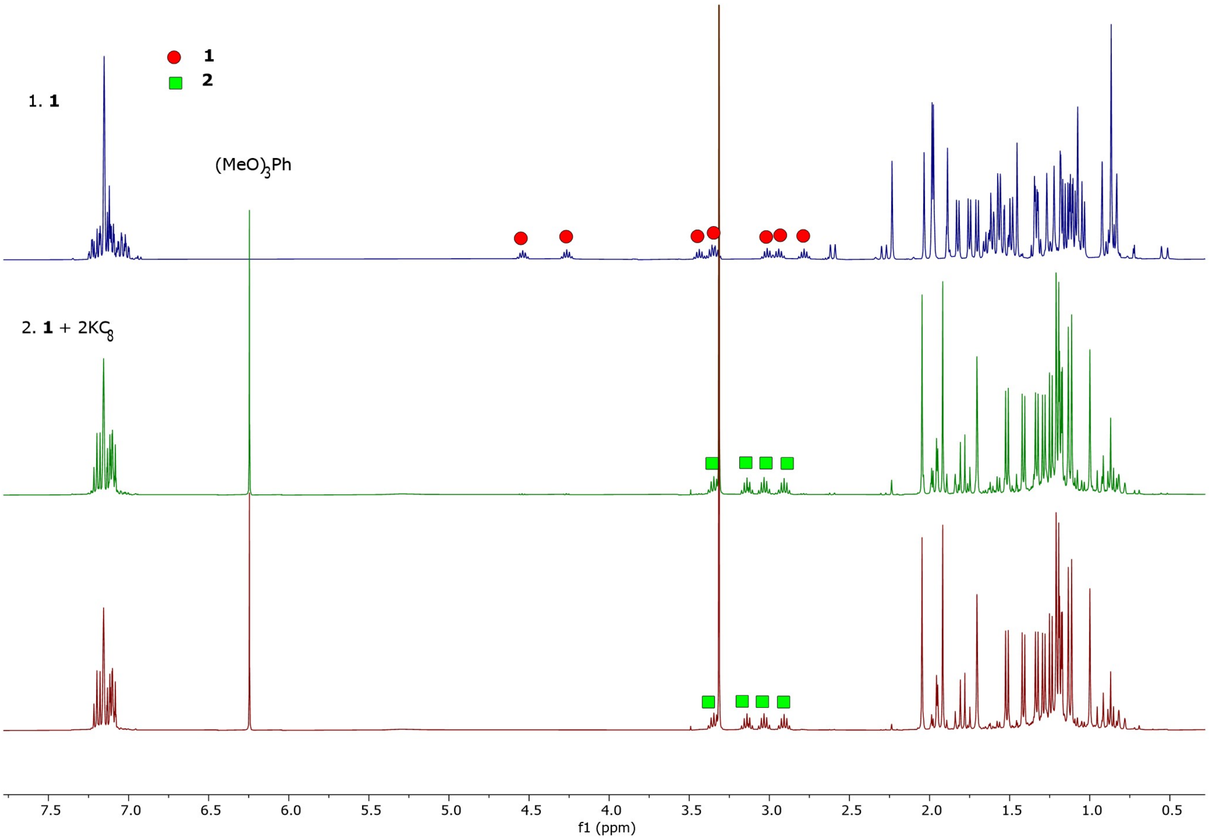

Figure S13. ^{1}H NMR spectra of the reductive conversion of **1** to **2** with KC_8.

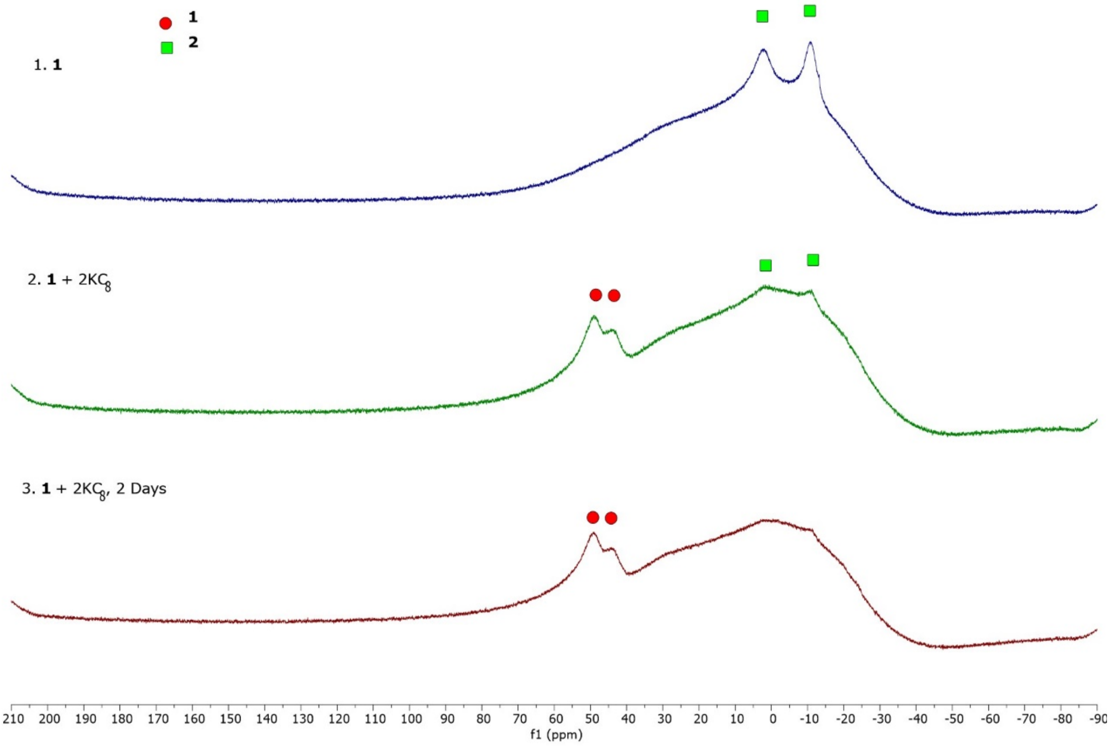

Figure S14. ^{11}B NMR spectra of the reductive conversion of **1** to **2** with KC_8.

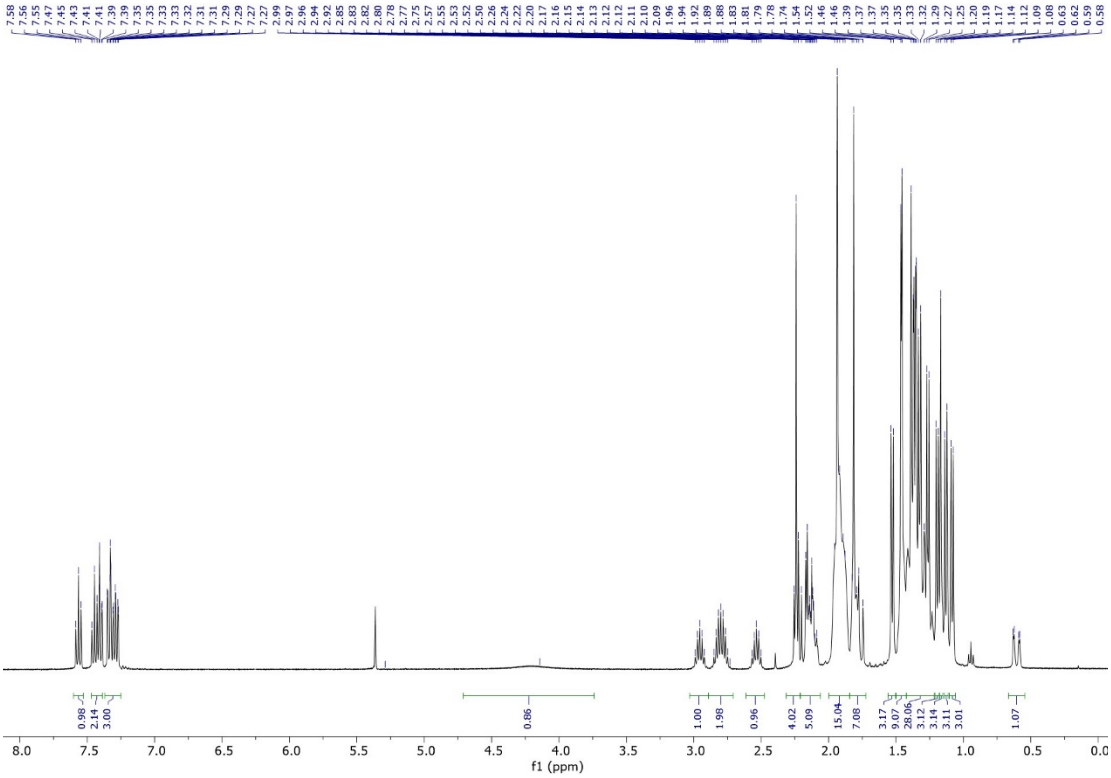

Figure S15. ^{1}H NMR spectrum of **3**.

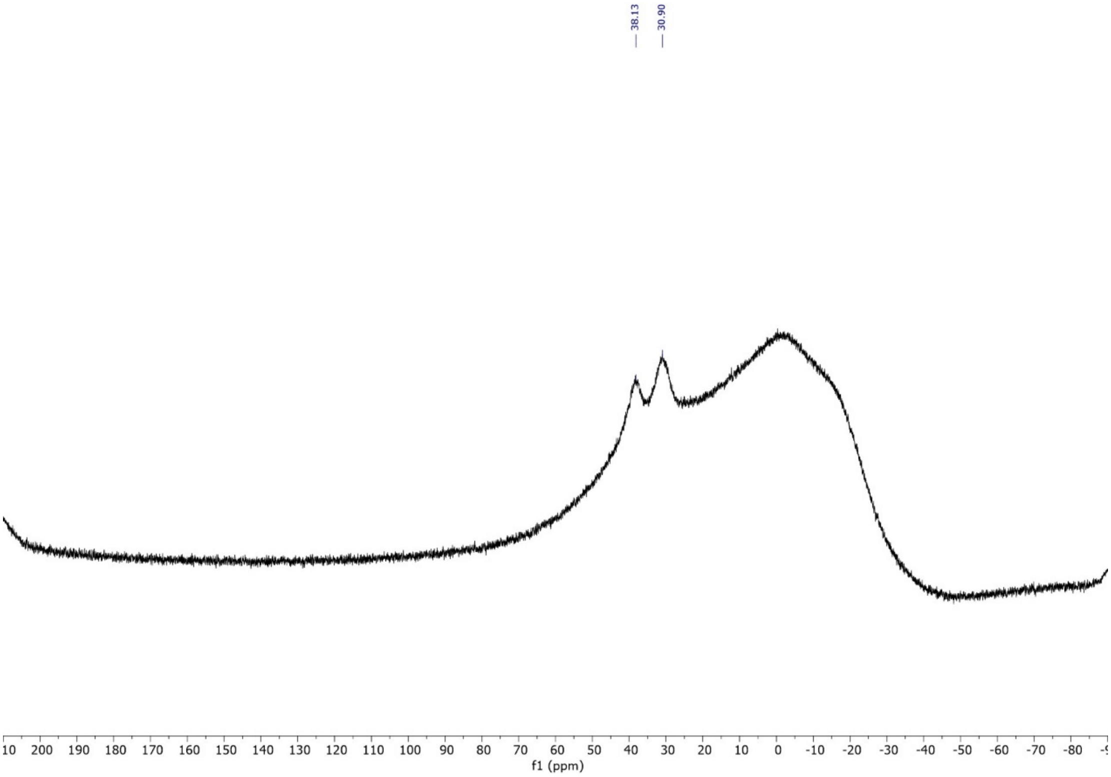

Figure S16. ^{11}B NMR spectrum of **3**.

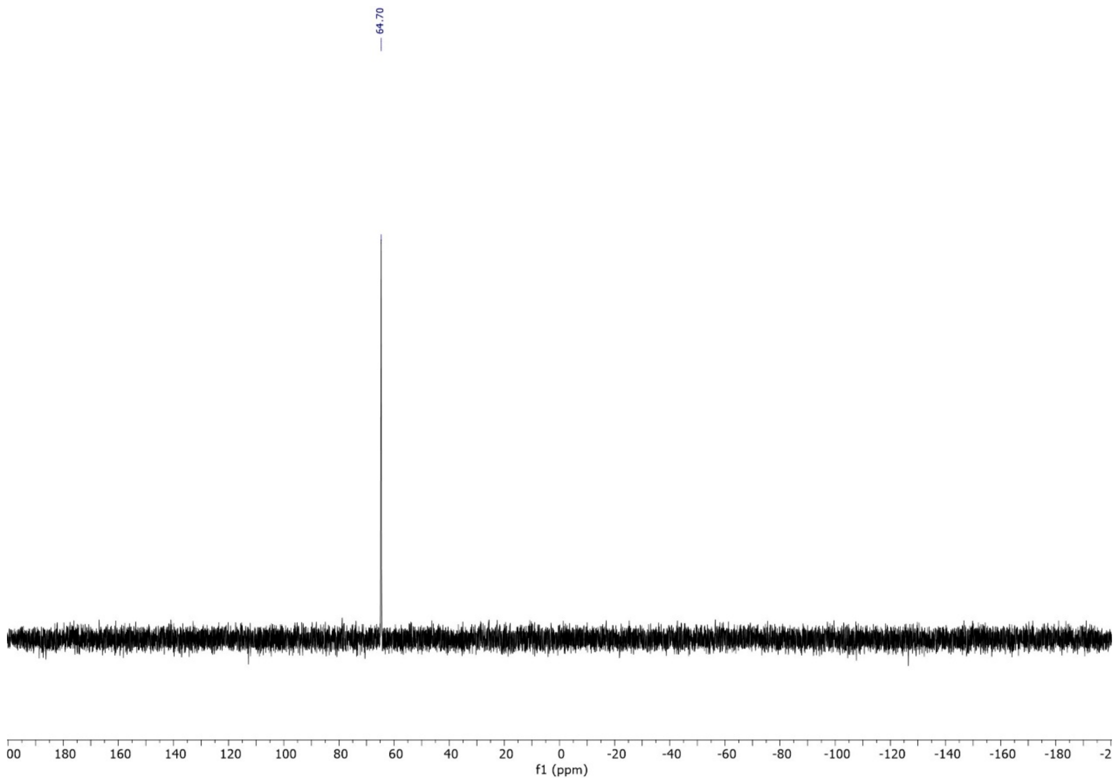

Figure S17. ^{31}P NMR spectrum of **3**.

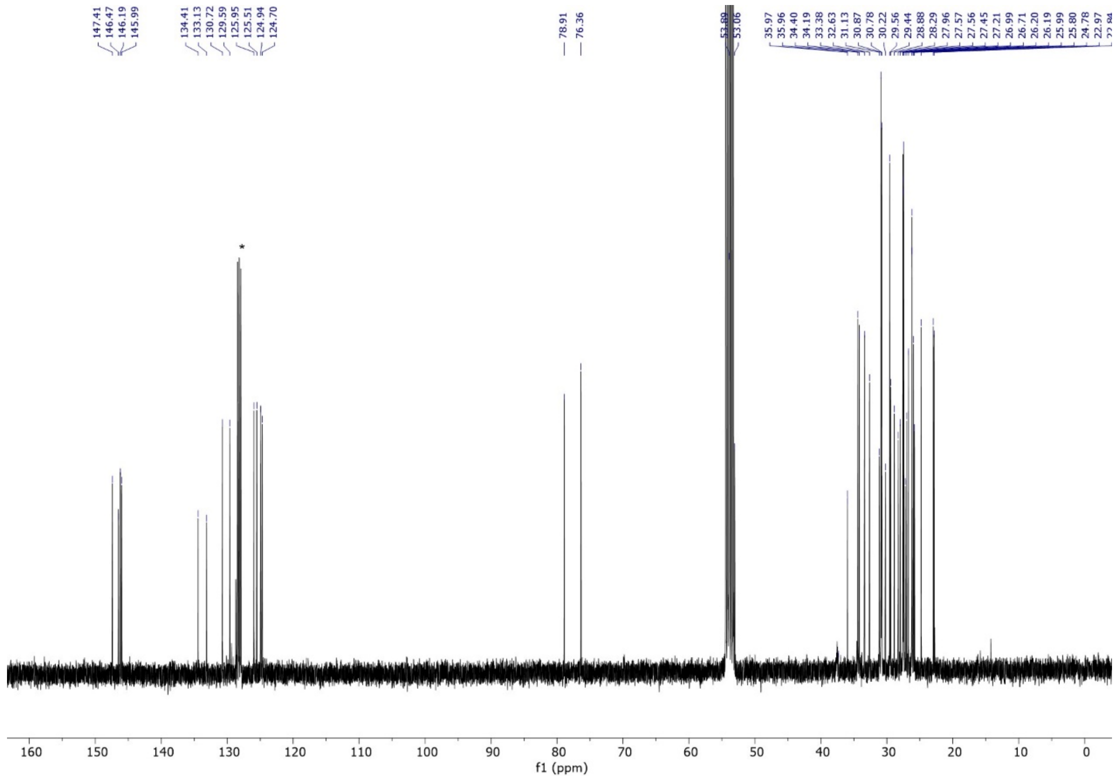

Figure S18. ^{13}C{^{1}H} NMR spectrum of **3** (*benzene).

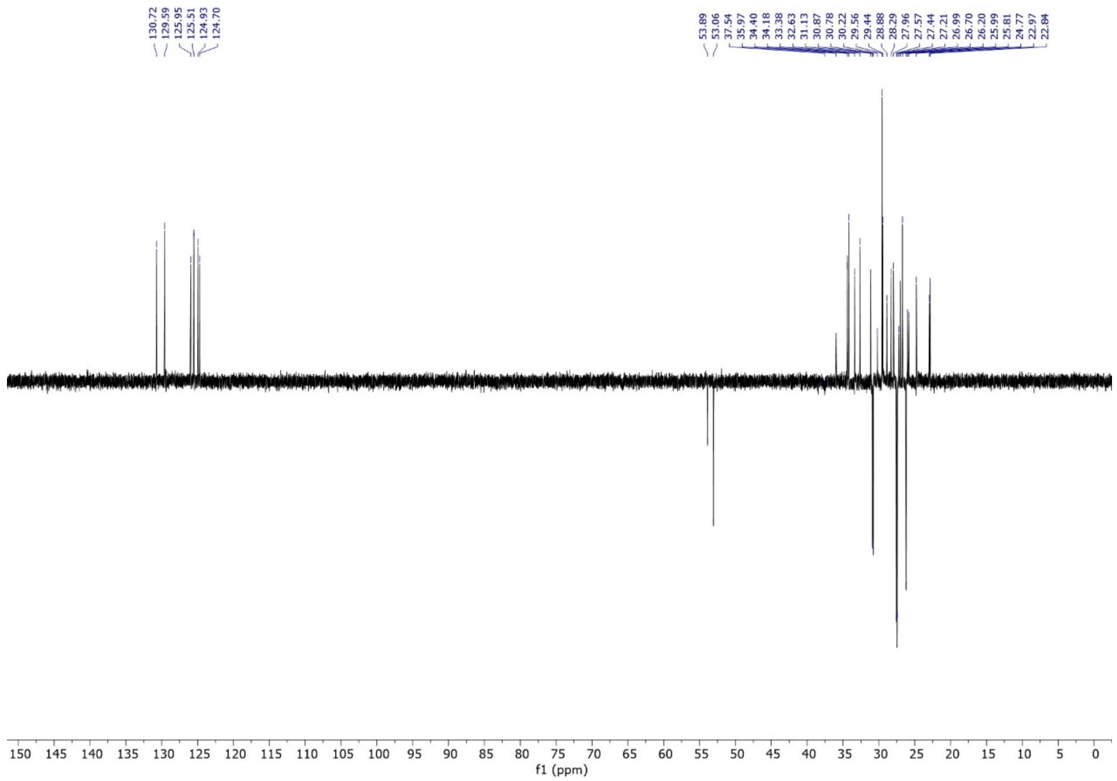

Figure S19. $^{13}C\{^{1}H\}$ NMR (DEPT 135) spectrum of **3**.

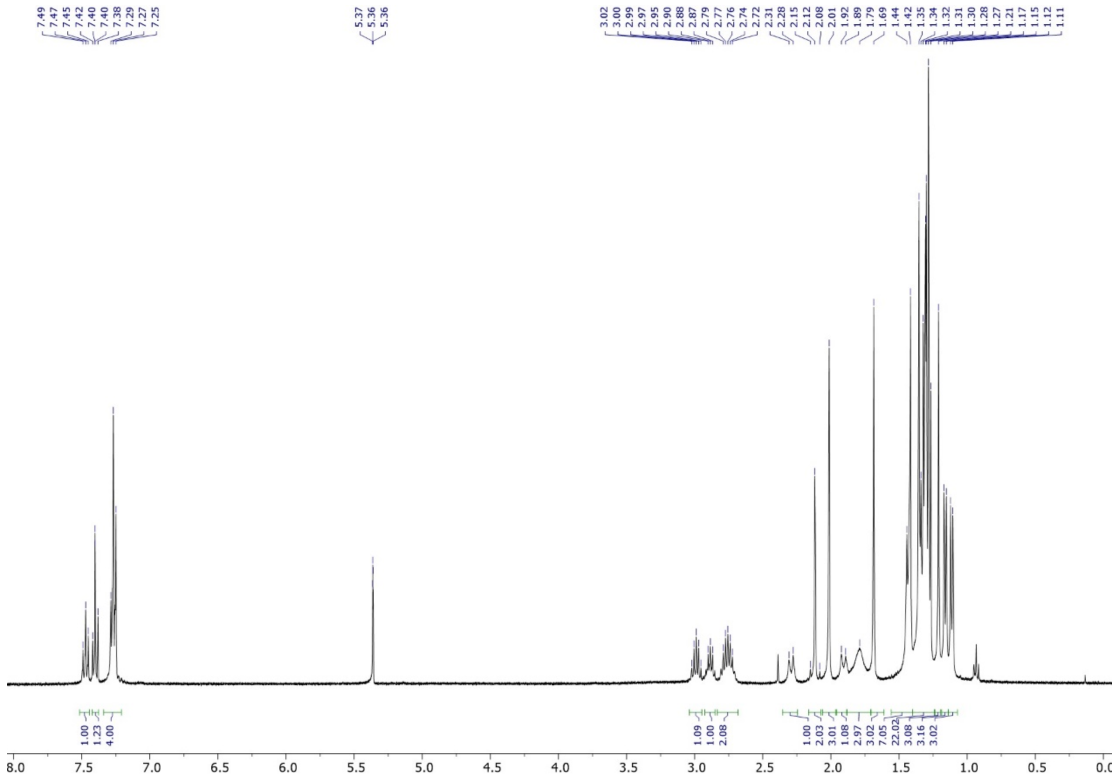

Figure S20. ^{1}H NMR spectrum of **4**.

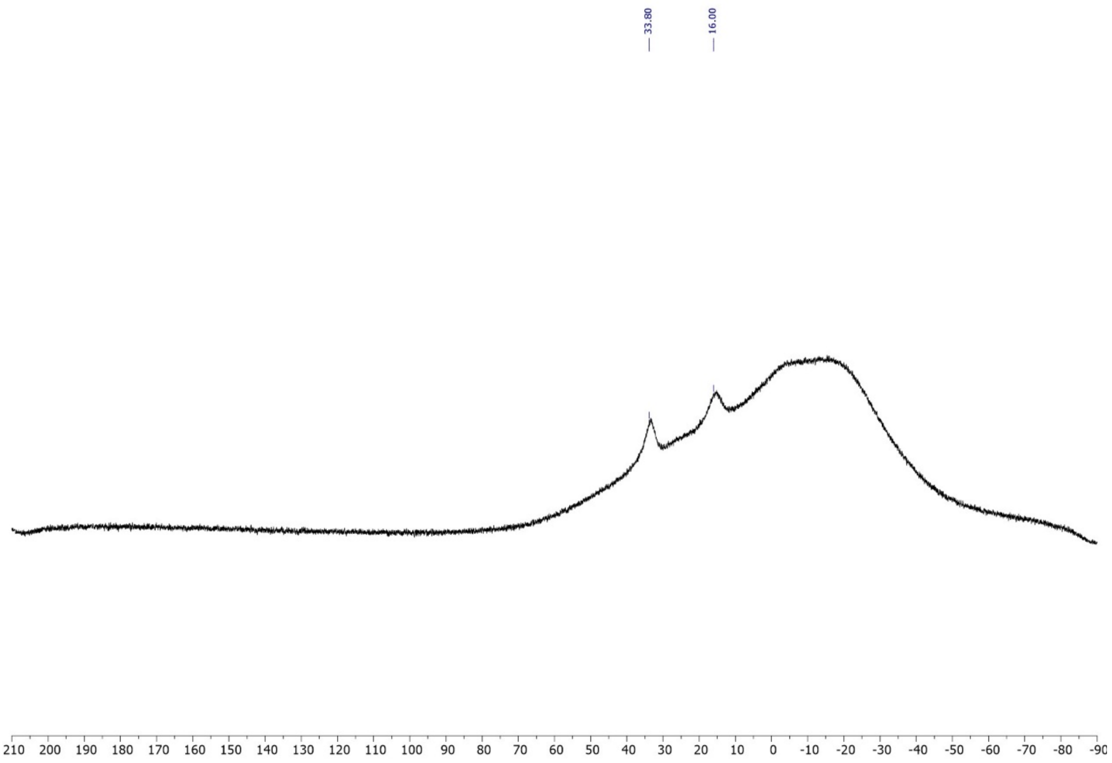

Figure S21. ^{11}B NMR spectrum of **4**.

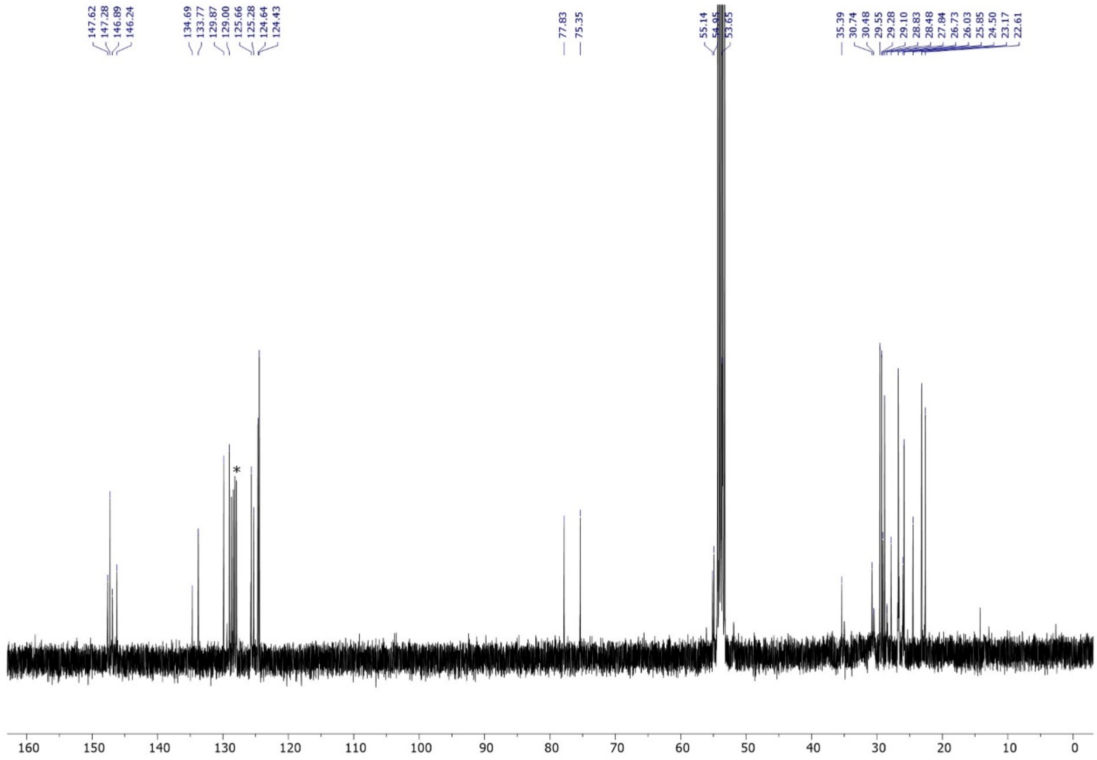

Figure S22. ^{13}C{^{1}H} NMR spectrum of **4** (*benzene).

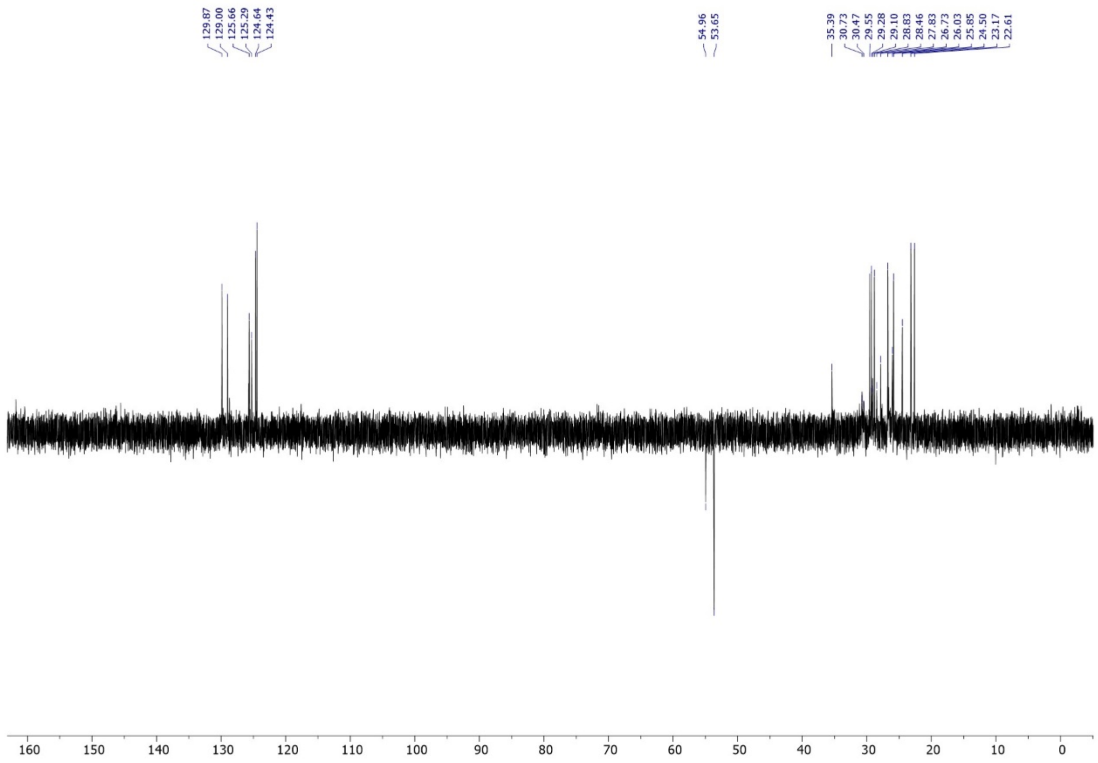

Figure S23. $^{13}C\{^{1}H\}$ NMR (DEPT 135) spectrum of **4**.

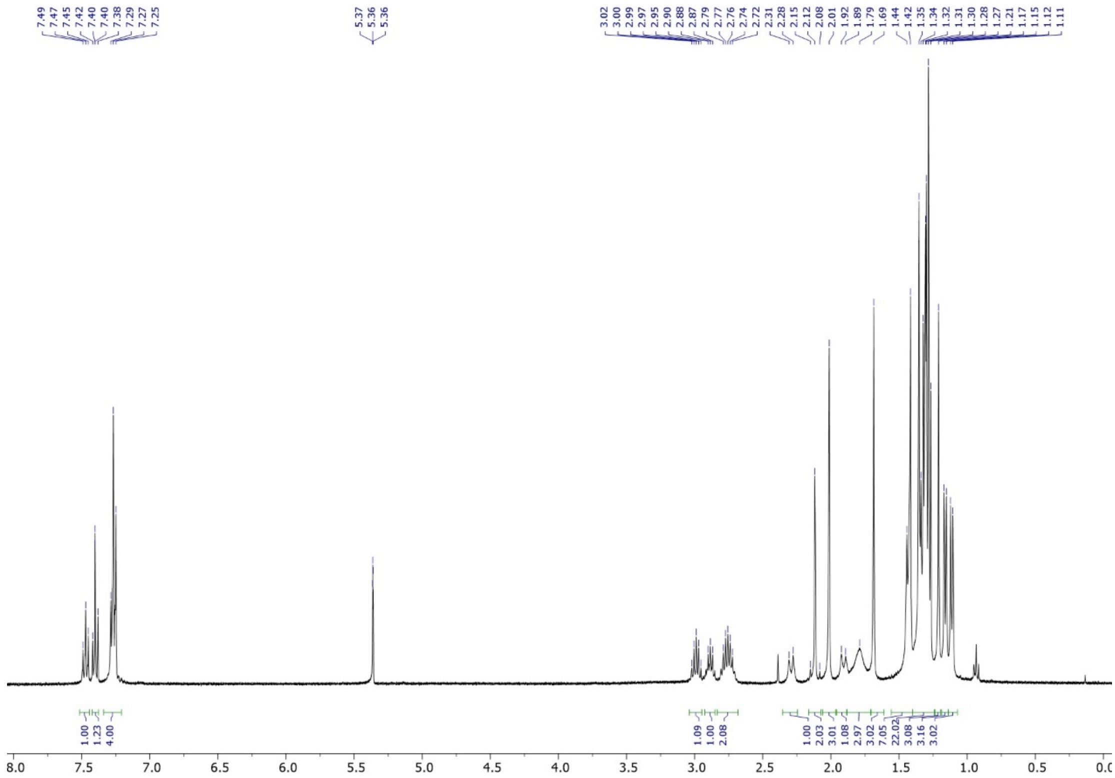

Figure S24. ^{1}H NMR spectrum of **4**.

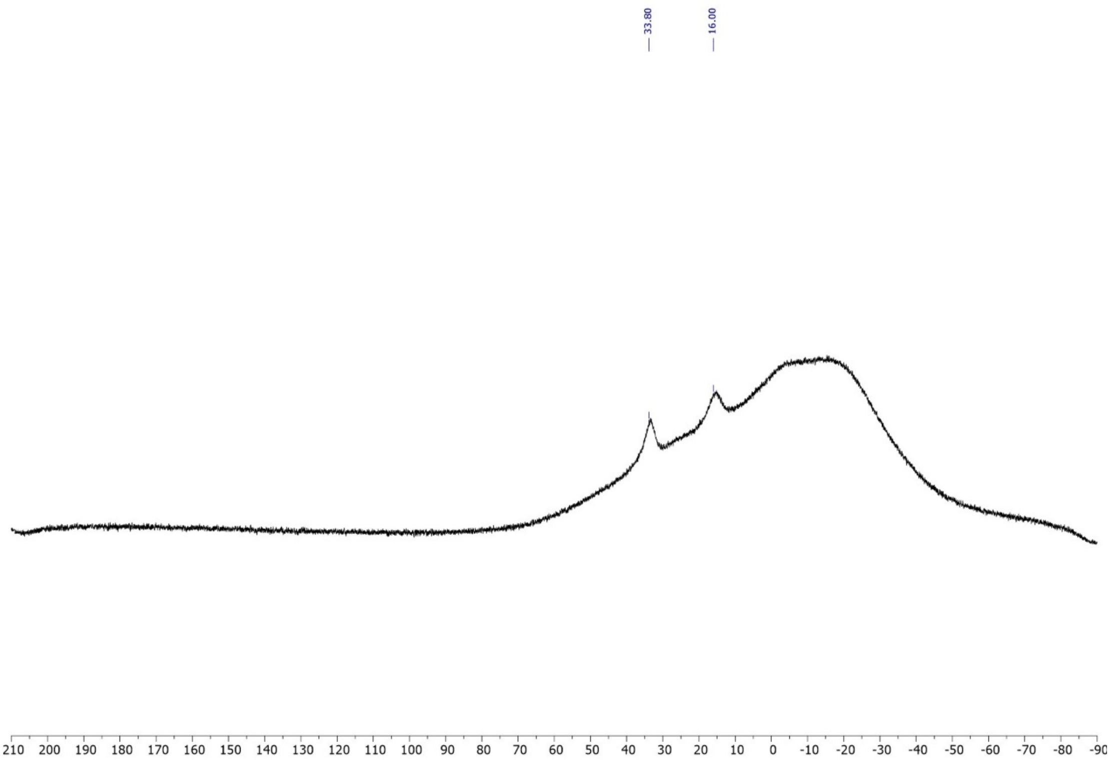

Figure S25. ^{11}B NMR spectrum of **4**.

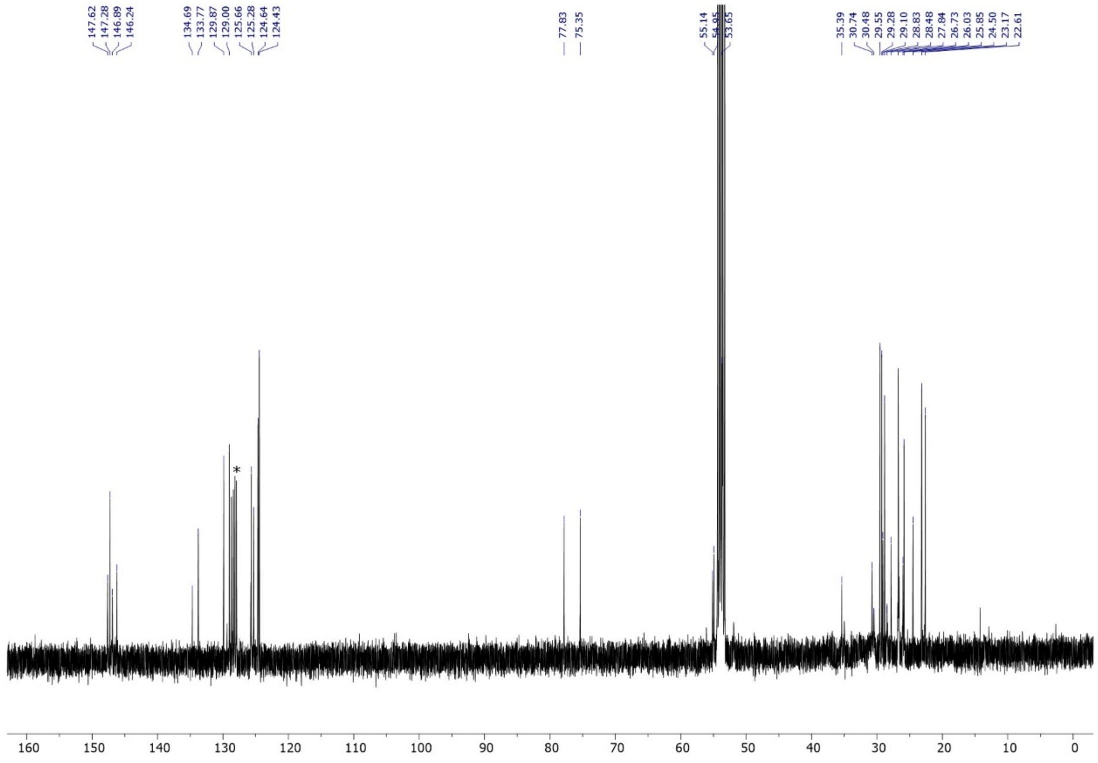

Figure S26. $^{13}C\{^1H\}$ NMR spectrum of **4** (*benzene).

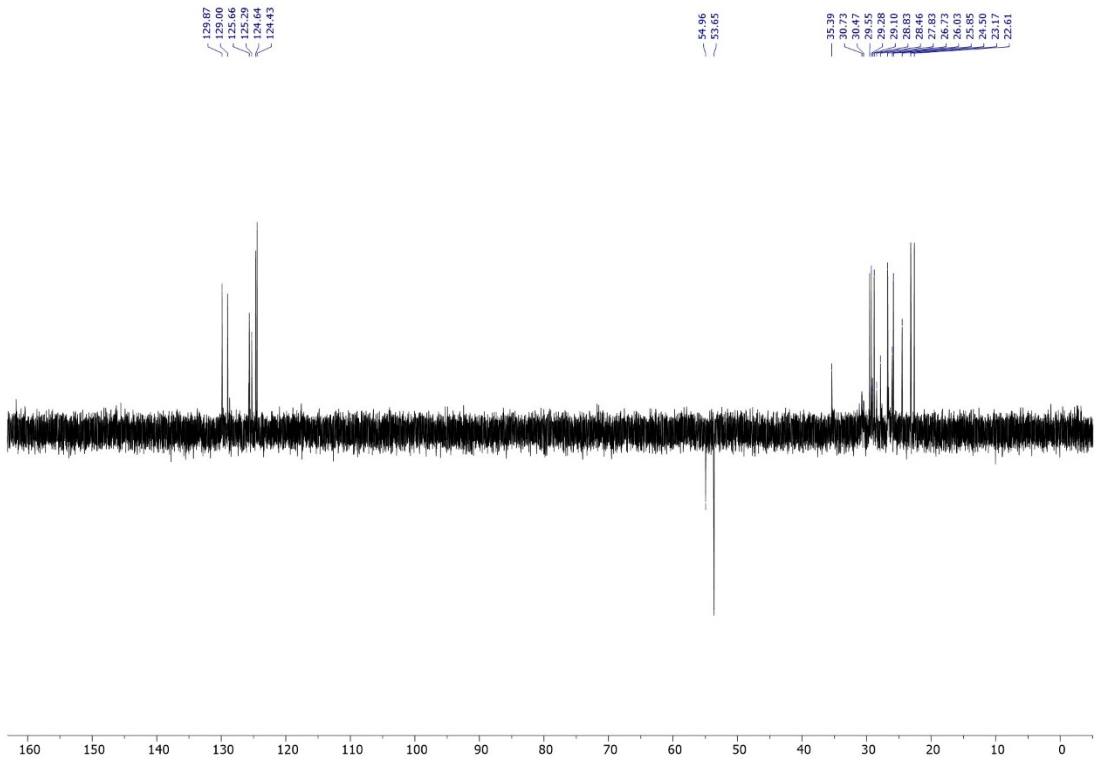

Figure S27. $^{13}C\{^{1}H\}$ NMR (DEPT 135) spectrum of **4**.

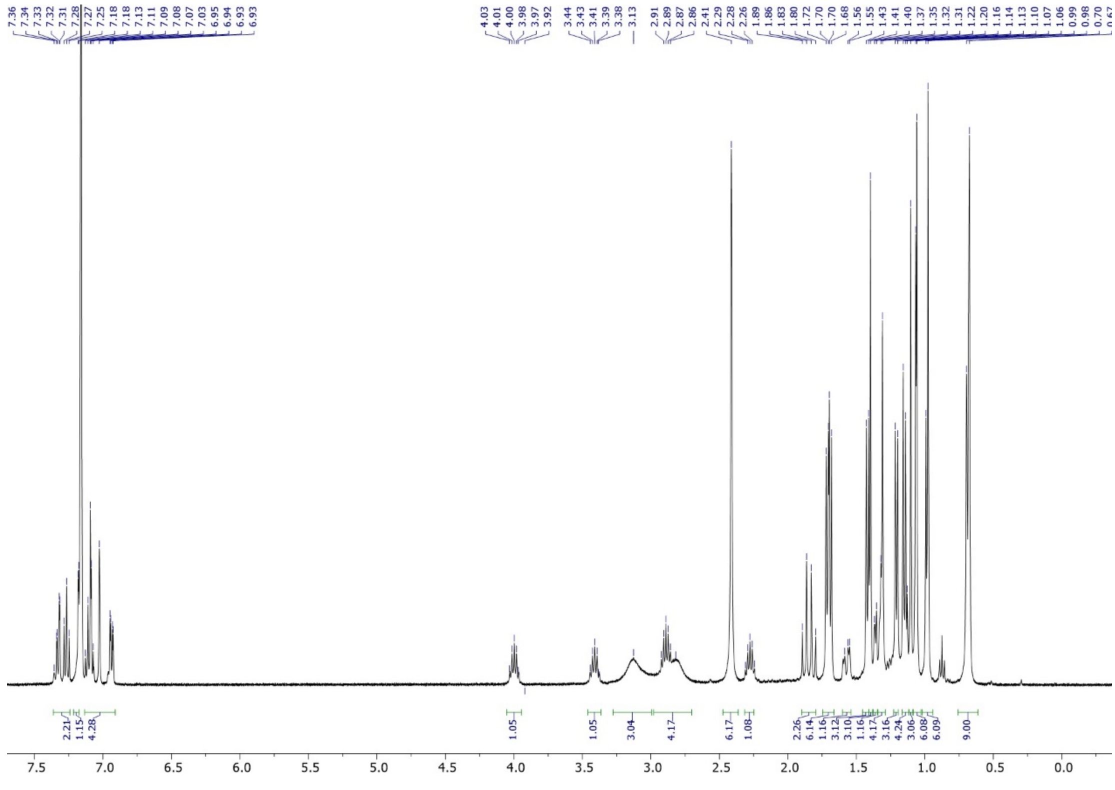

Figure S28. ^{1}H NMR spectrum of **6**.

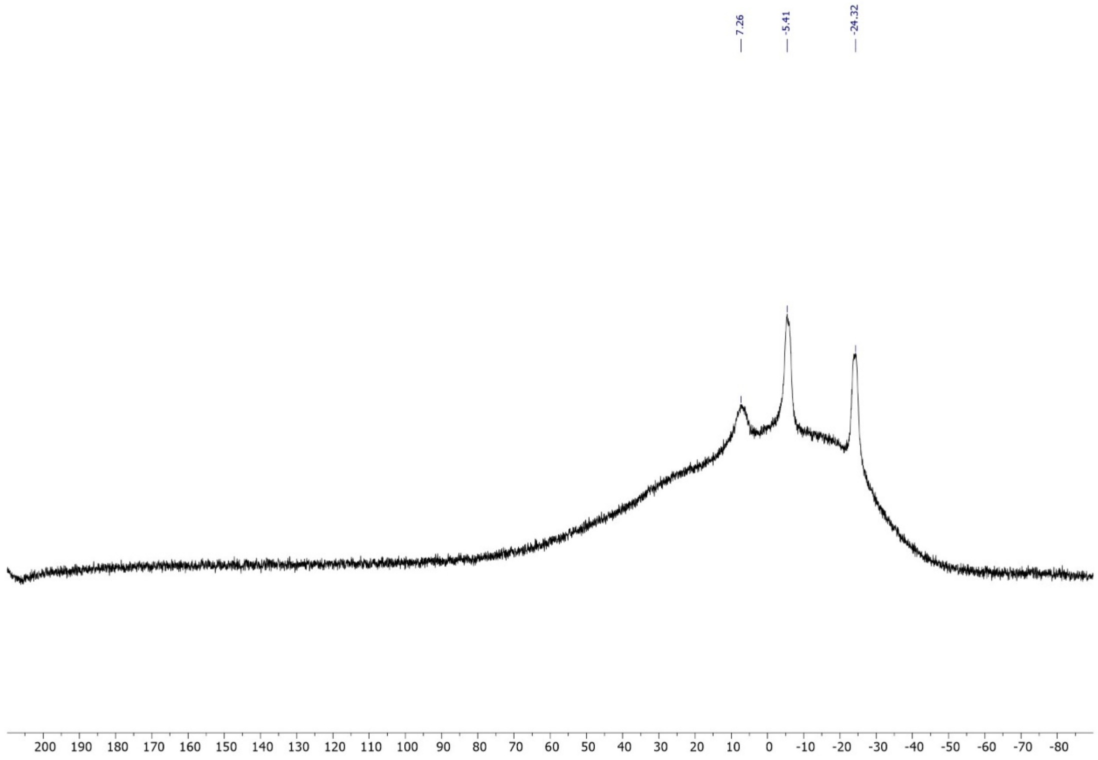

Figure S29. ^{11}B NMR spectrum of **6**.

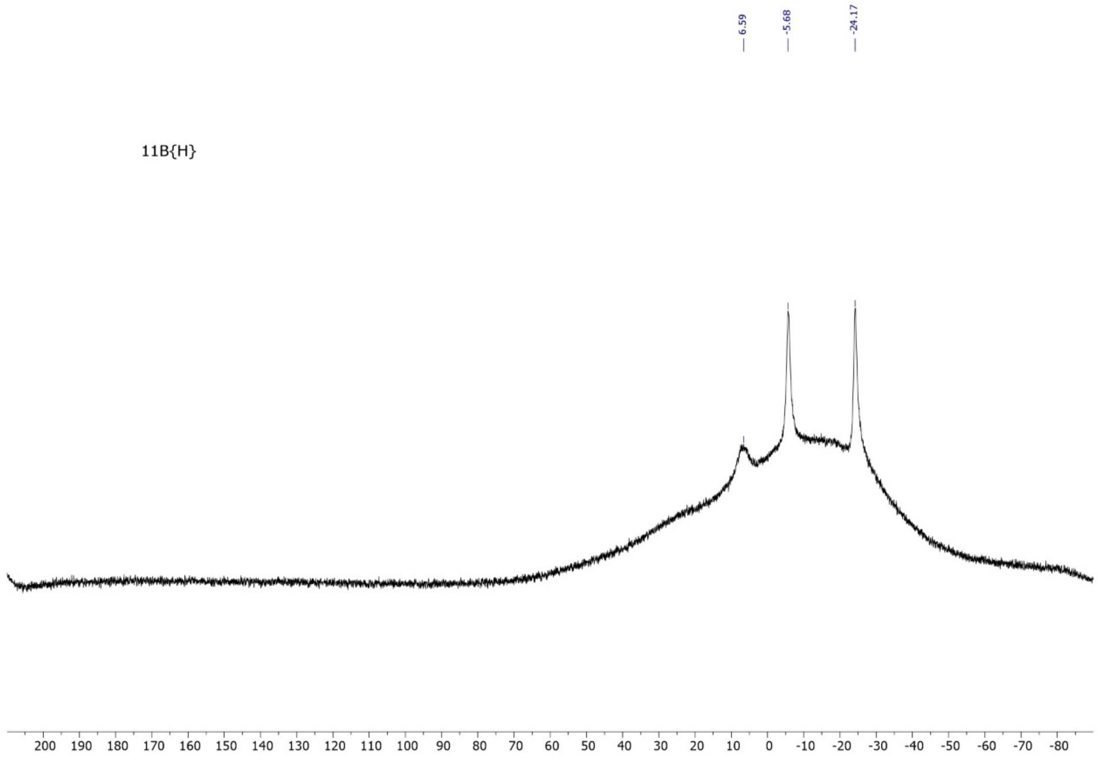

Figure S30. ^{11}B{^{1}H} NMR spectrum of **6**.

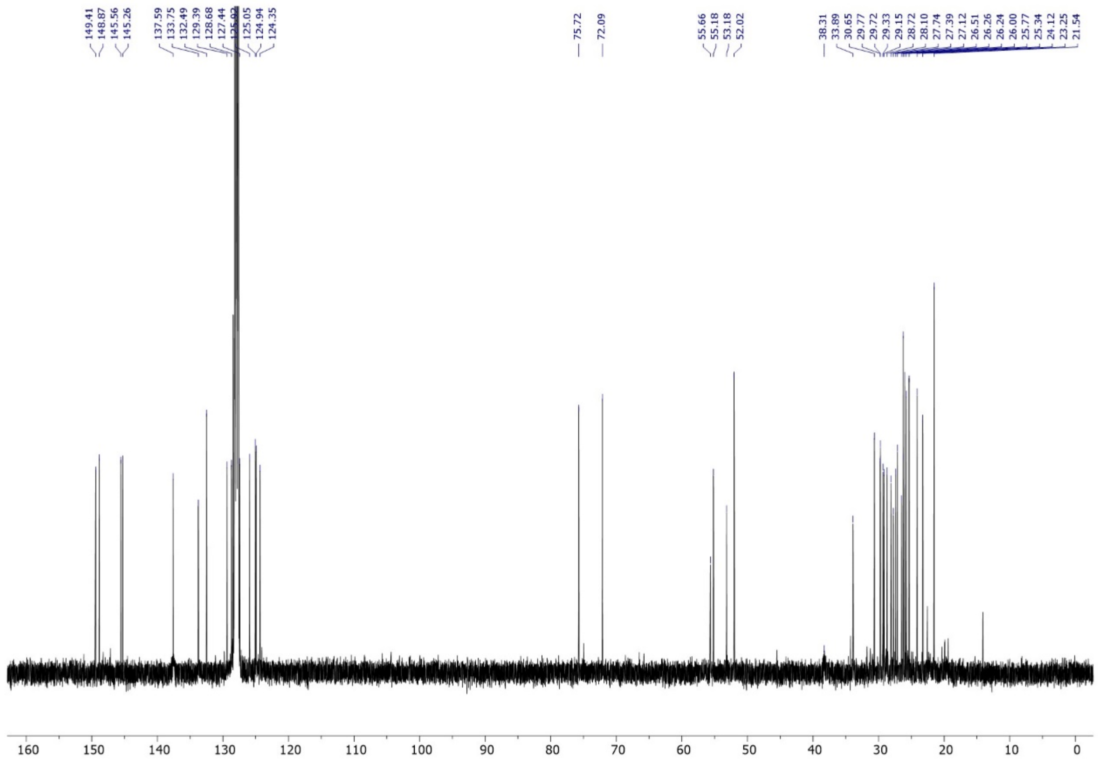

Figure S31. $^{13}C\{^{1}H\}$ NMR spectrum of **6**.

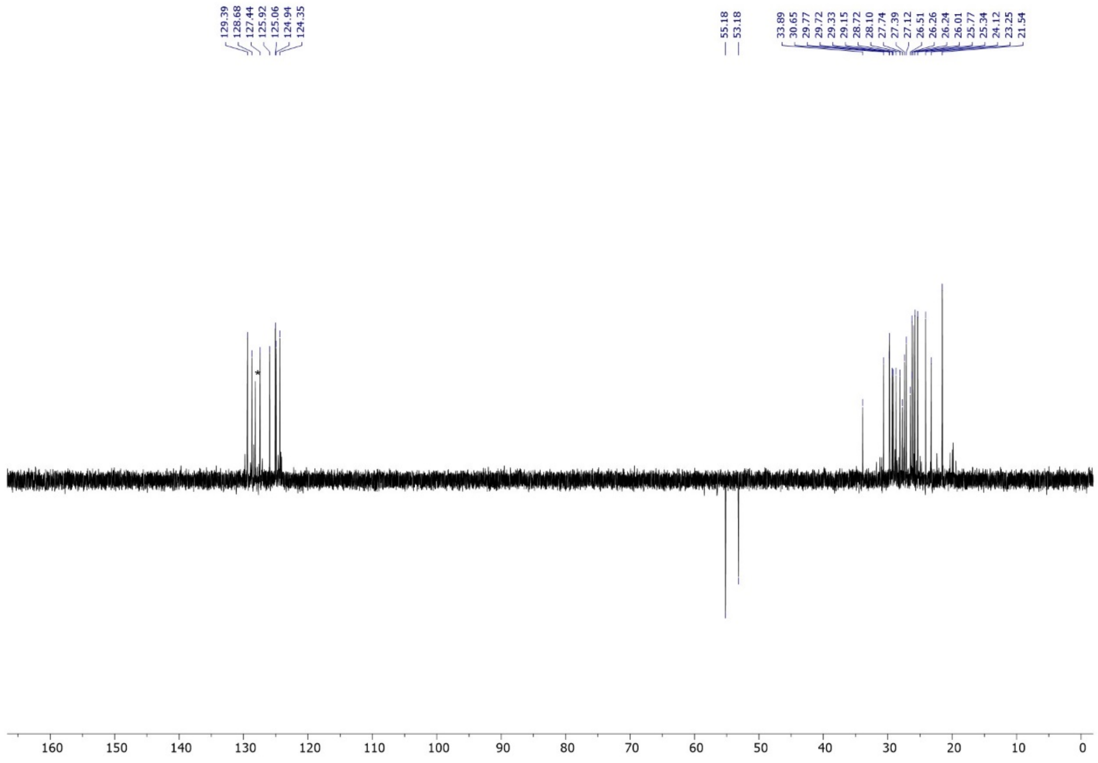

Figure S32. $^{13}C\{^{1}H\}$ NMR (DEPT 135) spectrum of **6** (*benzene).

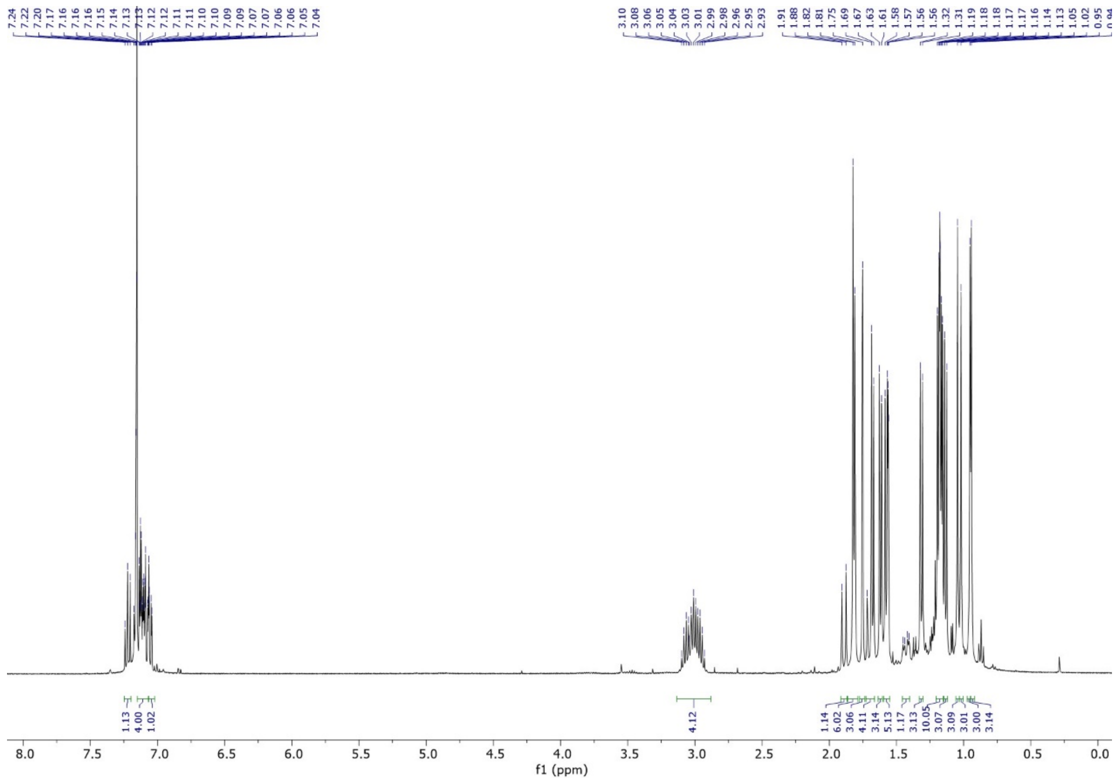

Figure S33. ^{1}H NMR spectrum of **7**.

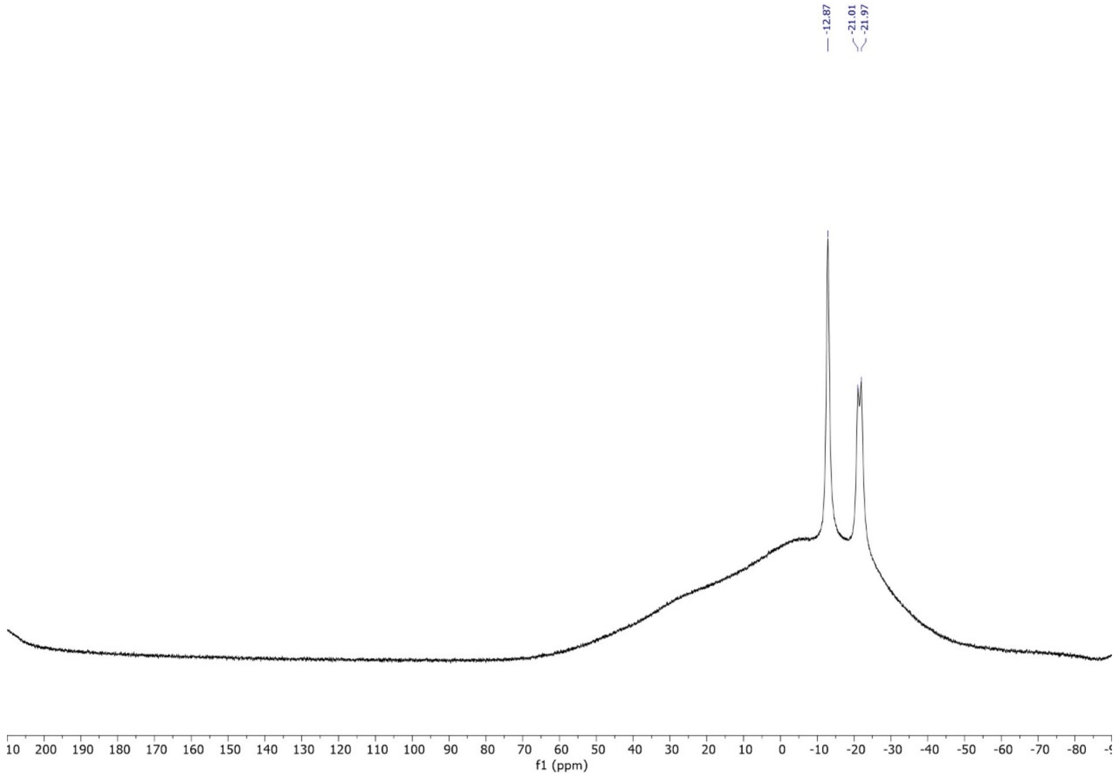

Figure S34. ^{11}B NMR spectrum of **7**.

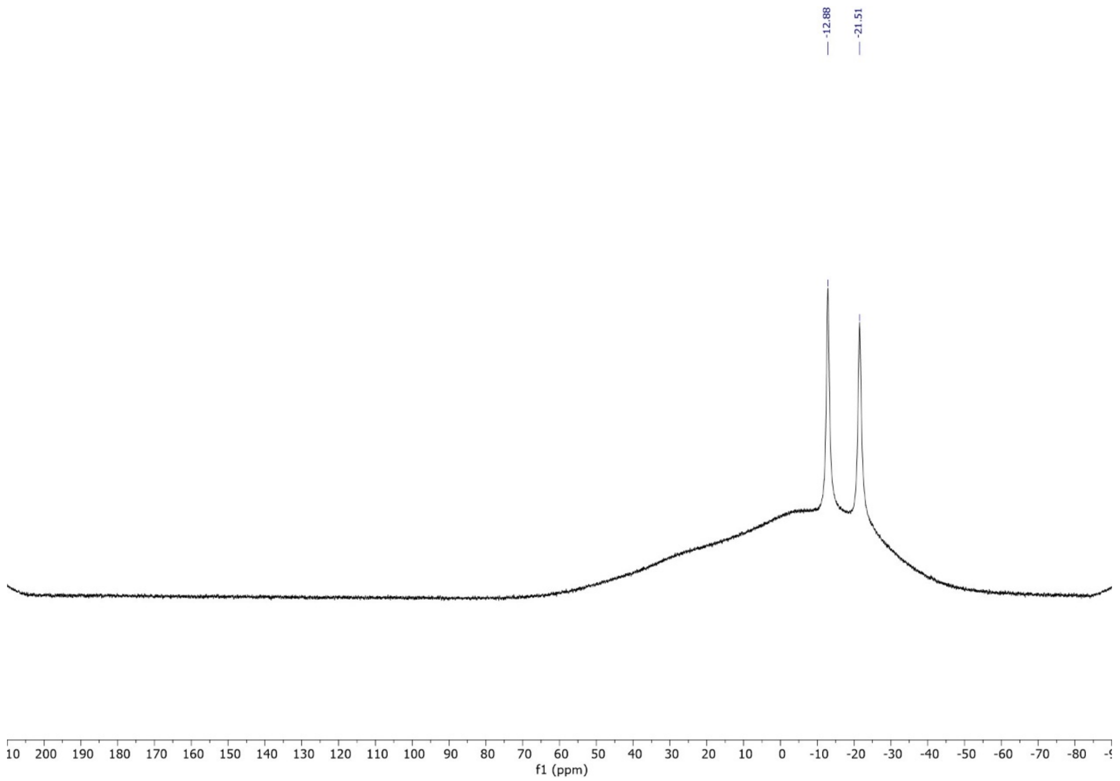

Figure S35. $^{11}B\{^{1}H\}$ NMR spectrum of **7**.

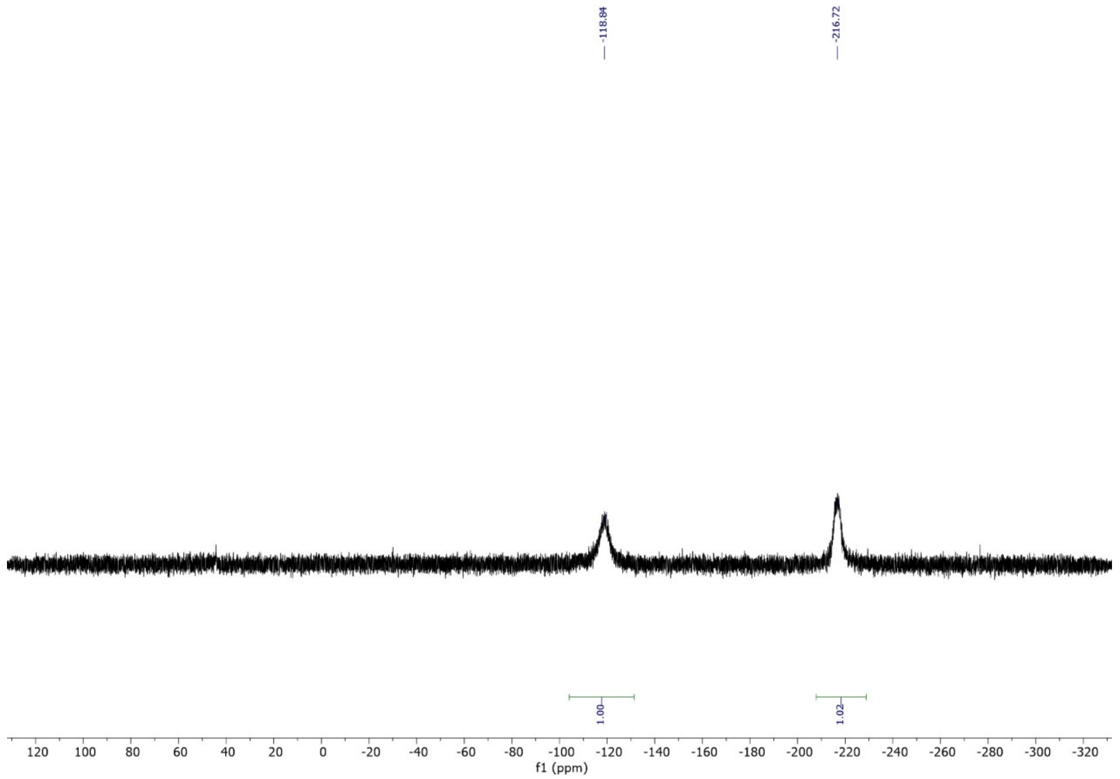

Figure S36. ^{31}P NMR spectrum of **7**.

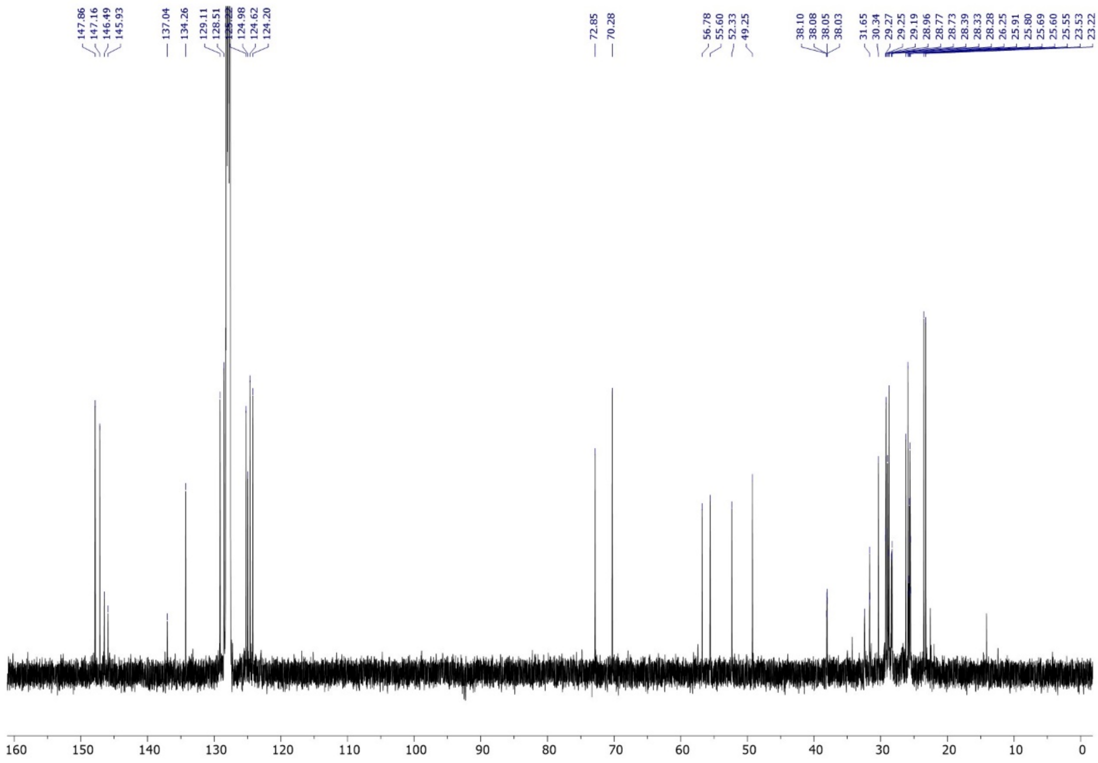

Figure S37. $^{13}C\{^{1}H\}$ NMR spectrum of **7**.

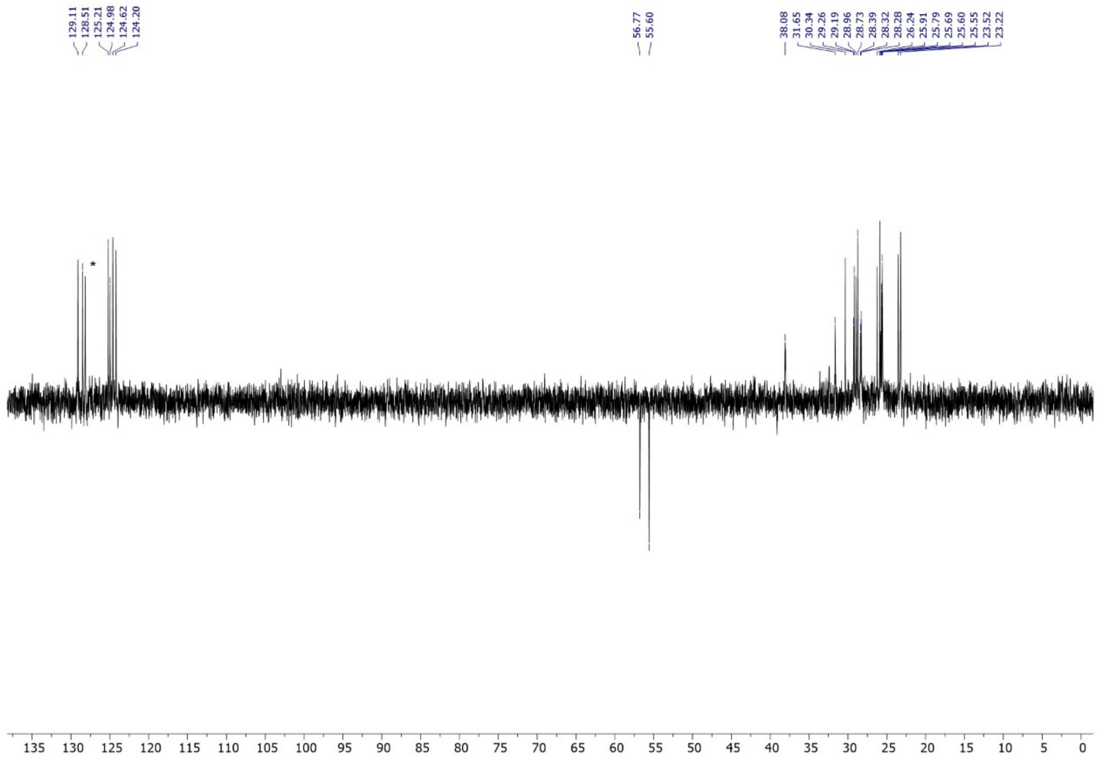

Figure S38. $^{13}C\{^{1}H\}$ NMR (DEPT 135) spectrum of **7** (*benzene).

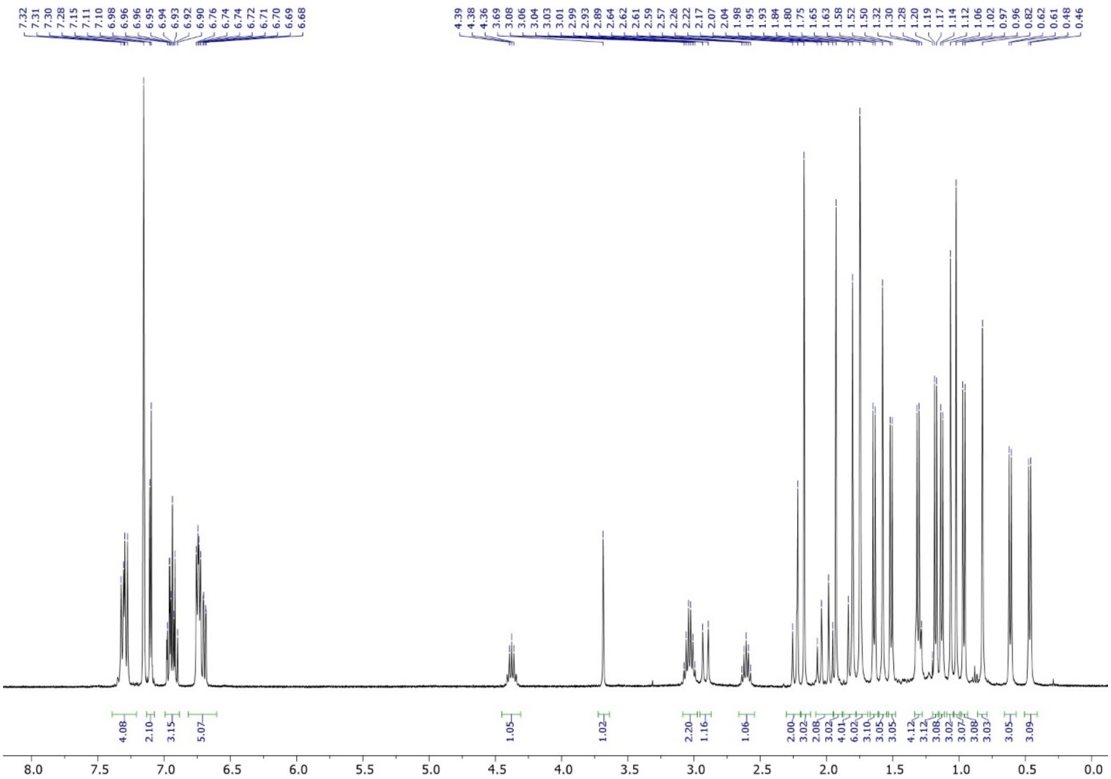

Figure S39. ^{1}H NMR spectrum of **8**.

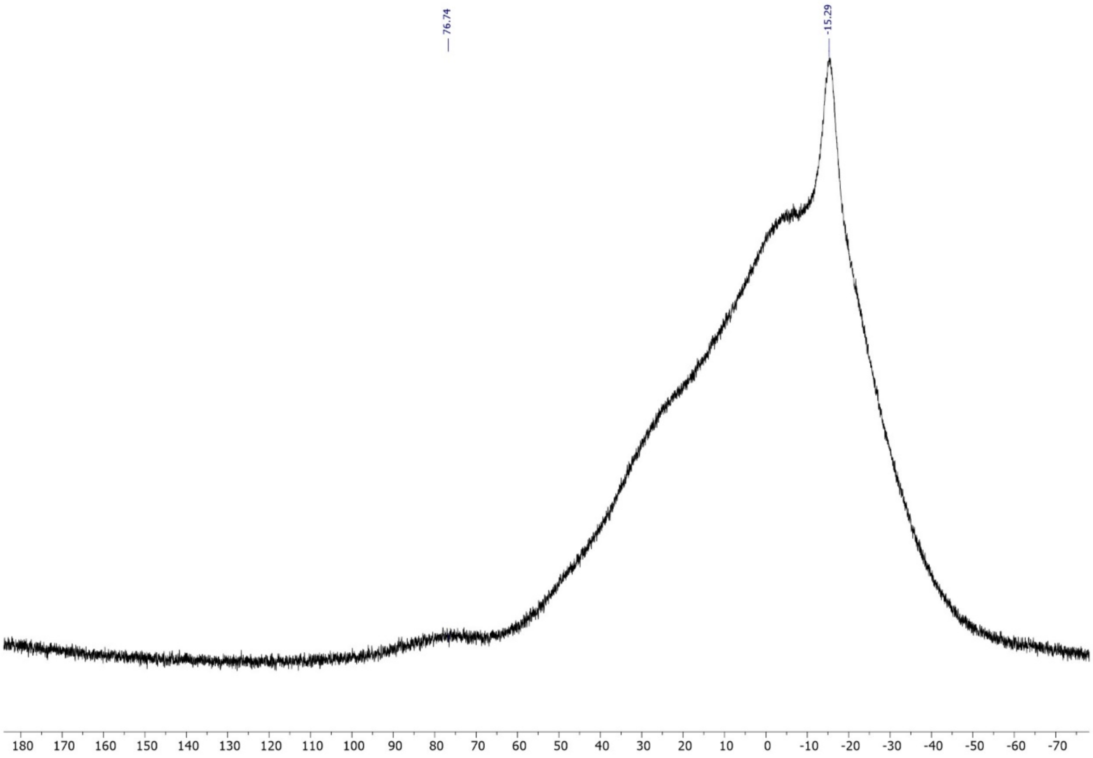

Figure S40. ^{11}B NMR spectrum of **8**.

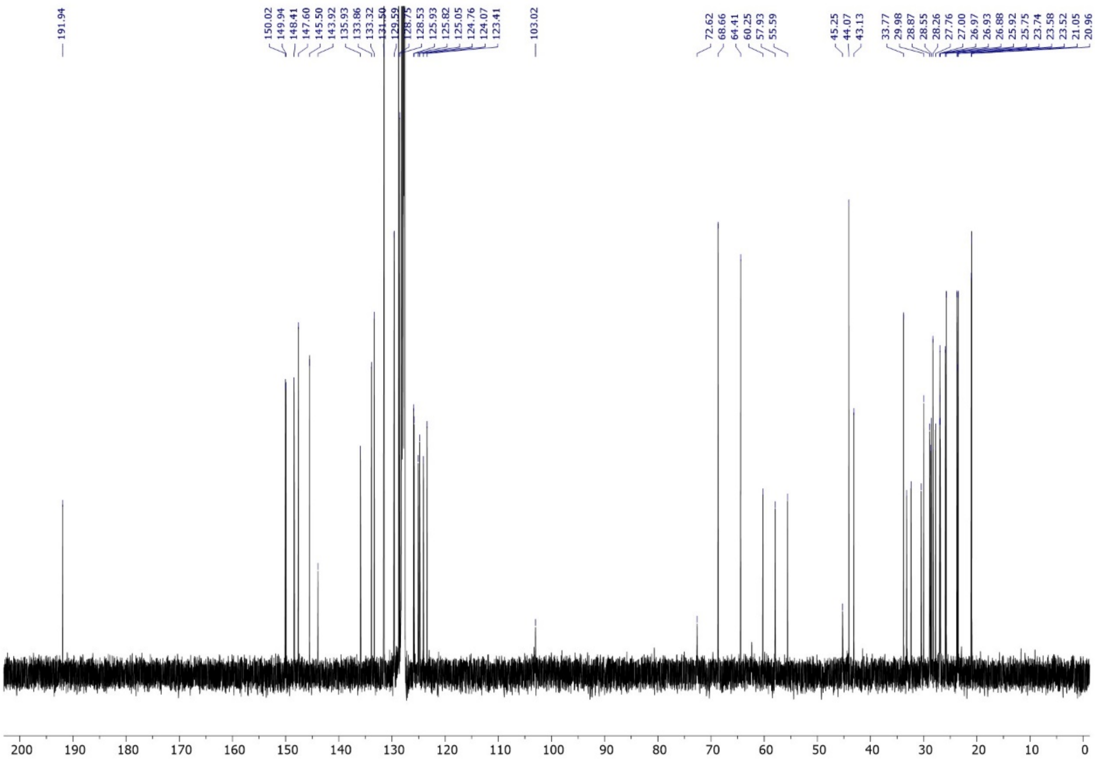

Figure S41. $^{13}C\{^1H\}$ NMR spectrum of **8**.

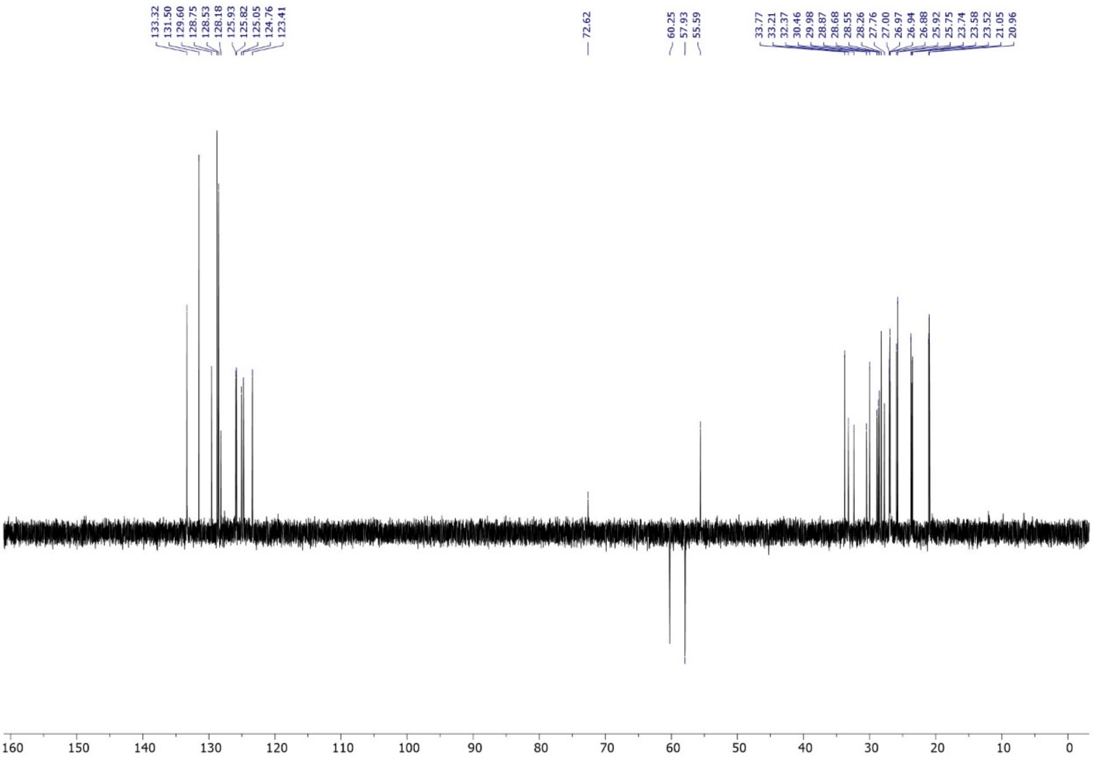

Figure S42. $^{13}C\{^1H\}$ NMR (DEPT 135) spectrum of **8**.

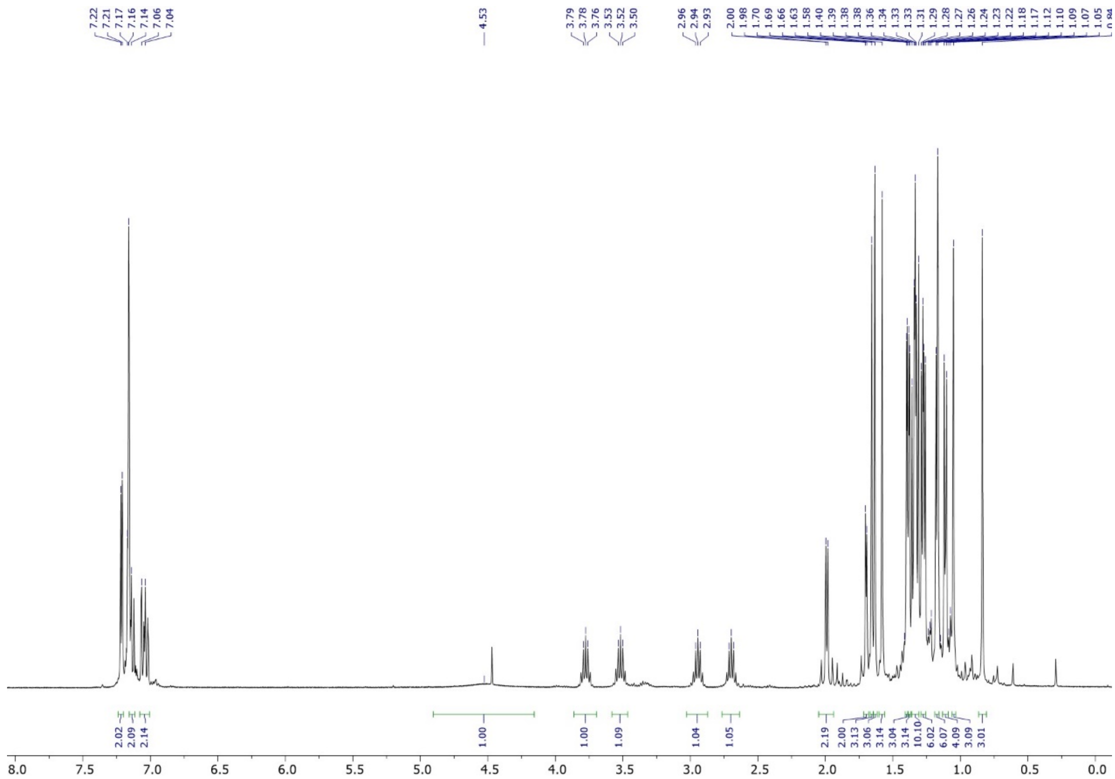

Figure S43. ^{1}H NMR spectrum of **10**.

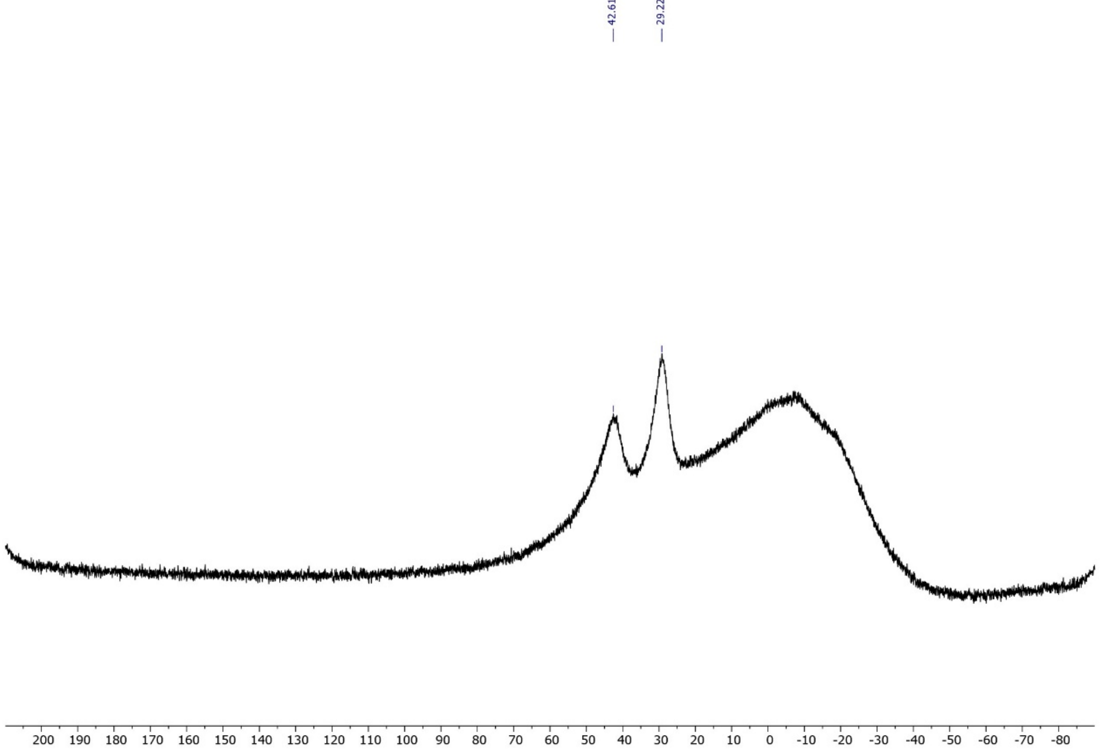

Figure S44. ^{11}B NMR spectrum of **10**.

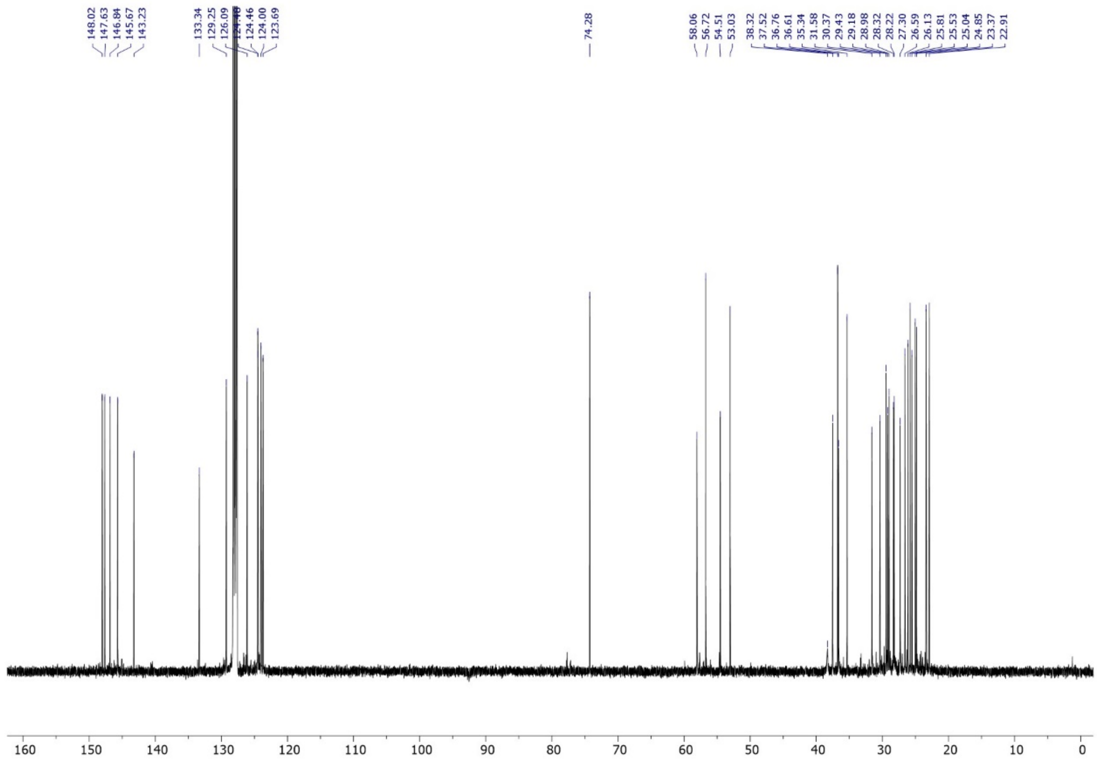

Figure S45. $^{13}C\{^{1}H\}$ NMR spectrum of **10**.

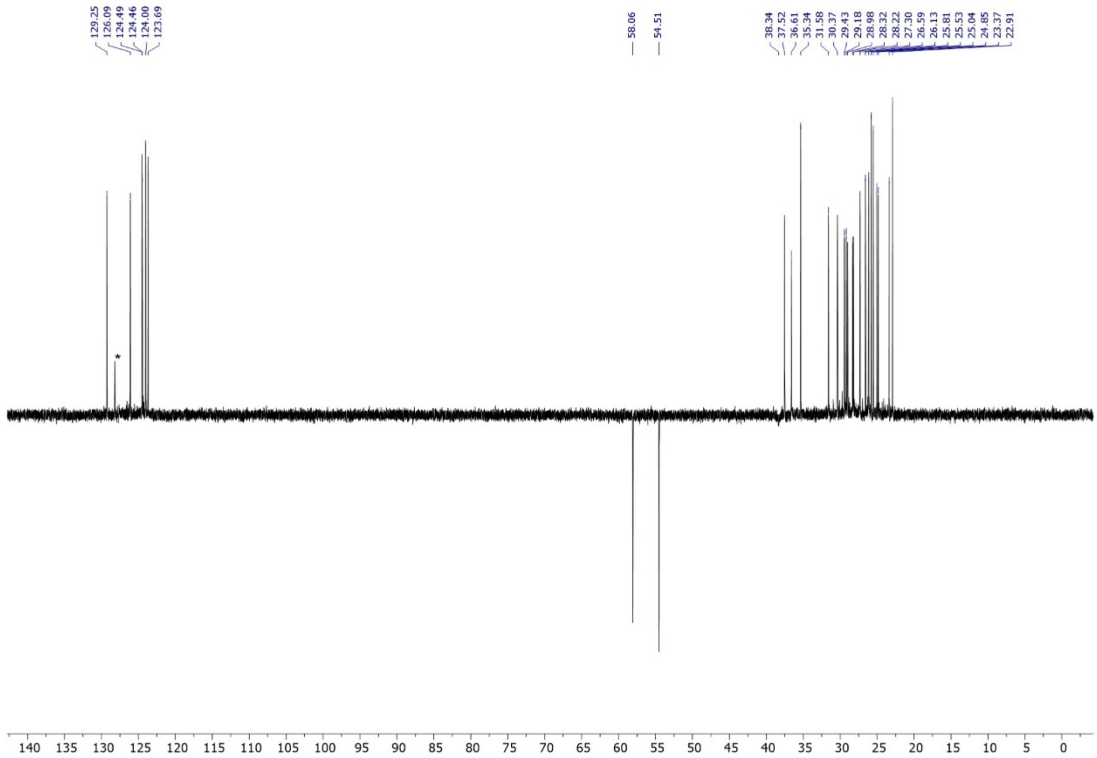

Figure S46. $^{13}C\{^{1}H\}$ NMR (DEPT 135) spectrum of **10** (*benzene).

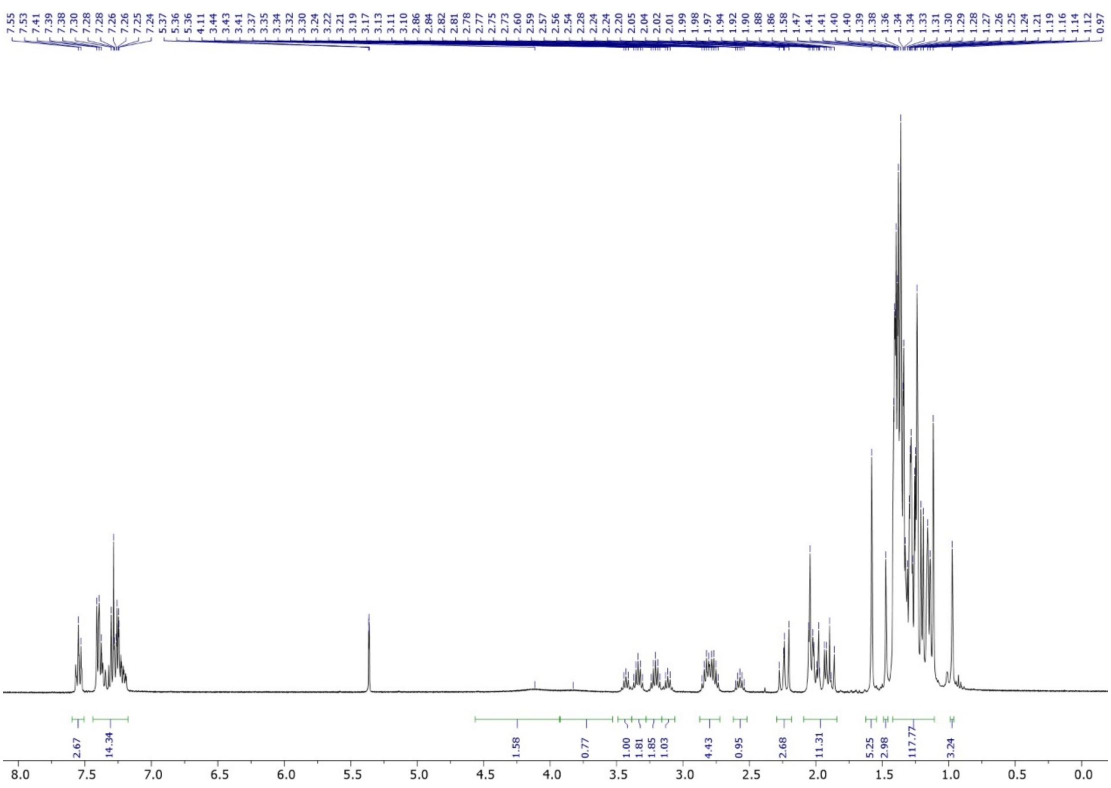

Figure S47. ^{1}H NMR spectrum of **11** and **11’**.

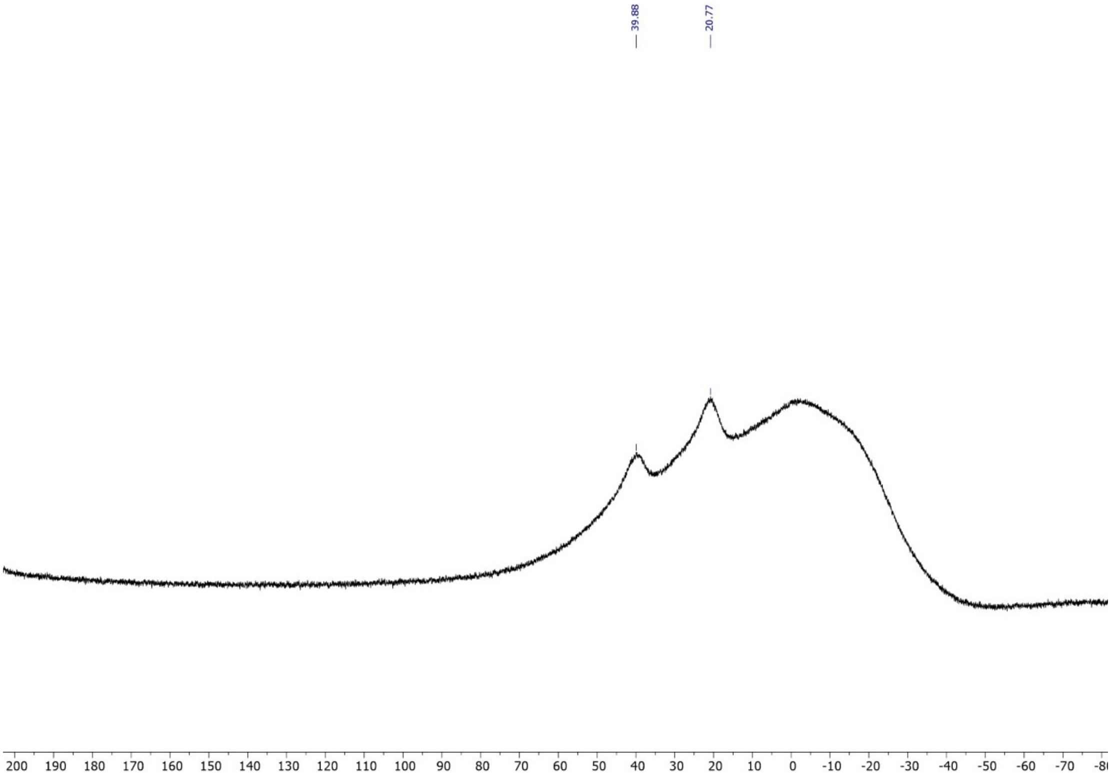

Figure S48. ^{11}B NMR spectrum of **11** and **11’**.

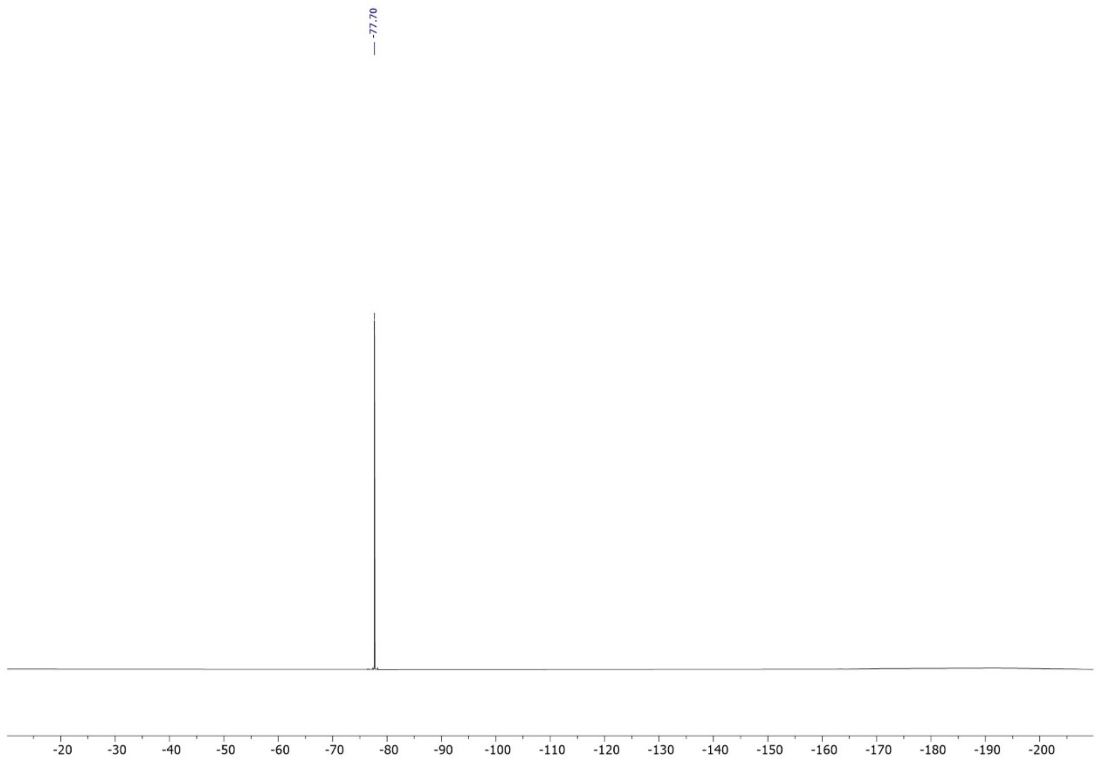

Figure S49. ^{19}F NMR spectrum of **11** and **11'**.

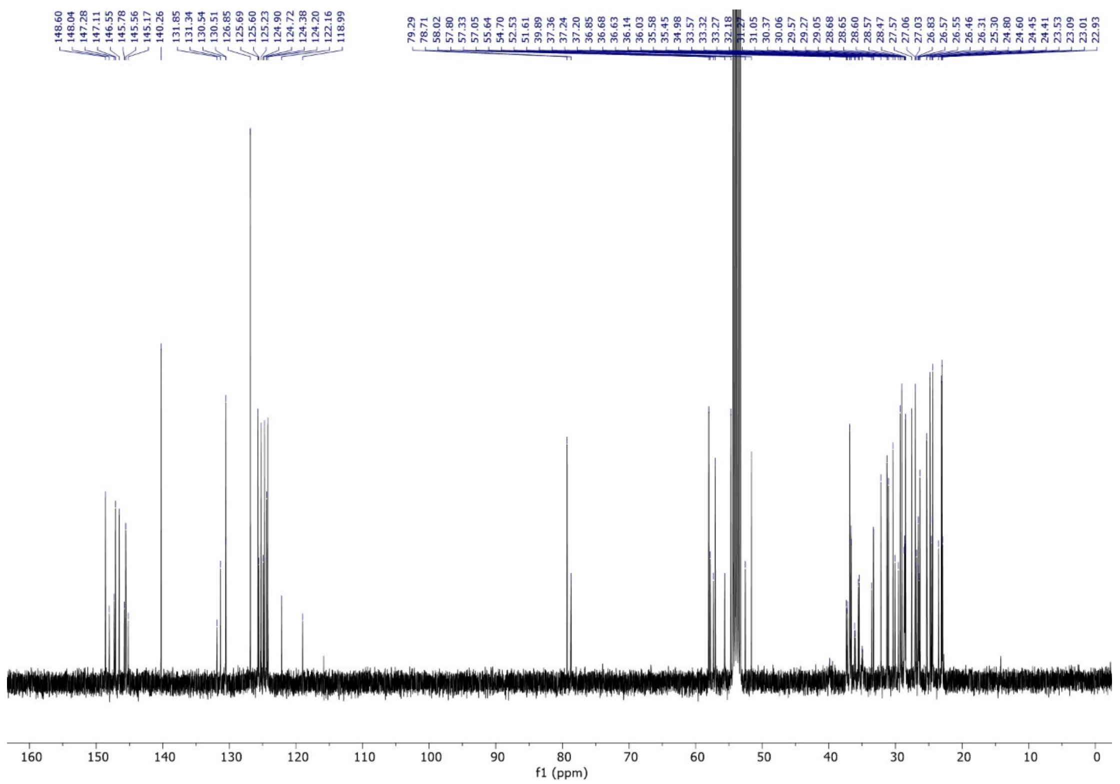

Figure S50. $^{13}C\{^1H\}$ NMR spectrum of **11** and **11'**.

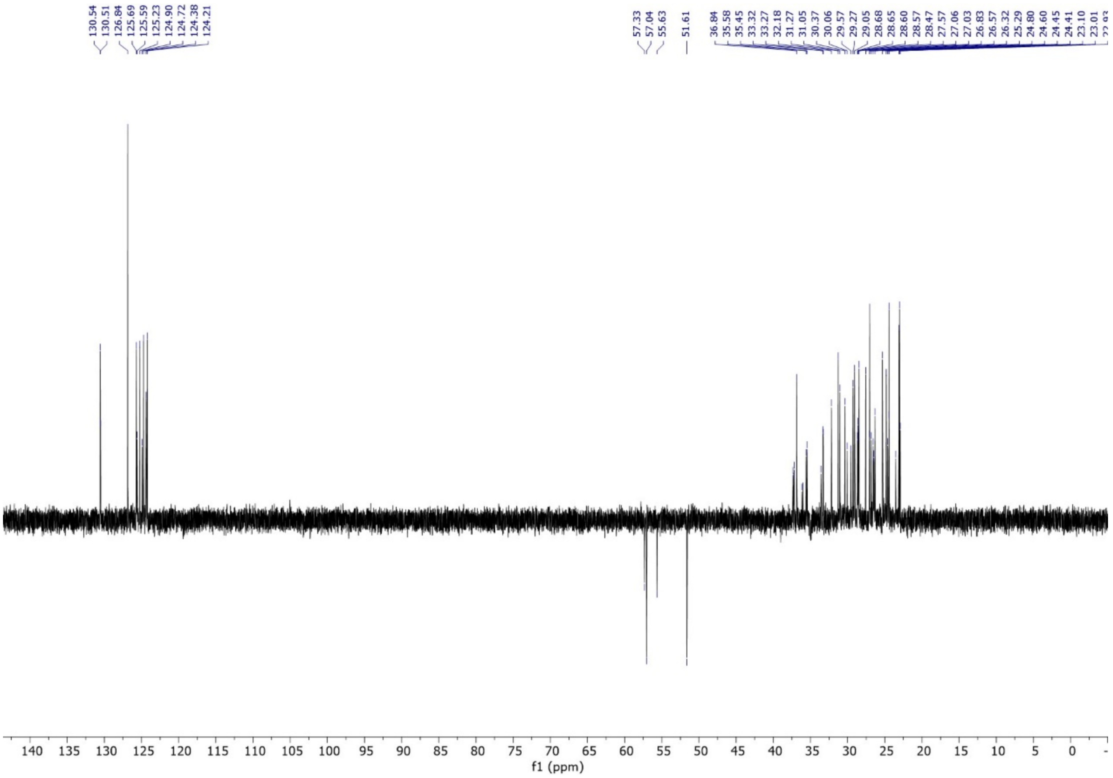

Figure S51. $^{13}C\{^{1}H\}$ NMR (DEPT 135) spectrum of **11** and **11'**.

2. Crystallographic details

The crystal data of **1-4** and **6-9** were collected on a Bruker D8 Quest diffractometer with a CMOS area detector and multi-layer mirror monochromated $Mo_{K\alpha}$ radiation. The crystal data of **10** and **11'** were collected on a Bruker X8-APEX II diffractometer with a CCD area detector and multi-layer mirror monochromated $Mo_{K\alpha}$ radiation. The crystal data of **11** was collected on a Rigaku XtaLAB Synergy-R diffractometer with a HPA area detector and multi-layer mirror monochromated $Cu_{K\alpha}$ radiation. The structure was solved using the intrinsic phasing method,[3] refined with the ShelXL program[4] and expanded using Fourier techniques. All non-hydrogen atoms were refined anisotropically. Hydrogen atoms were included in structure factor calculations. All hydrogen atoms were assigned to idealized positions, except those bound to boron, which were refined freely.

CCDC: 2112891-2112901 contains the supplementary crystallographic data for this paper. The data can be obtained free of charge from the Cambridge Crystallography Data Center via www.ccdc.cam.ac.uk/data_request/cif.

Crystal data for 1: $C_{40}H_{62}B_2Br_2N_2$, M_r = 752.35, orange needle, 0.276×0.13×0.053 mm^3, monoclinic space group $P2_1/c$, a = 18.291(5) Å, b = 10.672(4) Å, c = 20.039(9) Å, β = 97.436(15)°, V = 3879(2) Å^3, Z = 4, ρ_{calcd} = 1.288 g·cm^{-3}, μ = 2.119 mm^{-1}, F(000) = 1584, T = 100(2) K, R_1 = 0.0654, wR_2 = 0.1084, 8380 independent reflections [2θ≤54.34°] and 434 parameters.

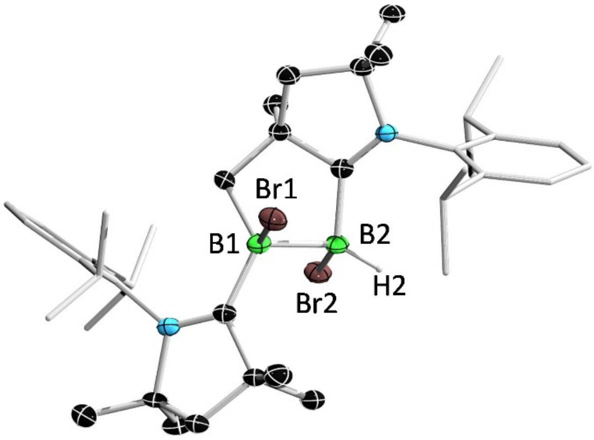

Crystal data for 2: $C_{40}H_{62}B_2N_2$, M_r = 592.53, blue block, 0.863×0.605×0.54 mm^3, monoclinic space group $C2/c$, a = 31.0908(10) Å, b = 14.3209(4) Å, c = 18.4709(5) Å, β = 112.7460(10)°, V = 7584.5(4) Å^3, Z = 8, ρ_{calcd} = 1.038 g·cm^{-3}, μ = 0.058 mm^{-1}, F(000) = 2608, T = 100(2) K, R_1 = 0.0436, wR_2 = 0.1061, 8072 independent reflections [2θ≤53.564°] and 416 parameters.

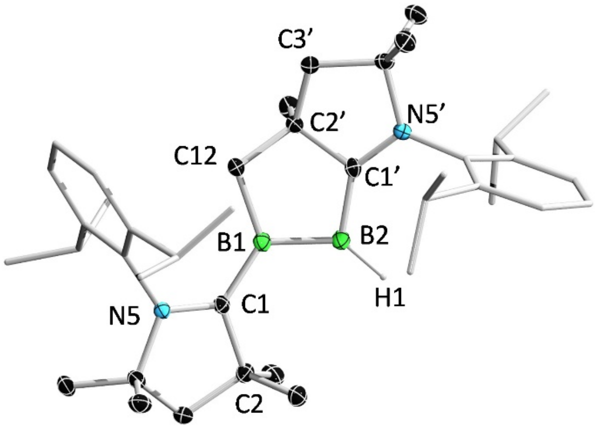

Refinement details for 3: The unit cell contains solvent molecules which have been treated as a diffuse contribution to the overall scattering without specific atom positions by SQUEEZE/PLATON. The displacement parameters of carbon atoms in the disordered cyclohexyl groups were restrained to the same value with similarity restraint SIMU. The U_{ii} displacement parameters of these atoms were restrained with ISOR keyword to approximate isotropic behavior as well. The distances between atoms C1_6 P1_1 and C1_16 P1_1 were restrained during refinement to the same value. The 1–2 and 1–3 distances of carbon atoms in the disordered cyclohexyl groups were restrained to the same values with SAME.

Crystal data for 3: $C_{58}H_{95}AuB_2BrN_2P$, M_r = 1149.82, red block, 0.267×0.153×0.135 mm^3, triclinic space group $P\bar{1}$, a = 14.4035(9) Å, b = 15.3336(9) Å, c = 17.3762(10) Å, α = 72.749(2)°, β = 86.460(2)°,

γ = 63.586(2)°, V = 3271.3(3) Å^3, Z = 2, ρ_{calcd} = 1.167 g·cm^{-3}, μ = 2.916 mm^{-1}, F(000) = 1192, T = 100(2) K, R_1 = 0.0228, wR_2 = 0.0482, 11548 independent reflections [2θ≤50.054°] and 660 parameters.

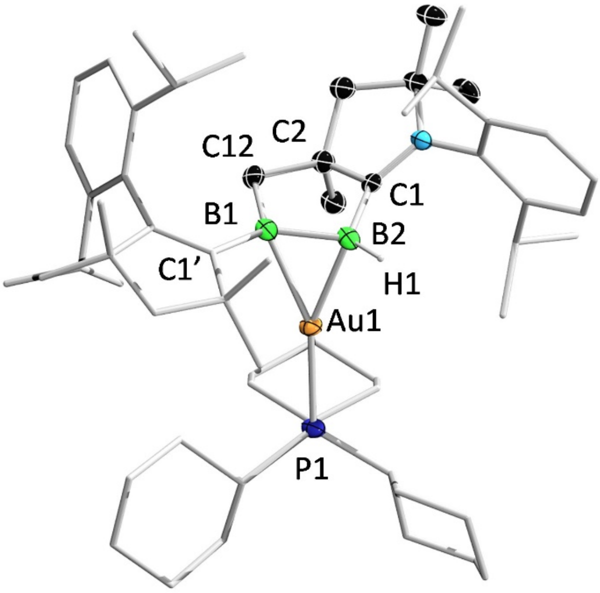

Refinement details for 4: The displacement parameters of atoms Cl1_3 and Br1_3 were constrained to the same value with EADP keyword.

Crystal data for 4: $C_{46}H_{68}AuB_2Br_{0.37}Cl_{0.63}N_2$, M_r = 919.57, red block, 0.164×0.159×0.09 mm^3, monoclinic space group $P2_1/n$, a = 20.034(4) Å, b = 9.685(2) Å, c = 23.325(7) Å, β = 108.88(2)°, V = 4282.5(18) Å^3, Z = 4, ρ_{calcd} = 1.426 g·cm^{-3}, μ = 3.854 mm^{-1}, F(000) = 1883, T = 100(2) K, R_1 = 0.0295, wR_2 = 0.0521, 9126 independent reflections [2θ≤53.542°] and 492 parameters.

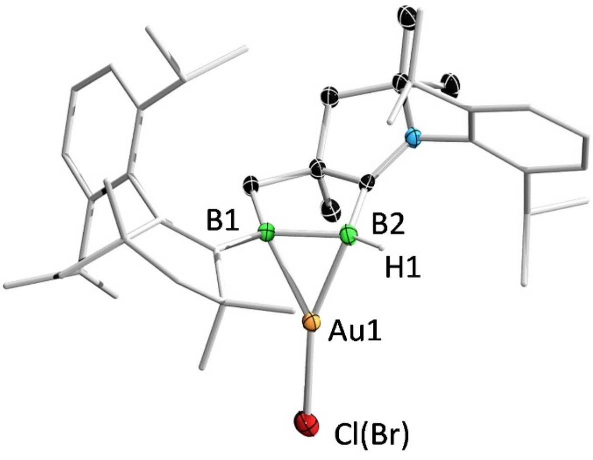

Refinement details for 6: The similarity restraint on displacement amplitudes (SIMU) was employed for the atomic displacement parameters of disordered benzene fragments. The bond distances of atoms were restrained to be similar by means of the similar restraint (SAME).

Crystal data for 6: $C_{62}H_{89}B_3N_2$, M_r = 894.78, red block, 0.274×0.239×0.236 mm^3, triclinic space group $P\overline{1}$, a = 11.0422(9) Å, b = 14.7580(9) Å, c = 16.8203(12) Å, α = 87.944(3)°, β = 89.704(3)°, γ = 86.727(2)°, V = 2734.8(3) Å^3, Z = 2, ρ_{calcd} = 1.087 g·cm^{-3}, μ = 0.061 mm^{-1}, F(000) = 980, T = 100(2) K, R_1 = 0.0508, wR_2 = 0.1116, 9643 independent reflections [2θ≤50.054°] and 745 parameters.

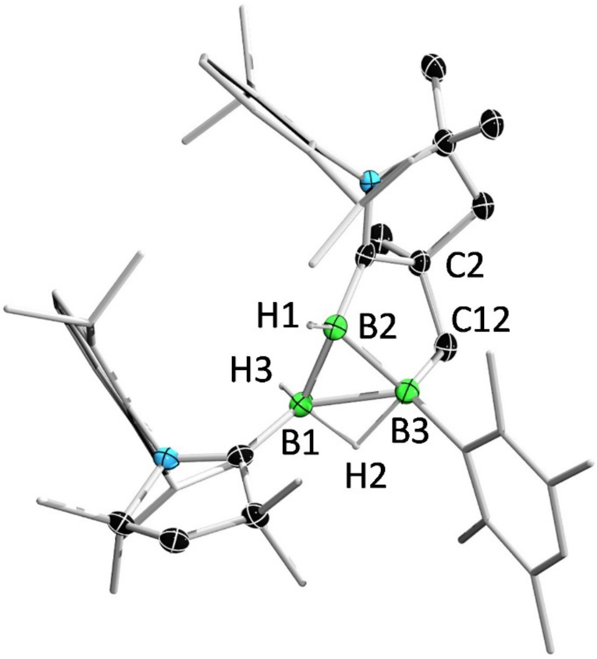

Crystal data for 7: $C_{40}H_{62}B_2N_2P_2$, M_r = 654.47, red plate, 0.253×0.146×0.088 mm^3, triclinic space group $P\overline{1}$, a = 9.0344(4) Å, b = 11.9089(4) Å, c = 19.1009(8) Å, α = 78.2280(10)°, β = 83.769(2)°, γ = 71.5850(10)°, V = 1906.67(13) Å^3, Z = 2, ρ_{calcd} = 1.140 g·cm^{-3}, μ = 0.144 mm^{-1}, F(000) = 712, T = 100(2) K, R_1 = 0.0564, wR_2 = 0.1046, 8125 independent reflections [2θ≤53.564°] and 434 parameters.

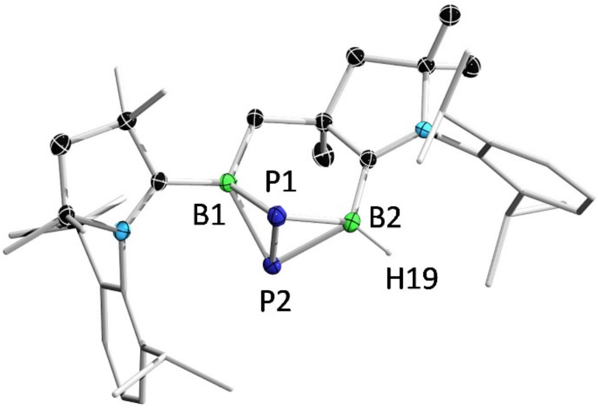

Refinement details for 8: The similarity restraint on displacement amplitudes (SIMU) was employed for the atomic displacement parameters of disordered benzene fragments. The bond distances of atoms were restrained to be similar by means of the similar restraint (SAME).

Crystal data for 8: $C_{67}H_{87}B_2N_2$, M_r = 942.00, colorless plate, 0.411×0.198×0.082 mm^3, triclinic space group $P\bar{1}$, a = 11.9416(10) Å, b = 16.6268(14) Å, c = 17.3846(15) Å, α = 61.967(3)°, β = 86.652(3)°, γ = 70.581(3)°, V = 2853.5(4) Å^3, Z = 2, ρ_{calcd} = 1.096 g·cm^{-3}, μ = 0.061 mm^{-1}, F(000) = 1026, T = 100(2) K, R_1 = 0.0829, wR_2 = 0.1519, 12161 independent reflections [2θ≤53.694°] and 658 parameters.

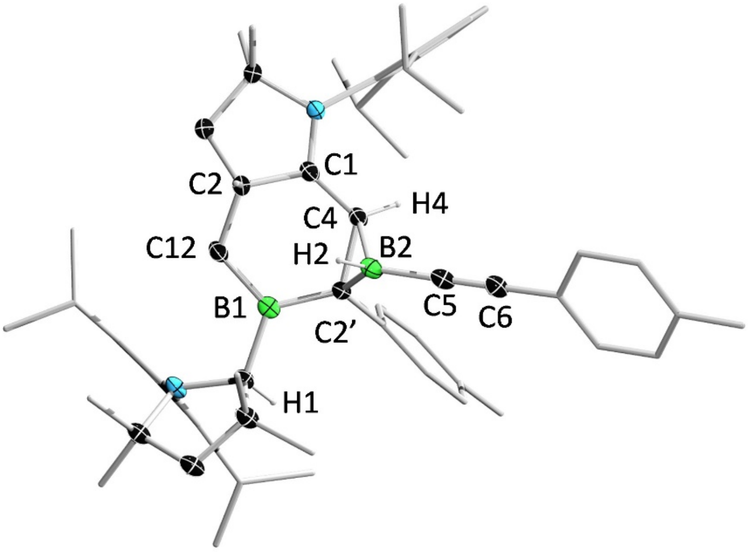

Refinement details for 9: The soft restraint (ISOR) and similarity restraint on displacement amplitudes (SIMU) were employed for the atomic displacement parameters of disordered 'ME' fragments. The bond distances of atoms were restrained to be similar by means of the similarity restraint (SAME). The distances between N11 and C1, as well as the C14 and C4 atoms in disordered 'ME' groups were restrained during refinement to the same value with SADI restraint, respectively. The disordered benzene solvent molecule was treated with SQUEEZE. The following reflections were removed by 'OMIT': 1 −2 3, 1 −1 2, 1 −1 3, 2 1 0, 0 2 0, 2 −2 2, 1 1 2, −1 3 3, 2 0 1, −3 3 3, −2 0 3, 2 0 0, 1 0 3, 0 −1 2, 2 −1 2, −1 2 1, 0 −4 3, 2 2 4, −3 1 0, 0 −1 4, 1 −1 1, 2 4 1, −2 −1 1.

Crystal data for 9: $C_{96}H_{134}B_4HgN_4$, M_r = 1587.89, orange plate, 0.274×0.199×0.116 mm^3, triclinic space group $P\bar{1}$, a = 10.6266(7) Å, b = 13.2239(9) Å, c = 18.0566(12) Å, α = 105.989(2)°, β = 102.836(2)°, γ = 97.860(2)°, V = 2324.6(3) Å^3, Z = 1, ρ_{calcd} = 1.134 g·cm^{-3}, μ = 1.699 mm^{-1}, F(000) = 838, T = 100(2) K, R_1 = 0.0416, wR_2 = 0.1198, 8202 independent reflections [2θ≤50.052°] and 525 parameters.

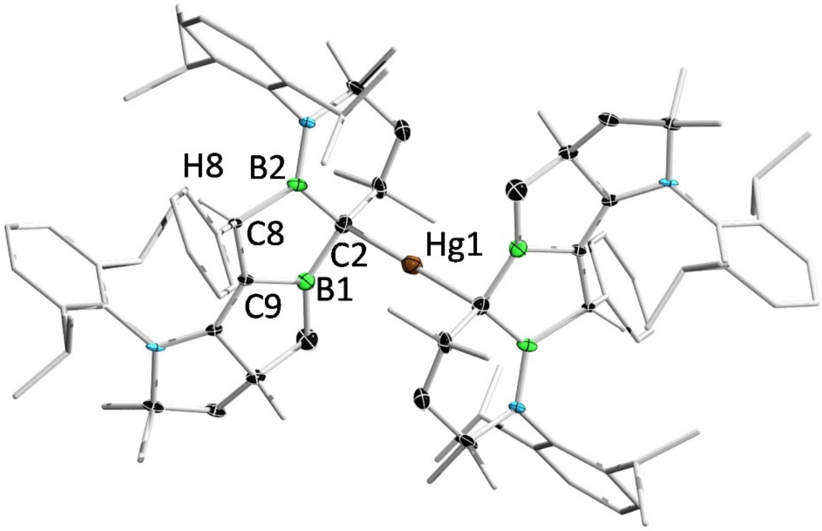

Crystal data for 10: $C_{40}H_{62}B_2N_2$, M_r = 592.53, orange block, 0.404×0.295×0.286 mm^3, monoclinic space group $P2_1/n$, a = 10.6576(3) Å, b = 24.6496(8) Å, c = 15.2266(5) Å, β = 108.275(2)°, V = 3798.4(2) Å^3, Z = 4, ρ_{calcd} = 1.036 g·cm^{-3}, μ = 0.058 mm^{-1}, F(000) = 1304, T = 99(2) K, R_1 = 0.0774, wR_2 = 0.1225, 8127 independent reflections [2θ≤53.646°] and 416 parameters.

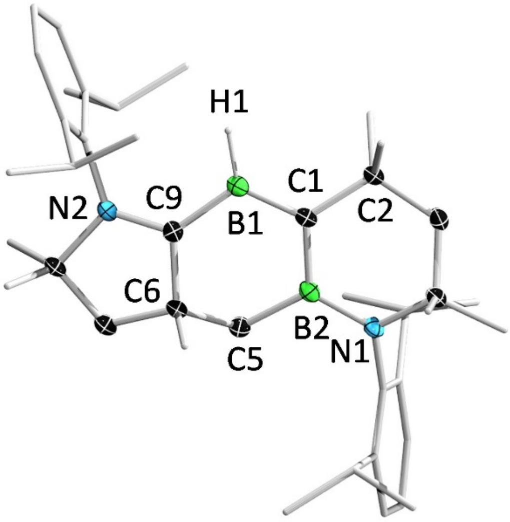

Refinement details for 11: The displacement parameters of atoms C2 and C7 of residue 2 (CAAC) were constrained to the same value with EADP keyword. The same also applies for atoms C4 and C9 of residue 2 (CAAC) and atoms C6 and C11 of residue 2 (CAAC). The coordinates of atoms C2 and C7 of residue 2 (CAAC) were constrained to the same value. The same also applies for atoms C4 and C9 of residue 2 (CAAC). The atomic displacement parameters of atoms C2 to C11 of residue 2 (CAAC) were restrained with the RIGU keyword in ShelXL input ('enhanced rigid bond' restraint for all bonds in the connectivity list). The same also applies for atoms S1 to F8 of residues 6 and 16 (OTf). The displacement parameters of atoms C2 to C11 of residue 2 (CAAC) were restrained to the same value with similarity restraint SIMU. The same also applies for atoms S1 to F8 of residues 6 and 16 (OTf). The 1–2 and 1–3 distances in residues 6 and 16 (OTf) were restrained to the same values with SAME due to disorder of the counterion.

Crystal data for 11: $C_{47}H_{68}AgB_2F_3N_2O_3S$, M_r = 927.58, clear colorless block, 0.119×0.070×0.028 mm^3, space group $P2_1/c$, a = 24.2041(2) Å, b = 9.40910(10) Å, c = 20.36720(10) Å, α = 90°, β = 91.2020(10)°, γ = 90°, V = 4637.38(7) Å^3, Z = 4, ρ_{calcd} = 1.329 g·cm^{-3}, μ = 4.336 mm^{-1}, F(000) = 1952, T = 100.00(10) K, R_1 = 0.0638, wR_2 = 0.1214, 9452 independent reflections [2θ≤150.994°] and 647 parameters.

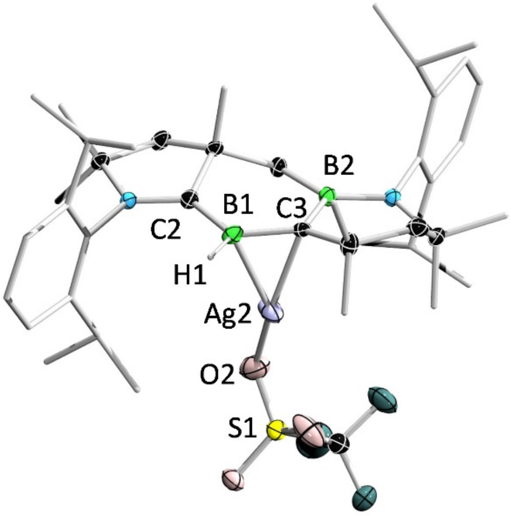

Crystal data for 11': $C_{82}H_{124}Ag_2B_4F_6N_4O_6S_2$, M_r = 1698.94, orange block, 0.452×0.262×0.214 mm^3, triclinic space group $P\bar{1}$, a = 10.5797(6) Å, b = 12.6112(7) Å, c = 16.6585(9) Å, α = 70.490(2)°, β = 86.906(2)°, γ = 79.663(2)°, V = 2061.0(2) Å^3, Z = 1, ρ_{calcd} = 1.369 g·cm^{-3}, μ = 0.593 mm^{-1}, F(000) = 892, T = 100(2) K, R_1 = 0.0417, wR_2 = 0.0997, 8725 independent reflections [2θ≤53.626°] and 493 parameters.

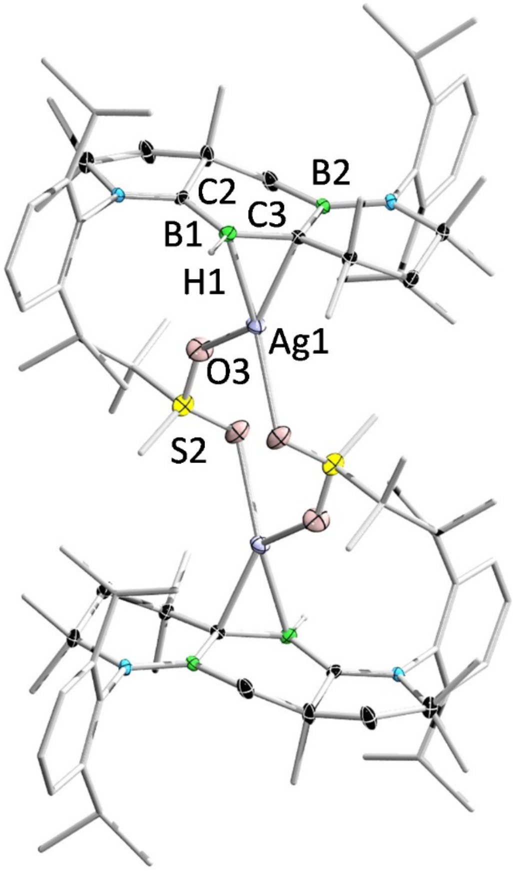

3. Computational details

Gaussian 09E and Gaunssian 16B were used for the density functional theory (DFT) calculations.[5] NBO analyses were performed using the program version NBO 7.[6] All calculations such as geometry optimization, frequency calculations, natural bond order (NBO) analysis and TD-DFT calculations of **2** and simplified model compounds **8'** and **10'** were performed at the B3LYP/6-311G* level of theory. For simplification, the 2,6-diisopropylphenyl groups of compounds **8** and **10** were replaced with 2,6-dimethylphenyl groups. Mayer bond order (MBO),[7] fuzzy bond order (FBO)[8] and quantum theory of atoms in molecules (QTAIM)[9] studies of **8'** were achieved with Multiwfn. Intrinsic bond orbital (IBO)[10] calculations were carried out with IBOview.

Table S1: Optimized structures of a) **opt-2**, b) **opt-8'** c) and **10'** (atom, x-, y-, z- positions in Å).

a) opt-2

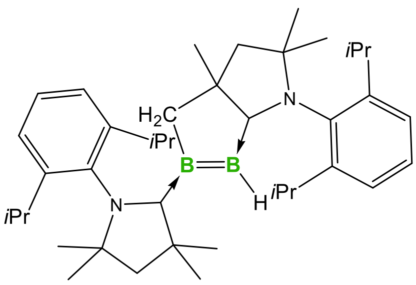

N	-3.137014	0.246602	0.851798
B	-0.626592	-0.441234	0.514612
N	2.903848	-0.377250	-0.707890
B	0.905967	-0.348723	1.056027
H	1.462559	0.030807	2.051538
C	-1.876752	0.022080	1.305894
C	-1.862626	0.301839	2.820907
C	-4.160629	-2.431275	0.090956
H	-3.917915	-2.067287	1.089144
C	-3.534987	0.016148	-0.524139
C	-4.033397	-1.259484	-0.884142
C	-4.112571	0.775156	1.889780
C	3.071427	-0.714163	-2.181568
C	3.944880	0.239623	0.084805
C	1.688631	-0.683331	-0.219537
C	-3.362853	0.425383	3.182852
H	-3.724771	-0.535577	3.561112
H	-3.541921	1.164587	3.967245
C	3.609684	0.474283	-2.985309
H	2.946173	1.335999	-2.930633
H	3.688207	0.185156	-4.036934
H	4.604851	0.776297	-2.650788
C	4.045121	1.648137	0.151428
C	-0.579639	-0.923016	-1.052668
H	-1.296571	-1.693159	-1.352248
H	-0.763368	-0.092729	-1.743061
C	0.876066	-1.417463	-1.264970
C	-5.486944	0.100861	1.823924
H	-6.133298	0.547350	2.584148
H	-5.434098	-0.966454	2.029284
H	-5.969127	0.247988	0.854996
C	4.835332	-0.583094	0.812771
C	4.036922	-1.892851	-2.391677
H	5.035556	-1.651011	-2.021772
H	4.125088	-2.102736	-3.461091
H	3.702446	-2.806872	-1.903767
C	-3.397533	1.039944	-1.490019
C	-4.337855	2.291900	1.753846
H	-4.980173	2.628148	2.572095
H	-4.842430	2.540761	0.820483
H	-3.410620	2.860303	1.810214
C	1.606287	-1.048257	-2.577691
H	1.143280	-0.160509	-3.018494
H	1.570595	-1.838743	-3.331878
C	-4.407678	-1.473254	-2.213426

H	-4.785952	-2.446434	-2.507894
C	-1.092609	1.603243	3.142616
H	-1.482587	2.465040	2.596606
H	-0.041373	1.495917	2.884535
H	-1.168792	1.823185	4.213161
C	2.995712	2.594822	-0.425266
H	2.275663	2.000309	-0.988315
C	-1.235723	-0.852001	3.626793
H	-0.171877	-0.946622	3.422985
H	-1.709908	-1.806991	3.384178
H	-1.375351	-0.669017	4.698805
C	-5.592071	-3.002035	0.125112
H	-5.689390	-3.732715	0.933775
H	-5.840479	-3.519535	-0.805560
H	-6.345008	-2.226769	0.278274
C	5.883969	0.026360	1.508134
H	6.580035	-0.589530	2.067414
C	-3.791740	0.765855	-2.803913
H	-3.691416	1.538965	-3.558382
C	5.115485	2.202766	0.860586
H	5.213475	3.281890	0.914941
C	6.041740	1.404587	1.515466
H	6.867670	1.856961	2.055907
C	0.921188	-2.944505	-1.011312
H	1.936941	-3.344024	-0.963905
H	0.394820	-3.468796	-1.815705
H	0.432740	-3.180878	-0.064776
C	-2.821775	2.425686	-1.197296
H	-2.625254	2.491975	-0.129631
C	-4.294517	-0.473508	-3.168426
H	-4.590770	-0.662700	-4.195606
C	2.208700	3.279264	0.709594
H	2.858799	3.913307	1.320274
H	1.740491	2.541762	1.362084
H	1.419131	3.913600	0.294322
C	4.644318	-2.089686	0.960006
H	3.878752	-2.403889	0.252569
C	3.592030	3.655647	-1.368083
H	2.793196	4.242595	-1.831392
H	4.188931	3.212174	-2.167317
H	4.234837	4.357701	-0.829827
C	-3.161003	-3.561998	-0.215445
H	-2.130711	-3.219462	-0.130022
H	-3.302636	-3.964137	-1.223156
H	-3.300598	-4.386306	0.490890

C	-1.474146	2.668762	-1.903138
H	-1.128623	3.688057	-1.703934
H	-1.557317	2.555285	-2.988078
H	-0.708463	1.982084	-1.543036
C	4.112082	-2.423292	2.367949
H	4.835440	-2.147944	3.141599
H	3.919253	-3.497010	2.458685
H	3.180673	-1.892748	2.573038

C	-3.810191	3.547216	-1.574115
H	-4.806425	3.382566	-1.157931
H	-3.923003	3.634606	-2.658385
H	-3.447910	4.512437	-1.208108
C	5.916909	-2.898615	0.657033
H	6.705103	-2.713775	1.392115
H	6.325004	-2.665252	-0.329292
H	5.698272	-3.970292	0.684089

b) **opt-8’**

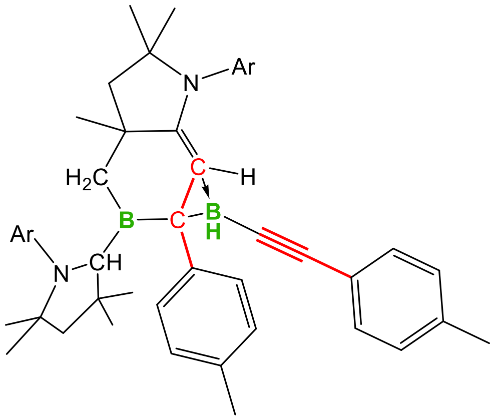

(Ar = 2,6-dimethylphenyl)

B	1.301414	-0.320720	-0.572206
C	-0.132339	-0.582322	-0.011147
B	-1.221365	-0.786435	-1.165390
H	-0.921499	-0.578918	-2.298591
C	-1.096995	0.626350	-0.084723
H	-1.931762	0.617088	0.604829
C	-2.496623	-1.604554	-0.944774
C	-3.508356	-2.265356	-0.807203
C	-0.732347	1.846476	-0.632453
C	0.499044	2.074243	-1.494370
C	0.744651	3.588224	-1.261715
H	1.416291	3.720804	-0.409076
H	1.214141	4.069221	-2.122223
C	-0.627954	4.223786	-0.927124
N	-1.379294	3.015055	-0.421262
C	-2.649440	3.097747	0.260685
C	-3.838004	2.954438	-0.486880
C	-5.059528	3.088646	0.176276
H	-5.979502	2.980037	-0.389695
C	-5.114406	3.330066	1.543566

H	-6.073861	3.428641	2.041146
C	-3.938763	3.398370	2.276517
H	-3.979395	3.533419	3.353422
C	-2.690906	3.269012	1.658172
C	1.650543	1.152753	-1.046399
H	2.105932	1.566709	-0.130245
H	2.454068	1.187334	-1.783666
C	0.213437	1.832607	-2.998825
H	1.064407	2.201159	-3.578813
H	-0.678617	2.353453	-3.347497
H	0.084683	0.778624	-3.223967
C	-1.326323	4.870461	-2.135357
H	-2.305280	5.263708	-1.851390
H	-1.463834	4.180274	-2.965457
H	-0.728082	5.711771	-2.494827
C	-0.485077	5.298271	0.159012
H	-1.455448	5.682734	0.481142
H	0.084584	6.138393	-0.246868
H	0.051123	4.927485	1.031320
C	-3.843793	2.617215	-1.959191

H	-3.020608	1.957505	-2.232388
C	-1.460854	3.262899	2.535614
H	-0.574553	2.909964	2.012237
C	2.428547	-1.466611	-0.569579
H	2.191821	-2.175969	0.234499
C	2.461865	-2.349287	-1.871230
C	3.833905	-3.037088	-1.699512
H	3.705577	-3.910248	-1.052096
H	4.229458	-3.403863	-2.651473
C	4.795813	-2.033274	-1.022190
N	3.843354	-1.001784	-0.469089
C	4.255065	-0.155778	0.616144
C	4.897427	1.075868	0.323261
C	5.307641	1.916117	1.364076
H	5.798639	2.853905	1.116542
C	5.084233	1.584729	2.690819
H	5.405570	2.247971	3.488236
C	4.442931	0.389443	2.984576
H	4.266748	0.116084	4.021238
C	4.030588	-0.492519	1.980516
C	2.408445	-1.528236	-3.171987
H	2.652028	-2.162808	-4.030204
H	3.110166	-0.694266	-3.165545
H	1.407979	-1.125280	-3.345806
C	1.347831	-3.402231	-1.914509
H	1.486637	-4.061644	-2.777821
H	0.358555	-2.949270	-2.006665
H	1.352653	-4.027983	-1.018116
C	5.779101	-1.431434	-2.046660
H	6.469103	-0.731660	-1.572160
H	5.266757	-0.912576	-2.856836
H	6.382206	-2.229017	-2.490857
C	5.656118	-2.744592	0.042380
H	6.264377	-2.034347	0.607714
H	6.337732	-3.448136	-0.445282
H	5.055217	-3.315888	0.749441
C	5.140597	1.575535	-1.082668
H	4.601342	0.988966	-1.818756
C	3.353739	-1.764522	2.442058
H	3.581092	-2.622372	1.815779
C	-0.283543	-1.357526	1.278656
C	-0.250257	-0.693187	2.514033
H	-0.131077	0.385496	2.531440
C	-0.361060	-1.386874	3.717446
H	-0.331206	-0.843302	4.657364
C	-0.499916	-2.773031	3.715755
C	-0.535065	-3.450261	2.498308
H	-0.655001	-4.529432	2.480142
C	-0.437335	-2.750091	1.297524
H	-0.503964	-3.287028	0.359959
C	-4.686679	-3.043026	-0.620899
C	-5.093705	-3.429493	0.670339
H	-4.493568	-3.126858	1.521213
C	-6.243730	-4.188539	0.852257
H	-6.542531	-4.478118	1.855048
C	-7.010808	-4.580319	-0.244742
C	-6.618181	-4.205777	-1.529460
H	-7.209614	-4.508564	-2.388110
C	-5.470351	-3.444993	-1.718921
H	-5.163757	-3.153293	-2.717507
H	-7.907498	-5.174461	-0.099656
H	-4.771139	2.104210	-2.219305
H	-3.780936	3.510412	-2.586521
H	-1.243279	4.259033	2.932342
H	-1.621525	2.605203	3.393335
H	-0.584485	-3.318190	4.650691
H	3.671357	-2.007494	3.458741
H	2.266767	-1.660920	2.464759
H	6.202719	1.540794	-1.344274
H	4.828558	2.620382	-1.173262

c) **opt-10’**

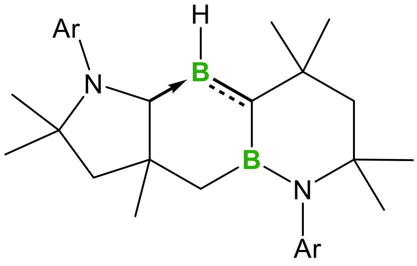

(Ar = 2,6-dimethylphenyl)

N	-2.736148	0.474587	-0.090581
H	2.000652	1.931277	-0.396824
B	1.121601	1.114409	-0.339067
N	2.741466	-0.862897	-0.071859
B	-1.326123	0.243750	-0.042944
C	3.899503	-0.025769	0.164829
C	-0.314277	1.406891	-0.175380
C	-3.676153	-0.570829	0.227765
C	4.694667	0.383750	-0.923097
C	-3.253795	1.847986	-0.442176
C	1.526455	-0.377239	-0.321031
C	4.189612	0.393487	1.476506
C	0.556925	-1.515235	-0.559672
C	-4.243253	-1.386928	-0.771331
C	5.844945	1.130792	-0.657291
H	6.464984	1.453358	-1.488443
C	-0.775296	-1.245645	0.179013
H	-0.599405	-1.385467	1.255189
H	-1.495141	-2.023482	-0.099036
C	6.187227	1.490072	0.639796
H	7.083758	2.072899	0.825649
C	-2.344277	2.888625	0.248855
H	-2.486754	2.751746	1.326296
H	-2.730148	3.889480	0.018890
C	-0.819138	2.849318	-0.039743
C	-3.992843	-0.806189	1.586368
C	5.351370	1.140850	1.691021
H	5.584934	1.468472	2.699834
C	-4.688721	2.068293	0.067348
H	-5.020788	3.069430	-0.220370
H	-4.761143	1.996926	1.153534
H	-5.390254	1.351428	-0.363673
C	1.330000	-2.756494	-0.041474
H	1.004359	-2.990058	0.975238
H	1.148524	-3.645881	-0.648930
C	2.828926	-2.377996	-0.027325
C	-3.277197	2.038659	-1.973798
H	-3.451037	3.088660	-2.226253
H	-4.079337	1.456249	-2.429585
H	-2.337382	1.732262	-2.432491
C	-5.164287	-2.375620	-0.402454
H	-5.601058	-3.000691	-1.176825

C	-0.460439	3.680653	-1.295703
H	-0.826277	4.710201	-1.200050
H	-0.878665	3.260195	-2.210238
H	0.623464	3.719509	-1.421638
C	-0.128582	3.540707	1.162515
H	-0.523962	4.553225	1.309982
H	0.948495	3.616346	1.004036
H	-0.292084	2.977240	2.085677
C	0.272241	-1.639714	-2.078171
H	-0.378976	-2.500190	-2.257683
H	1.183109	-1.783500	-2.664382
H	-0.224315	-0.744974	-2.454012
C	-5.513570	-2.577685	0.923985
H	-6.230805	-3.347528	1.191530
C	3.598472	-2.920410	-1.241657
H	3.601360	-4.012921	-1.206957
H	4.639014	-2.589213	-1.231299
H	3.148535	-2.617883	-2.186694
C	3.524915	-2.867803	1.247384
H	3.534090	-3.960899	1.247918
H	3.000065	-2.541236	2.144531
H	4.561296	-2.527390	1.304573
C	-4.915557	-1.801846	1.911066
H	-5.162015	-1.974412	2.954816
C	-3.875835	-1.270071	-2.231743
H	-3.817980	-2.261613	-2.688845
H	-4.625589	-0.705500	-2.795512
H	-2.918971	-0.772365	-2.373482
C	-3.332116	-0.029836	2.701272
H	-2.245147	-0.142842	2.683526
H	-3.530805	1.042089	2.642234
H	-3.687136	-0.378811	3.673319
C	4.329874	0.111176	-2.363742
H	4.838145	-0.772990	-2.758898
H	3.258594	-0.030205	-2.498663
H	4.628030	0.957117	-2.986481
C	3.284823	0.127740	2.657425
H	3.706105	-0.629414	3.325998
H	3.166596	1.041947	3.243792
H	2.288724	-0.195376	2.361674

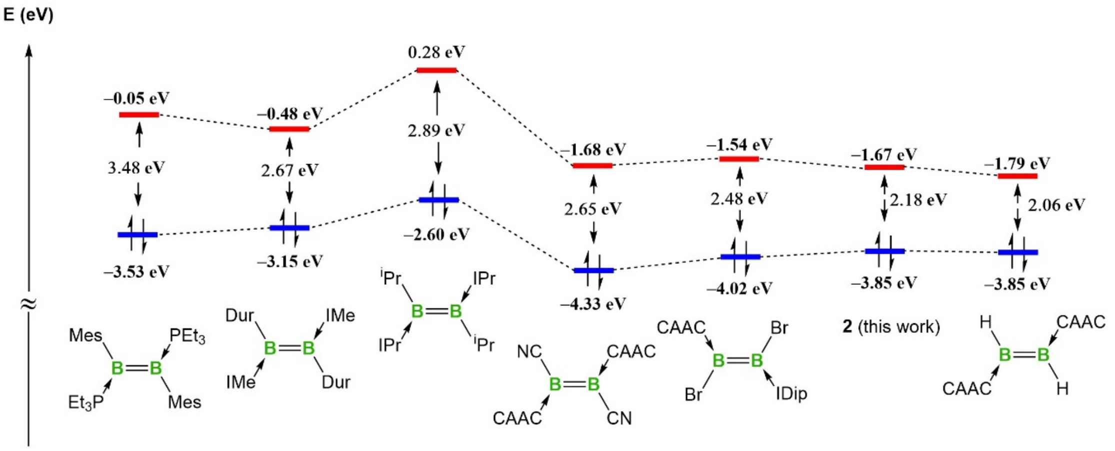

Figure S52. Energies of frontier orbitals of phosphine-, NHC-, and CAAC-supported diborenes (calculated at B3LYP/6-311G* level of theory)

Table S2. Experimental and calculated ^{11}B NMR chemical shifts of **2** (calculated at B3LYP/6-311G* level of theory).

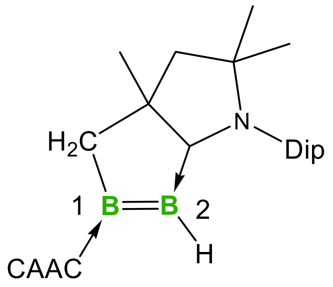

(^{11}B)[ppm]	B1	B2
exp.	48.4	43.2
cal.	51.0	44.5

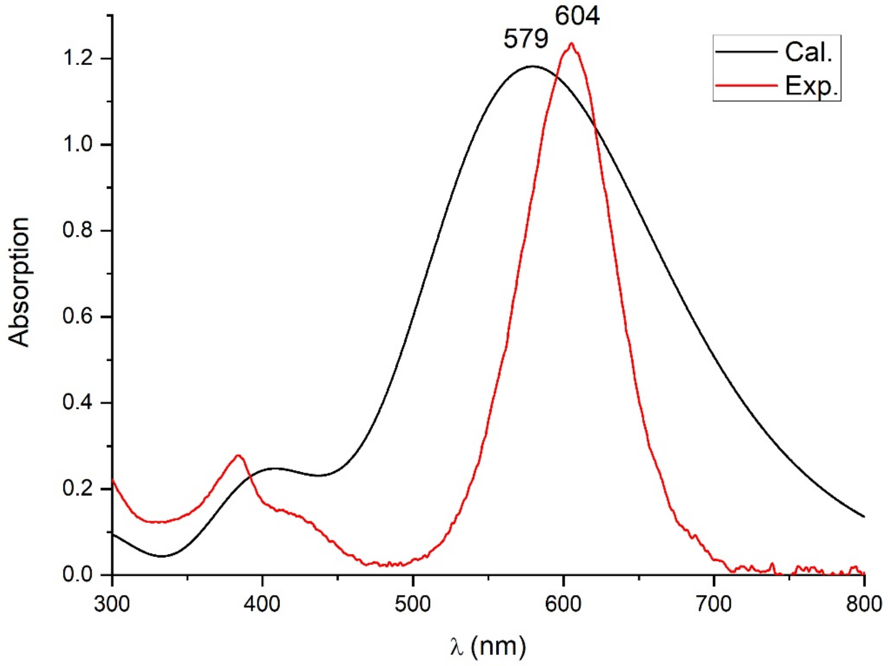

Figure S53. Experimental (red line) and calculated (black line) UV-vis absorption spectra of **2**.

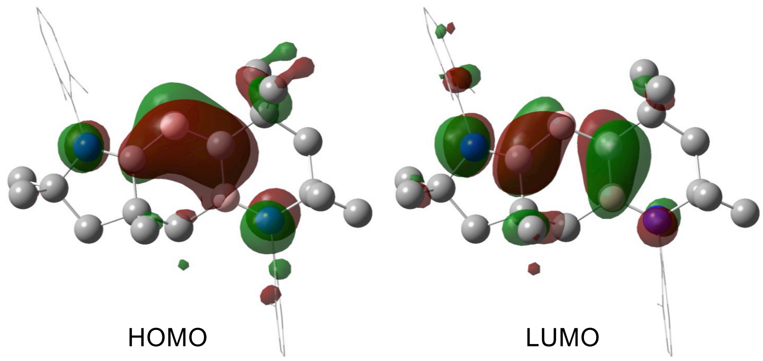

Figure S54. Plots of the HOMO and LUMO of **10'.**

4. References

[1] L. Englert, A. Stoy, M. Arrowsmith, J. H. Muessig, M. Thaler, A. Deißenberger, A. Häfner, J. Böhnke, F. Hupp, J. Seufert, J. Mies, A. Damme, T. Dellermann, K. Hammond, T. Kupfer, K. Radacki, T. Thiess, H. Braunschweig, *Chem. Eur. J.* **2019,** *25*, 8612-8622.

[2] J. Böhnke, H. Braunschweig, W. C. Ewing, C. Hörl, T. Kramer, I. Krummenacher, J. Mies, A. Vargas, *Angew. Chem. Int. Ed.* **2014,** *53*, 9082-9085.

[3] SHELXL-2014: G. M. Sheldrick, *Acta Crystallogr. Sect. C* **2015**, *71*, 3.

[4] SHELXL-97: G. M. Sheldrick, *Acta Crystallogr. Sect. A* **2008**, *64*, 112.

[5] Gaussian 09, Revision E.01, M. J. Frisch, G. W. Trucks, H. B. Schlegel, G. E. Scuseria, M. A. Robb, J. R. Cheeseman, G. Scalmani, V. Barone, B. Mennucci, G. A. Petersson, H. Nakatsuji, M. Caricato, X. Li, H. P. Hratchian, A. F. Izmaylov, J. Bloino, G. Zheng, J. L. Sonnenberg, M. Hada, M. Ehara, K. Toyota, R. Fukuda, J. Hasegawa, M. Ishida, T. Nakajima, Y. Honda, O. Kitao, H. Nakai, T. Vreven, J. A. Montgomery, Jr., J. E. Peralta, F. Ogliaro, M. Bearpark, J. J. Heyd, E. Brothers, K. N. Kudin, V. N. Staroverov, T. Keith, R. Kobayashi, J. Normand, K. Raghavachari, A. Rendell, J. C. Burant, S. S. Iyengar, J. Tomasi, M. Cossi, N. Rega, J. M. Millam, M. Klene, J. E. Knox, J. B. Cross, V. Bakken, C. Adamo, J. Jaramillo, R. Gomperts, R. E. Stratmann, O. Yazyev, A. J. Austin, R. Cammi, C. Pomelli, J. W. Ochterski, R. L. Martin, K. Morokuma, V. G. Zakrzewski, G. A. Voth, P. Salvador, J. J. Dannenberg, S. Dapprich, A. D. Daniels, O. Farkas, J. B. Foresman, J. V. Ortiz, J. Cioslowski, and D. J. Fox, Gaussian, Inc., Wallingford CT, 2013.

[6] F. Weinhold, C. R. Landis, E. D. Glendening, *Int. Rev. Phys. Chem.* **2016**, *35*, 399–440.

[7] a) I. Mayer, *Chem. Phys. Lett.* **1983**, *97*, 270–274; b) I. Mayer, *Int. J. Quantum Chem.* **1984**, *26*, 151–154.

[8] I. Mayer, P. Salvador, *Chem. Phys. Lett.* **2004**, *383*, 368–375.

[9] a) R. F. W. Bader, Atoms in Molecules: A Quantum Theory; Oxford University Press: Oxford, U.K., 1990; b) R. F. W. Bader, *Chem. Rev.* **1991**, *91*, 893–928; c) R. F. W. Bader, *J. Phys. Chem. A* **1998**, *102*, 7314–7323.

[10] G. Knizia, *J. Chem. Theory Comput.* **2013**, *9*, 4834–4843.
